# Prescription Drug Utilization and Spending by Race, Ethnicity, Payer, Health Condition, and US State

**DOI:** 10.1001/jamahealthforum.2025.2329

**Published:** 2025-08-08

**Authors:** Maitreyi Sahu, Tyler D. Wagner, Azalea Thomson, Meera Beauchamp, Jonathan D. Campbell, Sawyer Crosby, Drew DeJarnatt, Haley Lescinsky, Rayan K. Salih, Kayla Taylor, Maxwell Weil, Laura Dwyer-Lindgren, Annie Haakenstad, John W. Scott, Andy Stergachis, Utibe R. Essien, Joseph L. Dieleman

**Affiliations:** 1 Department of Health Metrics Sciences, University of Washington, Seattle; 2 Institute for Health Metrics and Evaluation, University of Washington, Seattle; 3 National Pharmaceutical Council, Washington, DC; 4 Department of Surgery, University of Washington, Seattle; 5 The Comparative Health Outcomes, Policy, and Economics (CHOICE) Institute, School of Pharmacy, University of Washington, Seattle; 6 Department of Global Health, University of Washington, Seattle; 7 Division of General Internal Medicine & Health Services Research, David Geffen School of Medicine at University of California, Los Angeles

## Abstract

**Importance:**

Achieving equitable access to medicines requires understanding of how pharmaceutical use and spending vary by race and ethnicity across the US.

**Objective:**

To quantify variation in prescription drug utilization and spending per capita and per prevalent case by race, ethnicity, health condition, payer, and US state.

**Design, Setting, and Participants:**

In this cross-sectional study, the US Disease Expenditure project was extended to incorporate disaggregation by race and ethnicity for state-level retail prescription drug utilization and spending—in addition to 143 health conditions, 38 age and sex groups, and 4 payers (Medicare, Medicaid, private insurance, and out of pocket)—across the 2019 population in all 50 states and Washington, DC. Data were analyzed from October 2023 to April 2025.

**Exposure:**

Four mutually exclusive racial and ethnic groups (Asian or Pacific Islander, Black, Hispanic, and White).

**Main Outcome and Measures:**

Outcomes include prescriptions dispensed and spending for retail pharmaceuticals. Estimates were standardized by population size, population age, and—where data permitted—by disease burden (52 conditions). Das Gupta decomposition was used to estimate the relative contribution of 3 factors (disease prevalence, prescriptions per prevalent case, and spending per prescription) on observed disparities in age-standardized per capita pharmaceutical spending.

**Results:**

In 2019, age-standardized pharmaceutical utilization and spending per person with a given disease was substantially lower than the all-population mean for Black populations, close to the mean for Hispanic populations, and often higher than the mean for Asian or Pacific Islander and White populations. These trends—particularly those for the Black population—were generally consistent across 52 health conditions but varied widely across payers and US states. The decomposition analysis for these 52 conditions showed that differences in per capita pharmaceutical spending across race and ethnicity groups were primarily explained by disease prevalence for Black populations (associated with increased per capita spending) and by utilization rates per prevalent case for Hispanic populations (also associated with increased spending). In contrast, differences in drug price or product type (spending per prescription) contributed less to observed spending disparities.

**Conclusions and Relevance:**

In this cross-sectional study, racial and ethnic disparities in medication use persisted, most notably the underutilization of medicines relative to disease burden among Black populations. These patterns varied by state, highlighting the need for local- and condition-specific approaches to advancing pharmacoequity in the US.

## Key Points

**Question:**

How do retail prescription drug utilization and spending vary by race, ethnicity, health condition, payer, and US state?

**Findings:**

In this cross-sectional study of 143 health conditions among persons in 50 states and Washington, DC, per capita pharmaceutical use was highest among White populations and lowest among Asian or Pacific Islander and Hispanic populations. However, after standardizing for age and disease prevalence (for 52 conditions with available data), prescription fills were substantially lower for Black populations relative to the all-population mean.

**Meaning:**

Disparities in medication use relative to disease burden persist—especially underutilization among Black populations—highlighting the need for targeted efforts to advance pharmacoequity.

## Teaser

This cross-sectional study aims to quantify variation in prescription drug utilization and spending per capita and per prevalent case by race, ethnicity, health condition, payer, and US state in 2019.

## Introduction

Life expectancy in the US varies considerably by race and ethnicity: in 2019, Black and American Indian or Alaska Native individuals experienced life expectancies nearly 4 and 7 years shorter than the national average, respectively.^1,2^ Prescription medications have contributed substantially to longevity, accounting for more than one-third of the overall increase in US life expectancy between 1990 and 2015.^3^ However, long-standing racial and ethnic disparities in access to medicines have been documented.^4,5,6^ These inequities can contribute to suboptimal management of health conditions and, in turn, more severe health outcomes—such as the disproportionately high rates of lower-limb amputations observed among Black individuals with diabetes.^7,8^ Accordingly, the concept of pharmacoequity, defined as “ensuring that all individuals, regardless of race and ethnicity, socioeconomic status, or availability of resources, have access to the highest-quality medications,”^4^ has gained attention in health care discussions.^9^

Ensuring equitable access to medicines begins with understanding how pharmaceutical utilization and spending vary across racial and ethnic groups, and identifying the mechanisms driving these differences. While health insurance claims data and national surveys offer valuable insights into patient access, drug pricing, and financial burden,^10^ prior studies using these datasets for disparities research have typically focused on payer-specific prescription drug spending^11,12,13^ or national-level health expenditure.^14^ To date, and to our knowledge, no analysis has systematically evaluated disparities in pharmaceutical use and spending across race, ethnicity, payer, health condition, and US state. In addition, earlier work did not account for key differences across groups—such as variation in population age structure, disease burden, and regional demographic distribution (eg, across states)—which could bias or obscure disparities. Addressing these limitations requires a more granular, standardized approach that accounts for both demographic and epidemiologic variation across racial and ethnic groups and geography.

To address these gaps, the present study leverages a novel dataset that integrates and standardizes health spending data to be representative across demographics (age, sex, race, and ethnicity), payers, 143 health conditions, and all US states, enabling a deeper level of granularity and comparability for measuring racial and ethnic disparities in access/uptake and spending on medicines.^15^ The study objectives were to (1) estimate pharmaceutical utilization and spending in 2019 for 5 race and ethnicity groups, 4 payers, and 143 health conditions across all US states; (2) standardize race-specific pharmaceutical utilization and spending data by age and, for 52 conditions with available prevalence or incidence data, by disease burden; and (3) examine how racial and ethnic differences in age-standardized pharmaceutical spending per capita are explained by variation across groups in disease burden, prescription fills per prevalent case (reflecting access, uptake, and adherence), and spending per prescription (reflecting prices and product mix).

## Methods

### Study Design

This study used outputs from the US Disease Expenditure (DEX) project, which produced estimates of US health care spending and utilization for 143 health condition categories—comprehensive of all spending—at the national, state, and county levels for 2010 to 2019.^15^ The DEX project integrated a broad set of health insurance, survey, and population data, which were scaled to align with official state-level spending totals.^16^ In addition, national- and state-level DEX estimates (across all types of care) were further disaggregated for 5 mutually exclusive racial and ethnic groups: Hispanic and (all non-Hispanic) American Indian or Alaska Native, Asian or Pacific Islander, Black, and White populations. The present study focused on 2019 retail prescription drug utilization and spending by race and ethnicity, reported per capita and per prevalent case, including a more granular assessment across 4 payers, 143 health conditions (including 52 with available disease burden data), and 50 US states and Washington, DC.

This study followed the Guidelines for Accurate and Transparent Health Estimates Reporting (GATHER),^17^ as detailed in eAppendix 5 in Supplement 1. Institutional review board approval was not required because only aggregate, nonidentifiable data were used.

### Data

#### Prescription Drug Utilization and Spending

The DEX project leveraged more than 27.3 billion public and commercial claims and survey retail pharmaceutical data from 2010 to 2019, including Medicare Part D, Medicaid, private insurance (MarketScan, Kythera, and Health Care Cost Institute), and the Medical Expenditure Panel Survey (MEPS), a national-level survey covering all payers. Details on these datasets and how they were each used are provided in eAppendix 1 in Supplement 1. Each prescription drug dataset was linked to beneficiary demographic data, including age and sex, and—where available—self-reported race and ethnicity. Race and ethnicity data were available in 4 pharmaceutical datasets, as detailed in eAppendix 1 in Supplement 1, each with varying degrees of missingness: Medicare (3%), Medicaid (18%), Kythera (88%), and MEPS (0.5%). For Medicare and Medicaid, we imputed missing race and ethnicity as described in eAppendix 1 in Supplement 1. Given the high level of missingness for Kythera, we did not perform imputation and instead relied on the 12% of records with available information. Nonetheless, the private insurance estimates included more than 20 million records with usable race and ethnicity data from Kythera, supplemented with nationally representative survey data from MEPS. We modeled estimates by race and ethnicity as described herein and in eAppendix 2 in Supplement 1.

This study included prescribed retail pharmaceuticals dispensed through community pharmacies, clinic pharmacies, mail-order pharmacies, and other outpatient settings—representing approximately 70% of total pharmaceutical spending in 2019.^18^ It excluded inpatient-administered medications, office-administered drugs (eg, chemotherapy), and institutional use such as long-term care. Data from the Indian Health Service, Tricare, and the US Department of Veteran Affairs were not available and excluded. Because approximately 64% of American Indian or Alaska Native individuals receive care through the Indian Health Service,^19^ we did not report race-specific estimates for this group except in the most aggregated result. However, these individuals were included in the calculation of all-population averages.

Spending represented total expenditure across all payers and disaggregated by payer (including out-of-pocket spending). Utilization was measured by the number of prescriptions dispensed (ie, fills).

#### Disease Burden

For 52 health conditions, state-level disease burden by race and ethnicity was modeled using race-specific mortality data.^20^ We then applied estimated incidence to mortality ratios for cancers and prevalence-to-mortality ratios for other conditions, as described in eAppendix 1 in Supplement 1. For simplicity, we refer to this modeled burden as “prevalence” or “prevalent cases” throughout the article.

#### Population Denominators

To calculate per capita estimates, we used state population data by race and ethnicity from the US Census Bureau and the National Center for Health Statistics bridged-race population estimates.^21,22^ For payer-specific estimates, we used insurance coverage by race and ethnicity from the American Community Survey and State Health Access Data Center, as detailed in eAppendix 1 in Supplement 1.^23^

### Analysis

#### Modeling

Pharmaceutical data underwent 4 modeling steps to generate comprehensive estimates of retail prescription drug utilization and spending, stratified by US state, health condition, payer, year, age category, sex, race, and ethnicity of the patient. These estimation methods are described in greater detail in eAppendix 2 in Supplement 1 and elsewhere.^15^ Briefly, these steps included (1) disaggregating data by self-reported race and ethnicity with imputation for missing values, (2) linking drugs to health conditions using a random forest model and Micromedex (Merative) records,^24^ (3) estimating utilization and spending rates through small area modeling, and (4) adjusting results to align with Centers for Medicare & Medicaid Services State Health Expenditure Accounts, which estimate prescription drug spending net of rebates and pharmacy discounts.^16,25^

#### Uncertainty and Significance

In step 3, we simulated 50 draws from a multivariate normal distribution. Calculations in step 4 were performed separately for each draw. Then, estimates were calculated as the mean of these draws with 95% uncertainty intervals (UIs) calculated from the 2.5th and 97.5th percentiles. Race-specific estimates were considered statistically significantly different from the weighted all-population mean (calculated across racial and ethnic groups) when the 95% UI of the estimate did not overlap with the weighted mean.

#### Decomposition Analysis

For 52 health conditions with existing estimates of disease prevalence by race and ethnicity (eAppendix 1 in Supplement 1), we examined possible drivers of differences in pharmaceutical spending by race and ethnicity using Das Gupta decomposition.^26,27^ Specifically, we decomposed differences between national-level spending (for the entire US population) and age-standardized race-specific spending (for each race and ethnicity group) into 3 factors: (1) disease prevalence, (2) prescriptions per prevalent case, and (3) spending per prescription. We algebraically estimated the relative contribution of each factor to these differences:

*Spending_d_*/*Population* = *Prevalent Cases_d_*/*Population* × *Prescription Fills_d_*/*Prevalent Cases_d_* × *Spending_d_*/*Prescription Fills_d_*

where *d* is the health condition (n = 52) and spending is specific to retail pharmaceuticals only. This analysis was conducted separately at national- and state-levels and for each of the 52 health conditions.

#### Standardization

All comparisons (eg, across race groups or states) were age- and sex-standardized using direct standardization with either the national population or payer-specific population as the reference group. Data were analyzed from October 2023 to April 2025 using R, version 4.4.0 (R Project for Statistical Computing), and Python, version 3.11.8 (Python Software Foundation).

## Results

Retail pharmaceutical utilization and spending by race and ethnicity are organized as follows: (1) unadjusted national patterns, (2) age-standardized results overall and by payer, (3) condition-specific trends, (4) geographic variation, and (5) decomposition of spending. Condition-specific and geographic results are age and prevalence standardized. Supplementary results are presented in eAppendices 3 and 4 in Supplement 1.

### Unadjusted National Patterns

**Figure 1.**
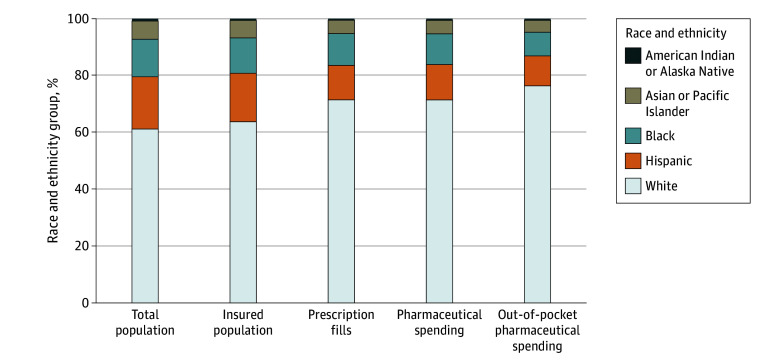
Population and Pharmaceutical Utilization and Spending by Race and Ethnicity Group, 2019 These unadjusted proportions are not age or prevalence standardized. Spending and prescription fills are estimated across all payers and US states, including Washington, DC. Population data are from the US Census Bureau. Insurance coverage data are from the State Health Access Data Assistance Center. All groups other than Hispanic exclude individuals of Hispanic ethnicity.

In 2019, an estimated 6.7 billion (95% UI, 5.9-7.5 billion) prescriptions were filled, and $331.4 billion (95% UI, $238.3-$460.2 billion) was spent on prescribed retail pharmaceuticals, including $46.8 billion (95% UI, $35.4-$62.3 billion) in out-of-pocket spending. Before adjusting for age or disease prevalence, the 61.2% of the US population identifying as White accounted for disproportionately higher insurance coverage, prescription fills, total pharmaceutical spending, and out-of-pocket pharmaceutical spending (Figure 1). In contrast, American Indian or Alaska Native, Asian or Pacific Islander, Black, and Hispanic populations each accounted for a disproportionately lower share of these outcomes relative to their population size.

### Overall and Payer-Specific Trends (Age Standardized)

**Figure 2.**
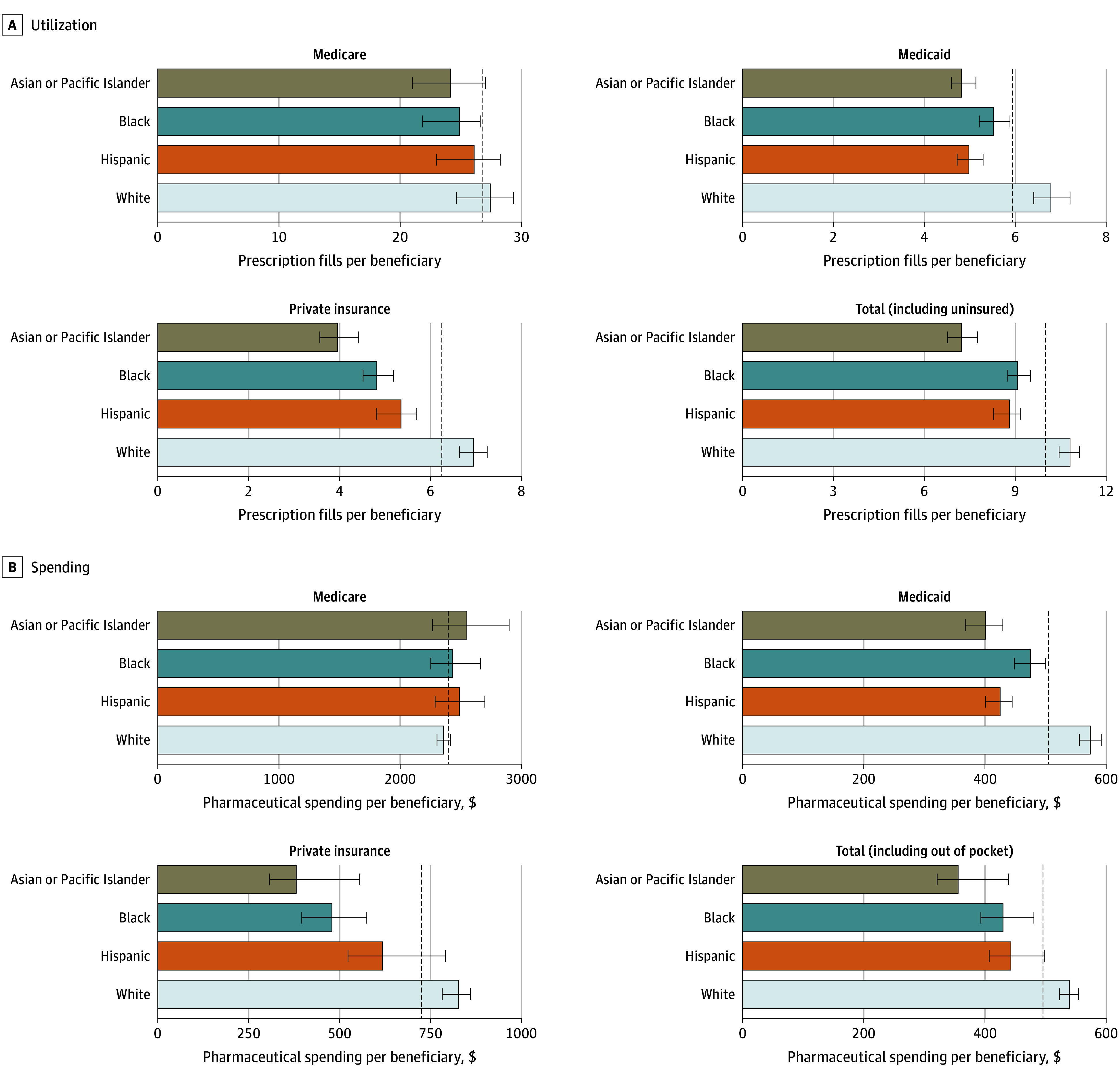
Age-Standardized Pharmaceutical Utilization and Spending by Payer Across Race and Ethnicity Groups, 2019 This figure shows the age-standardized utilization and spending per capita (for the total) and per beneficiary (for Medicare, Medicaid, and private insurance) for each race and ethnicity group. Dashed lines represent the all-population age-standardized mean prescription fills. Error bars represent 95% uncertainty intervals. All groups other than Hispanic exclude individuals of Hispanic ethnicity. The American Indian or Alaska Native population is excluded due to lack of data.

Overall, the White population had higher per capita retail pharmaceutical utilization and spending than other groups, even after adjusting for age differences (Figure 2). Private insurance accounted for 43.1% of retail pharmaceutical spending, followed by 32.3% for Medicare, 14.4% for out-of-pocket spending, and 10.1% for Medicaid. For Medicare, utilization and spending were generally consistent across groups, with the exception of Black beneficiaries, who had marginally lower utilization compared with the all-population mean. In contrast, for both private insurance and Medicaid, White beneficiaries had statistically significant higher prescription fills per person, while Asian or Pacific Islander, Black, and Hispanic beneficiaries had statistically significant lower than average fills and spending per person.

### Disease-Specific Trends (Age and Prevalence Standardized)

**Figure 3.**
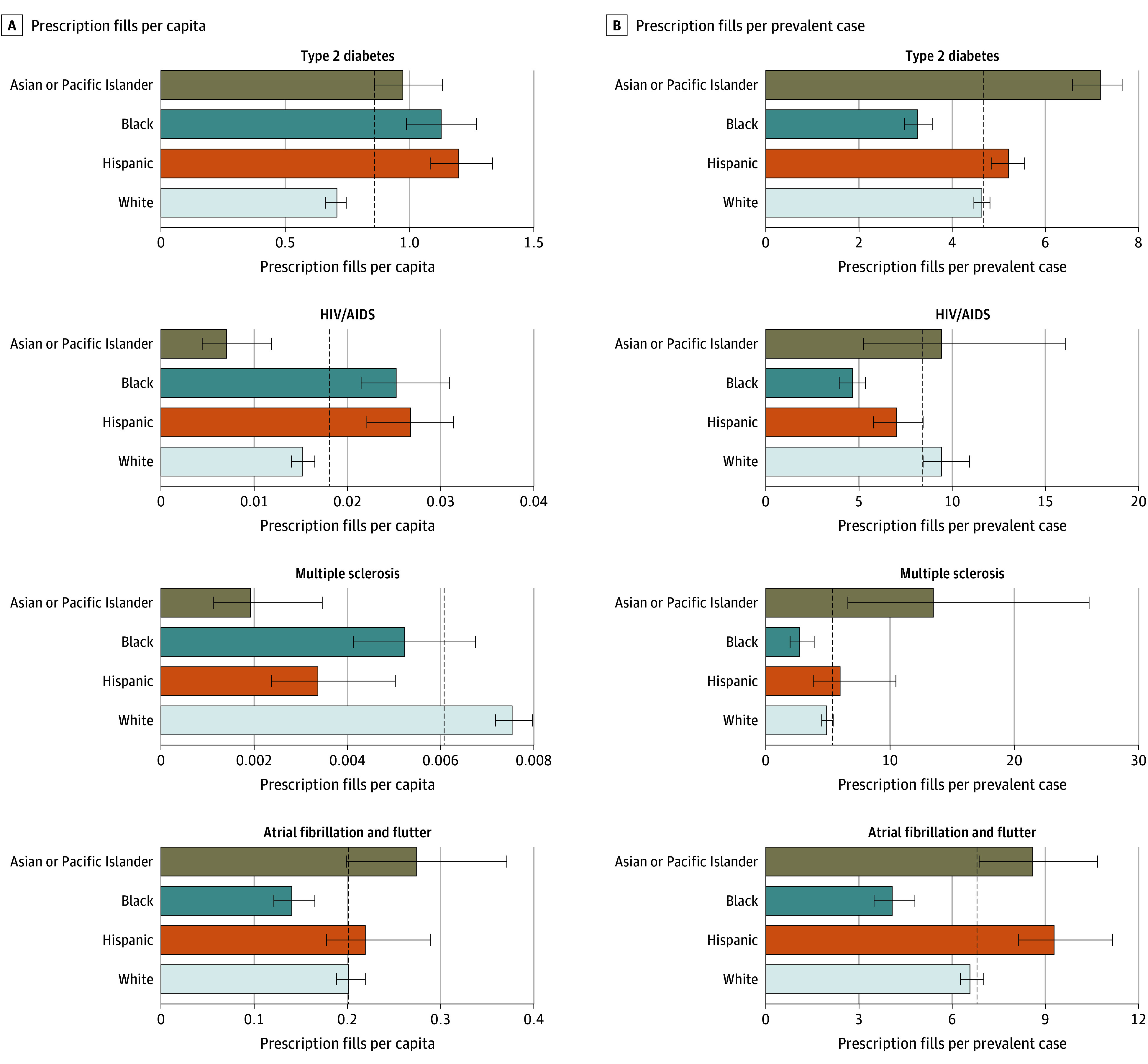
Age-Standardized Pharmaceutical Utilization per Capita and per Prevalent Case for Health Conditions With Highest Pharmaceutical Spending, 2019 This figure includes the 4 health conditions with the greatest pharmaceutical spending, of 52 with available prevalence estimates. See eFigure 1 in Supplement 1 for all 52 health conditions, including both pharmaceutical utilization and spending. Spending and utilization estimates are inclusive of preventive spending for that health condition. Dashed lines represent the all-population mean. Error bars represent the 95% uncertainty intervals. All groups other than Hispanic exclude individuals of Hispanic ethnicity. The American Indian or Alaska Native population is excluded due to lack of data.

Figure 3 and eFigure 1 in Supplement 1 present age-standardized pharmaceutical utilization and spending per capita and per prevalent case for 52 health conditions with available race- and ethnicity-specific disease prevalence estimates. Compared with per capita estimates (Figure 3A), standardizing for prevalence revealed dramatically different trends (Figure 3B), with Black individuals having substantially lower than average prevalence-standardized utilization and Asian or Pacific Islander individuals having substantially higher than average prevalence-standardized utilization. For example, for type 2 diabetes (the condition with the highest total pharmaceutical spending and utilization), Black populations had markedly fewer prescriptions per prevalent case, while Asian or Pacific Islander populations had substantially greater than average prescriptions per prevalent case. Full rankings of pharmaceutical utilization and spending—both per capita (for all 143 health conditions) and per prevalent case (for the 52 conditions with available prevalence data)—are provided in eAppendix 3 in Supplement 1.

### Geographic Variation (Age and Prevalence Standardized)

**Figure 4.**
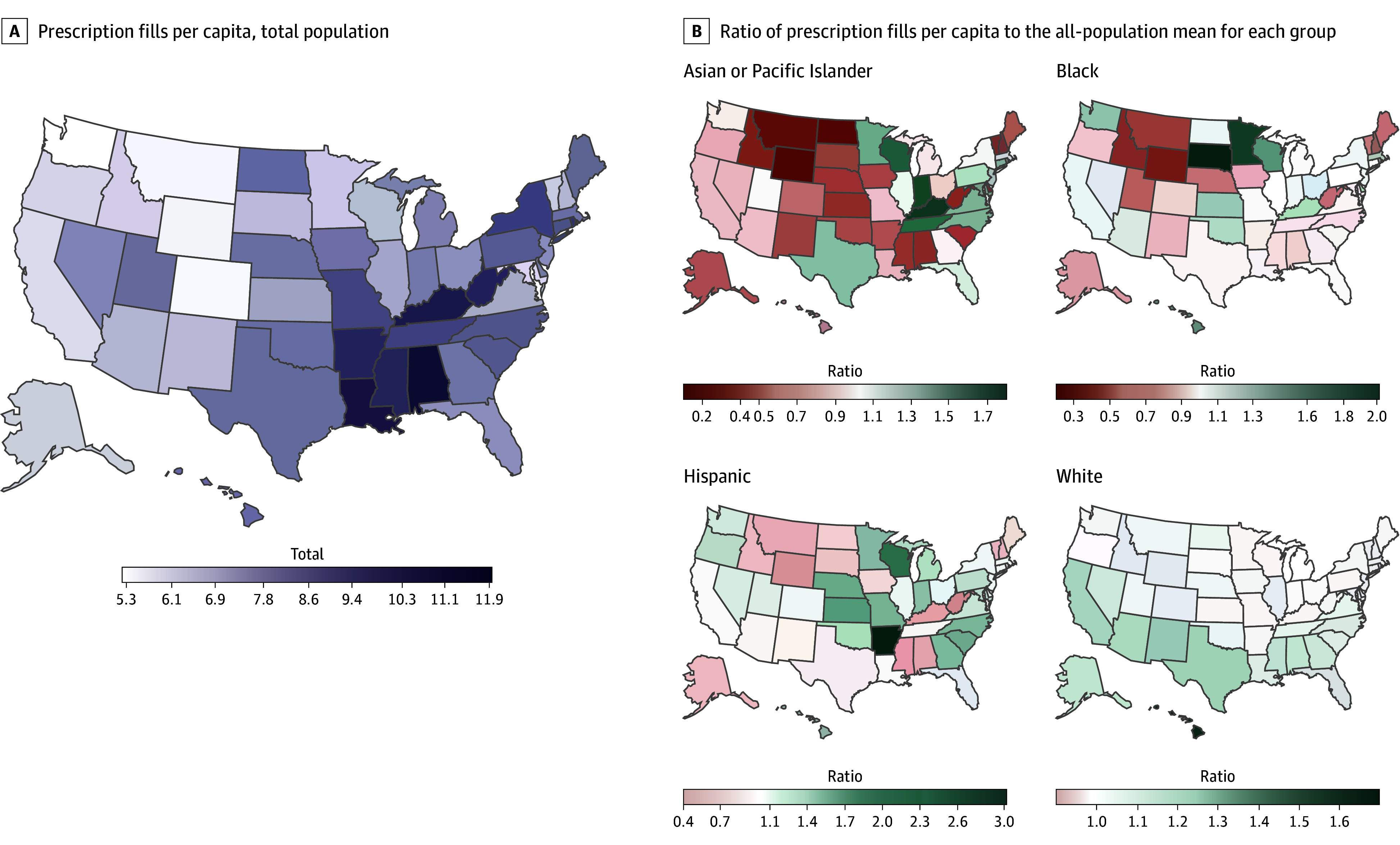
Age-Standardized Ratio of Pharmaceutical Utilization per Capita to the All-Population Mean by Race and Ethnicity Group and US State, 2019 A, Total age-standardized pharmaceutical utilization per capita for the whole population of each state. B, Ratio of race-specific age-standardized pharmaceutical utilization per capita compared to the corresponding value for the all-population mean for each state. State-level estimates and 95% uncertainty intervals for prescription fills and spending per capita are shown in the eTable in Supplement 1. All groups other than Hispanic exclude individuals of Hispanic ethnicity. The American Indian or Alaska Native population is suppressed due to lack of data but is included in the all-population means.

In 2019, the states/districts with the highest age-standardized per capita utilization of prescription drugs were Washington, DC, Alabama, West Virginia, Rhode Island, and Kentucky (Figure 4A and the eTable in Supplement 1). The states with the lowest age-standardized per capita utilization of prescription drugs were, from lowest, Washington, Colorado, Wyoming, Alaska, and California. Spatial patterns across the 50 US states and Washington, DC, differed by race and ethnicity (Figure 4B). Differences across race and ethnicity groups in age- and sex-standardized prescription drug utilization varied across US states. Pharmaceutical utilization exceeded the all-population mean in 16 states (31%) for Asian or Pacific Islander populations, 23 states (45%) for Black populations, 29 states (57%) for Hispanic populations, and 46 states (90%) for White populations. Geographic patterns varied similarly for pharmaceutical spending (eFigure 3 in Supplement 1).

### Decomposition of Pharmaceutical Spending

**Figure 5.**
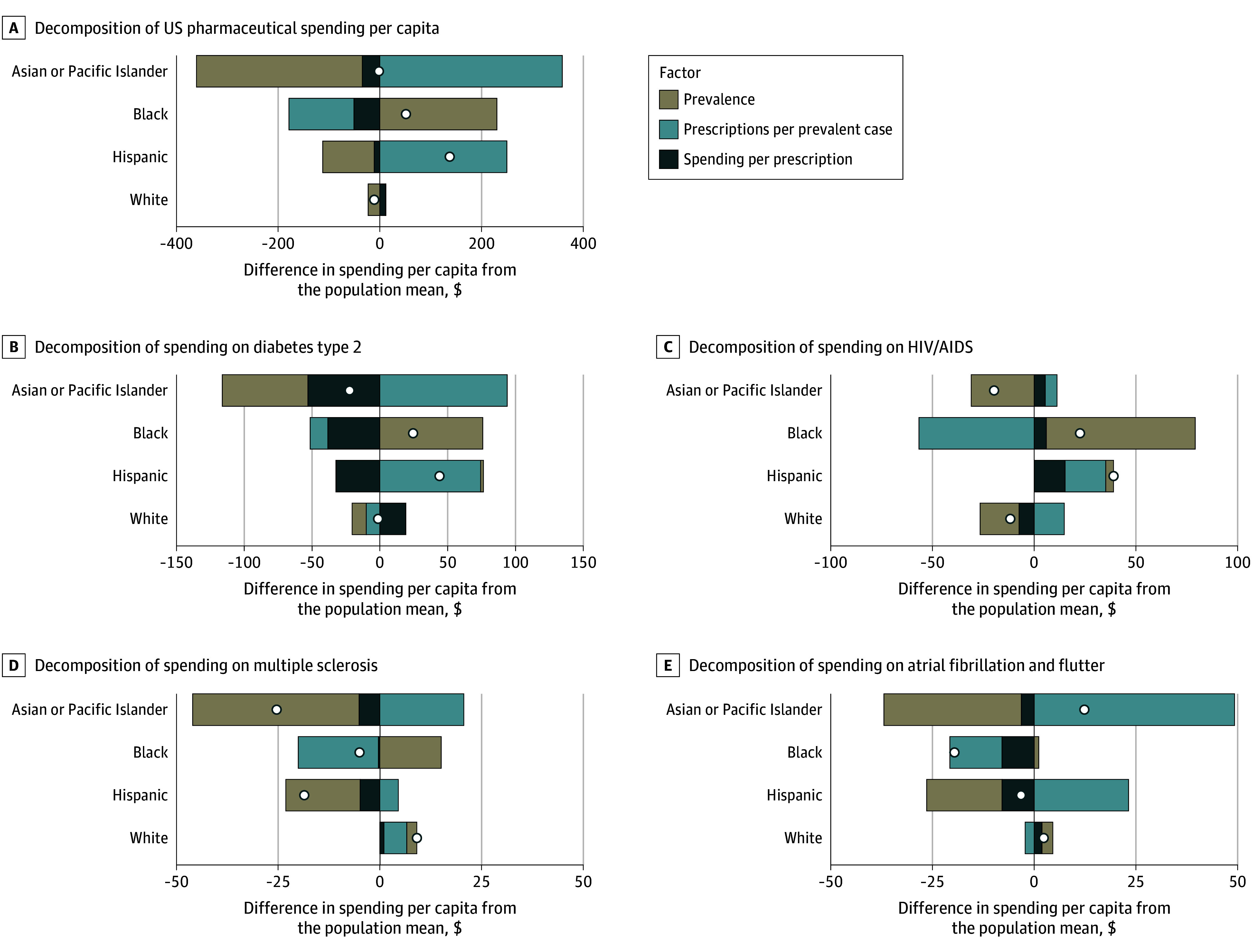
Decomposition of National Age-Standardized Pharmaceutical Spending per Capita, 2019 This figure presents the results from the Das Gupta decomposition analysis, which shows the extent to which differences in each factor (disease prevalence, prescriptions per prevalent case, and spending per prescription) contribute to differences between the age-standardized race-specific pharmaceutical spending per capita and total national-level pharmaceutical spending per capita. The open circles represent the total effect (in per capita terms), and each bar reflects the difference in age-standardized spending per capita due to each effect. The US total (A) includes 52 health conditions with available prevalence data, which account for 42% of pharmaceutical spending and 20% of prescription fills. The 4 health conditions with the greatest pharmaceutical spending (B-E) are also included. Decomposition results for the remaining 48 health conditions are presented in eFigure 2 in Supplement 1. All groups other than Hispanic exclude individuals of Hispanic ethnicity. The American Indian or Alaska Native population is excluded due to lack of data. Pharmaceutical spending is inclusive of both treatment and preventive therapies.

Figure 5 and eFigure 2 in Supplement 1 show the national-level decomposition of retail pharmaceutical spending for 52 health conditions by race and ethnicity into prevalence, prescriptions per prevalent case, and spending per prescription; these 52 conditions represent 42% of pharmaceutical spending and 20% of prescription fills. Across these 52 conditions, Asian or Pacific Islander populations had lower than average spending per capita, while the Black and Hispanic populations had higher than average spending per capita; White populations had spending per capita that was close to the all-population mean. For Asian or Pacific Islander populations, lower spending per capita appeared associated with lower prevalence, while the association of prescriptions per prevalent case was higher than average. Notably, higher spending for Hispanic populations was associated with utilization per case, while higher spending for Black populations was associated with disease prevalence. Disease-specific results for 52 health conditions are presented in eFigure 2 in Supplement 1, and state-level results across all 52 health conditions are shown in eFigure 4 in Supplement 1.

## Discussion

This study comprehensively describes inequities in retail prescription drug utilization and spending by race and ethnicity in 2019, leveraging a wealth of data to reveal heterogeneity across payers, health conditions, and US states. After standardizing for age and disease burden, substantial disparities persisted, particularly affecting the Black population, highlighting ongoing barriers to achieving pharmacoequity.

### Interpretation of Findings

These results confirm previous findings of higher per capita pharmaceutical utilization, spending, and out-of-pocket spending among White populations, associated in part with, but not completely explained by, their older age.^11,12,14^ When disaggregated by payer (Medicare, Medicaid, and private insurance), substantial inequalities emerged. While Medicare Part D showed little variation across race and ethnicity groups, Medicaid and private insurance revealed greater disparities in both spending and utilization. The present findings align with prior studies documenting higher per capita pharmaceutical utilization and spending for White individuals, as well as lower utilization and spending across payers,^14^ though we see fewer differences than previously observed in Medicare.^11,12^ Prior studies did not comprehensively account for disease burden. After standardizing for disease prevalence, we found substantially lower prescription fills per prevalent case for Black populations and substantially higher than average prescription fills per prevalent case for Asian or Pacific Islander populations. These disparities in access to medicines may help explain the more favorable health outcomes and higher life expectancy observed among Asian individuals and poorer outcomes for Black individuals.^28,29,30^

Disease-specific analyses illustrate the importance of contextualizing disparities relative to disease burden. Compared with the all-population average, the Black population had higher per capita prescription drug utilization for most health conditions examined but markedly lower prescriptions per prevalent case, suggesting undertreatment relative to disease burden. In contrast, the Asian or Pacific Islander population had lower than average per capita utilization for these conditions but higher than average per–prevalent case utilization. Moreover, while the Hispanic population had lower than average per capita utilization for some conditions, this finding disappeared after accounting for differences in prevalence. The decomposition of race-specific age-standardized spending compared with the all-population means similarly showed that Asian or Pacific Islander populations spent less per capita on pharmaceuticals due to lower prevalence; Black populations spent more per capita due to high prevalence, but utilization was low with respect to prevalence; Hispanic populations spent more per capita than average due to higher utilization; and White populations spent close to average per capita but had higher spending per prescription compared with other groups. Linking these data to disease prevalence reveals patterns and disparities that would otherwise be obscured by analyzing utilization and spending alone.

Differences in access to and uptake of medicines across racial and ethnic groups can be influenced by differential prescribing (eg, due to clinician access, clinician type visited,^31,32,33^ and discrimination^34,35,36^) and differential uptake and adherence (eg, due to insurance coverage,^37,38^ out-of-pocket cost burden,^17,28^ pharmacy access,^39^ differential drug pricing across pharmacies,^40,41,42^ and health system distrust^8^). For example, a growing body of research on pharmacy deserts has found poorer access to pharmacies in predominantly Black and Hispanic neighborhoods.^31,32,33,34,35,36,37,38,39,40,41,42,43^ Varying disease prevalence and health care access across geography may also likely contribute to the observed state-level variation in racial and ethnic differences in per capita drug utilization. Previous research has highlighted the mixed success of insurance expansions in mitigating these gaps,^44^ with Medicaid expansion improving access to prescription drugs for some populations^45,46,47^ but the Medicare Part D coverage gap being particularly disruptive to Black and Hispanic populations.^12,13^

### Policy Implications

The present findings highlight the need for tailored interventions to address racial and ethnic disparities in medication access and utilization. At the state level, prior studies have shown that Medicaid expansion demonstrated success in improving access, yet its incomplete adoption across the US limits its potential impact.^45,46,47^ Federal programs should aim to ensure equitable medication access while accommodating regional differences. While the long-term impact of the Inflation Reduction Act on racial disparities is unknown, its patient out-of-pocket caps and enhanced subsidies could further reduce financial barriers for vulnerable populations.^9,48^ Additionally, addressing structural inequities, such as pharmacy deserts and differential drug pricing, is critical to achieving pharmacoequity.^39^ Efforts to enhance equity should also prioritize preventive care and early treatment, such as prophylaxis for HIV.^49^ Increasing equitable access to therapies, such as glucagon-like peptide-1 receptor agonists and sodium-glucose co-transporter 2 inhibitors, could improve outcomes for individuals with type 2 diabetes and cardiovascular disease.^50,51^ Expanded insurance coverage, lower cost sharing, and equitable health care professional incentives could mitigate these medication access disparities.

### Limitations

This study has several limitations. First, it excluded data from the Indian Health Service, Tricare, US Department of Veterans Affairs, and incarcerated populations. Because 64% of American Indian or Alaska Native individuals access the Indian Health Service,^19^ we did not report disaggregated estimates for this group—an important omission given known disparities.^1,52^ Second, race and ethnicity data were incomplete or unavailable in several datasets, especially private insurance claims, which have known limitations.^53^ Nonetheless, these estimates (including those for private insurance) aligned with national survey data that better capture race and ethnicity.^54^ Third, small population sizes for some groups (eg, Asian and Pacific Islander individuals) contributed to wide 95% UIs. Fourth, demographic classifications were limited (eg, the Asian and Pacific Islander group aggregates heterogeneous populations^55^), and variation in data collection across states (eg, for Medicaid) may affect comparability. Fifth, drug-indication probabilities were derived from data without race and ethnicity, and we assumed these probabilities did not vary across groups; this may bias disease-specific estimates if prescribing patterns differ. Sixth, we only included retail prescriptions, excluding inpatient and in-office administered drugs, which account for roughly 30% of spending.^18^ Seventh, data ended in 2019 and did not capture the impact of the COVID-19 pandemic. Finally, because this analysis was conducted at the disease (rather than drug) level, we could not distinguish between treatment vs preventive use, account for differences in prescription length (eg, 30- vs 90-day fills), or decompose the effects of drug prices vs product mix. We also did not examine the extent to which variation reflected differences in adherence,^39^ clinician behavior, or disease severity.

## Conclusions

This cross-sectional study highlights persistent disparities in retail prescription drug utilization and spending by race and ethnicity across the US. In particular, we found that Black populations consistently had lower prescription drug utilization relative to disease prevalence. Geographic and payer-specific variations further highlight the need for targeted policy solutions. Advancing pharmacoequity will require coordinated action across federal, state, and local levels and the private sector to ensure equitable access to essential medications.

## Notes

Supplement 1. **eAppendix 1.** Data description **eAppendix 2. **Imputation and modelling methods **eAppendix 3. **National-level supplemental results **eFigure 1. **Age- and prevalence-standardized pharmaceutical spending and utilization by race and ethnicity (for 52 health conditions) **eFigure 2. **Decomposition of pharmaceutical spending per capita, for 52 conditions **eAppendix 4. **State-level supplemental results **eTable. **Pharmaceutical spending and utilization per capita by race and ethnicity (age-standardized) **eFigure 3. **State maps of pharmaceutical spending per capita (age-standardized), 2019 **eFigure 4. **State-level decomposition (aggregated across 52 health conditions) **eAppendix 5. **GATHER
eReferences

Supplement 2.
Data Sharing Statement
