# Supplementary material for: Prescription Drug Utilization and Spending by Race, Ethnicity, Payer, Health Condition, and US State
Source: JAMA Health Forum. 2025 Aug 8;6(8):e252329. doi: 10.1001/jamahealthforum.2025.2329 (PMC12334964; doi:10.1001/jamahealthforum.2025.2329)
Supplement: Supplement 1. — eAppendix 1. Data description eAppendix 2. Imputation and modelling methods eAppendix 3. National-level supplemental results eFigure 1. Age- and prevalence-standardized pharmaceutical spending and utilization by race and ethnicity (for 52 health conditions) eFigure 2. Decomposition of pharmaceutical spending per capita, for 52 conditions eAppendix 4. State-level supplemental results eTable. Pharmaceutical spending and utilization per capita by race and ethnicity (age-standardized) eFigure 3. State maps of pharmaceutical spending per capita (age-standardized), 2019 eFigure 4. State-level decomposition (aggregated across 52 health conditions) eAppendix 5. GATHER eReferences [file jamahealthforum-e252329-s001.pdf]

Supplemental Online Content

Sahu M, Wagner TD, Thomson A, et al. Prescription drug utilization and spending by race, ethnicity, payer, health condition, and US state. *JAMA Health Forum*. 2025;6(8):e252329. doi:10.1001/jamahealthforum.2025.2329

eAppendix 1. Data description
eAppendix 2. Imputation and modelling methods
eAppendix 3. National-level supplemental results
eFigure 1. Age- and prevalence-standardized pharmaceutical spending and utilization by race and ethnicity (for 52 health conditions)
eFigure 2. Decomposition of pharmaceutical spending per capita, for 52 conditions
eAppendix 4. State-level supplemental results
eTable. Pharmaceutical spending and utilization per capita by race and ethnicity (age-standardized)
eFigure 3. State maps of pharmaceutical spending per capita (age-standardized), 2019
eFigure 4. State-level decomposition (aggregated across 52 health conditions)
eAppendix 5. GATHER
eReferences

This supplemental material has been provided by the authors to give readers additional information about their work.

© 2025 Sahu M et al. JAMA Health Forum.

eAppendix 1. DATA DESCRIPTION

This section describes the underlying data for retail pharmaceutical spending and utilization, race and ethnicity, prevalence and incidence, and population denominators used for this study. As detailed in the parent study by Dieleman et al (2025),[1] datasets were aggregated, adjusted for comparability and representativeness, modeled to smooth and fill data gaps, and then scaled to state-level totals; accordingly, that study generated estimates of all types of care – including retail pharmaceutical spending – by age, sex, payer, health condition, and US county for 2010 to 2019. A follow-on study further disaggregated these estimates by race and ethnicity.[2] The present study is a deeper dive on just the retail pharmaceutical estimates, disaggregated by race and ethnicity and linked to disease prevalence.

S1.1 Retail Pharmaceutical Data

Estimates of retail pharmaceutical spending and utilization relied on a total of **27.3 billion retail prescription drug claims and encounters** from national insurance data and surveys covering all payers (private insurance, Medicare, Medicaid, and out-of-pocket payments) from 2010 to 2019. While this manuscript presents estimates for 2019 only, the estimates draw on data from the full 2010-2019 period.

Table S1.1.1: Retail pharmaceutical data used to inform our study estimates (2010-2019).

	Dataset	Source	Data Type	Payers covered	Years included	Purpose for this study	# Pharmaceutical Observations*
1	State Health Expenditure Accounts[3]	CMS	Modelled total estimates	All	2010-19	Envelopes for state-level estimates of retail pharmaceutical spending.	N/A
2	Market Scan Commercial Claims and Encounters (CCAE); Medicare Supplement (MS)[4]	IBM	Claims	Private insurance and Medicare Advantage	2010-19	Development of 'drugs to diagnosis' model; Estimation of total private utilization and spending [not race-specific].	3.4 billion<br><br>(2.72 billion CCAE + 641 million MS)
3	Medical Expenditure Panel Survey (MEPS) [5]	AHRQ	Nationally representative survey	All	2010-19	Estimation of total and out-of-pocket utilization and spending by race/ethnicity.	3.2 million *(prescribed medicines files)*
4	Medicare Part D Event (PDE) File[6]	CMS	Claims	Medicare Part D (FFS + MA)	2010, 2014-16, 2019	Estimation of Medicare utilization and spending by race/ethnicity.	6.1 billion
6	Medicaid T-MSIS Analytic Files (TAF)[7]	CMS	Claims	Medicaid	2016, 2019	Estimation of Medicaid utilization and spending by race/ethnicity.	1.7 billion
7	Kythera Payer "Complete" Dataset[8]	Kythera Labs	Claims	Private insurance	2015-19	Estimation of private utilization and spending by race/ethnicity.	13.8 billion
8	Health Care Cost Institute (HCCI) 2.0 commercial claims[9]	HCCI	Claims	Private insurance	2012-19	Estimation of total private utilization and spending [not race-specific].	2.3 billion

**See Dieleman et al. (2025) Supplement S2 for year-by-year availability of prescribed medicines data.*[1]

© 2025 Sahu M et al. JAMA Health Forum.

S1.2 Race and Ethnicity Data and Missingness

We used the five minimum, mutually exclusive groups defined by the 1977 US Office of Management and Budget (OMB) guidelines: (1) American Indian/Alaskan Native non-Hispanic, (2) Asian/Pacific Islander non-Hispanic, (3) Black non-Hispanic, (4) Hispanic, and (5) White non-Hispanic.[10] While these categorizations are limited given that recent US census data included nearly 1,500 racial and ethnic groups including over 1,200 American Indian and Alaska Native tribes and villages,[11] we were constrained by what could be reasonably captured and imputed from our datasets.

Values were recoded as follows:

- **Ethnicity:** Entries containing the term 'Hispanic' (e.g., 'Other-Hispanic,' but not 'non-Hispanic') were recoded as Hispanic.
- **Race:** After ethnicity recoding, all race entries not falling into one of the five OMB categories listed above—including 'unknown', 'other' and 'multi-race'—were recoded as 'unknown.'

This section describes the available race and ethnicity data in our four data sources which included race and ethnicity information (Medicare, Medicaid, MEPS, and Kythera) and the extent of 'unknown' values. Race and ethnicity were more completely captured in public insurance claims data (Medicare, Medicaid) and national surveys covering all payers (MEPS), but less so in our private insurance claims database (Kythera).

As described in **Appendix S2**, we imputed missing race/ethnicity information as appropriate and used these datasets to inform the race and ethnicity small area model which was then scaled to all-population estimates produced by Dieleman et al[1] which included additional non-race specific private insurance datasets (MarketScan and HCCI).

Table S1.2.1: Pharmaceutical observations and corresponding race and ethnicity information

Data Source	Payer	Race Captured?	Pharmaceutical observations [2019]	Beneficiaries [2019]	# Beneficiaries with known race/ethnicity* [2019]	% Beneficiaries with known race/ethnicity [2019]
MEPS	All	Yes	293K	28,512	27,680	97%
Medicare Part D	Medicare	Yes	609M	66,333,096	64,553,435	97%
Medicaid	Medicaid	Yes	830M	76,576,338	62,449,452	82%, but variable by state
Kythera	Private	Yes	2,887M	230,255,536	20,213,000	9%

**We define 'known' race/ethnicity as a race/ethnicity group which could be mapped to our 1977 OMB categories, not 'unknown', 'other', or 'multi-race'—all of which were mapped to 'unknown' and considered missing. Note, these are for beneficiaries with pharmaceutical claims/data only – so coverage estimates differ slightly from those described in our imputation methods (S2.1).*

© 2025 Sahu M et al. JAMA Health Forum.

A. *Medical Expenditure Panel Survey (MEPS)*

The Medical Expenditure Panel Survey (MEPS) is a nationally representative household survey, covering both insured and uninsured populations.[12] MEPS collects self-reported data on prescription drug utilization and expenditures through its prescribed medicines files. During the interview, participants are asked to self-identify their race and ethnicity using 12 race categories and 2 ethnicity categories. Except for the 'Multiple Race' category, these align directly with the 1977 OMB classification system.[10] When race or ethnicity is not reported, MEPS fills in gaps using data from prior interviews or from immediate family members, resulting in high coverage of this information. For our study, individuals identifying as 'Multiple Race' were mapped to 'Other' and considered missing.

Below, we describe the distribution of demographic characteristics (age and sex) among individuals with and without known race and ethnicity data, reporting column-wise percentages.

Table S1.2.2: Race and ethnicity data coverage in the Medical Expenditure Panel Survey (MEPS) by age group and sex (2019)

Category	With 'known' race/ethnicity	Missing ('Unknown' or 'Other') race/ethnicity
**Sex**		
Female	14332 (52%)	417 (50%)
Male	13111 (48%)	409 (50%)
**Age**		
<20	6939 (25%)	360 (44%)
20-44	8057 (29%)	247 (30%)
45-64	7103 (26%)	132 (16%)
65+	5344 (19%)	87 (11%)

Notes: (1) Percents are column-wise. (2) "'Usable' race/ethnicity" includes respondents with race and ethnicity data coded into defined categories. "Missing" includes those marked as "Unknown" and "Other," including 'Multiple Race' individuals.

B. *Medicare Part D*

Medicare Part D provides prescription drug coverage for Medicare and Medicare Advantage beneficiaries and includes detailed information on drug utilization and spending through Prescription Drug Event (PDE) files. Race and ethnicity data for Part D beneficiaries are not collected directly by the program but are instead sourced from the Medicare enrollment database, which relies on self-reported data originally collected by the Social Security Administration (SSA). Prior to 1980, the SSA captured only limited race information—typically White, Black, or Other. In 1980, the SSA revised its race and ethnicity categories to align with the 1977 OMB standards, introducing six standardized categories.[10] This change is relevant for our purposes because most Medicare beneficiaries were born before 1980, meaning their race and ethnicity information may still reflect older, less granular classifications.

In recent years, efforts to improve the accuracy of these data—such as the Research Triangle Institute (RTI) race code—have used imputation and name-based algorithms to enhance classification. We used the RTI variable in our study. Several studies have evaluated its validity.[13–15] Prior research suggests that the RTI race code performs well for non-Hispanic

© 2025 Sahu M et al. JAMA Health Forum.

Black (κ = 0.96), non-Hispanic White (κ = 0.90), and Hispanic individuals (κ = 0.87), but shows lower agreement for Asian/Pacific Islander (κ = 0.77) and American Indian/Alaska Native (κ = 0.44) groups.[13]

Below, we summarize missingness in Medicare beneficiary race and ethnicity information (RTI variable) by age, sex, and US region.

Table S1.2.3: Race and ethnicity data coverage for Medicare beneficiaries by age, sex and region (2019)

Category	With 'usable' race/ethnicity	Missing ('Unknown' or 'Other') race/ethnicity
**Sex**		
Female	35,250,023 (55%)	735,928 (41%)
Male	29,303,412 (45%)	1,043,733 (59%)
**Age**		
<20	7928 (0%)	519 (0%)
20-44	1,889,907 (3%)	88,326 (5%)
45-64	8,807,073 (14%)	145,131 (8%)
65+	53,848,527 (83%)	1,545,684 (87%)
**US Region**		
South	24,745,295 (38%)	493,795 (28%)
West	13,986,055 (22%)	488,650 (27%)
Northeast	11,702,884 (18%)	428,840 (24%)
Midwest	14,119,201 (22%)	368,376 (21%)

Notes: (1) Percents are column-wise. (2) "'Usable' race/ethnicity" includes respondents with race and ethnicity data coded into defined categories. "Missing" includes those marked as "Unknown" or "Other."

C. Medicaid

Medicaid is administered jointly by states and the federal government, and each state uses its own application form to collect race and ethnicity data. While federal guidelines require adherence to 1977 OMB minimum standards—states differ in how these questions are formatted, presented, enforced, and validated. In some cases, states treat these questions as effectively optional, resulting in substantial variation in data completeness, which can range from 50% to 100%.[16]

Some states also collect or validate race and ethnicity data through contracts with managed care organizations or by linking to patient encounter or survey data, though the methods and intensity of validation vary widely. A recent brief by the Medicaid and CHIP Payment and Access Commission (MACPAC) noted that while all state Medicaid programs report race and ethnicity data, the quality varies, with only 30 states meeting the minimum data quality standards necessary in the 2019 data.[16] These inconsistencies affect both the quality and comparability of race and ethnicity data in Medicaid.

In our analysis, we use the Transformed Medicaid Statistical Information System Analytic Files (TAF), which reflect these underlying state-level differences. Since we did not perform any

© 2025 Sahu M et al. JAMA Health Forum.

corrections to the underlying data, our estimates are constrained by the data quality of self-reported race and ethnicities.

In the following table, we summarize missingness in Medicaid beneficiary race and ethnicity information by age, sex, and US state.

Table S1.2.4: Race and ethnicity data coverage for Medicaid beneficiaries by age, sex, and US state (2019)

Category	With 'usable' race/ethnicity	Missing ('Unknown' or 'Other') race/ethnicity
**Sex**		
Female	34,835,217 (56%)	7,561,099 (54%)
Male	27,614,235 (44%)	6,565,788 (46%)
**Age**		
<20	27,564,418 (44%)	7,138,115 (51%)
20-44	18,226,044 (29%)	3,713,115 (26%)
45-64	10,513,543 (17%)	2,103,048 (15%)
65+	6,145,446 (10%)	1,172,608 (8%)
**US State**		
Alabama	383,590 (1%)	663,659 (5%)
Alaska	198,976 (0%)	27,213 (0%)
Arizona	1,302,407 (2%)	641,521 (5%)
Arkansas	663,010 (1%)	237,099 (2%)
California	12,678,100 (20%)	1,086,813 (8%)
Colorado	747,937 (1%)	483,939 (3%)
Connecticut	609,606 (1%)	352,154 (2%)
Delaware	235,574 (0%)	467 (0%)
District of Columbia	194,472 (0%)	70,858 (1%)
Florida	3,770,273 (6%)	663,448 (5%)
Georgia	1,771,118 (3%)	271,885 (2%)
Hawaii	261,392 (0%)	81,477 (1%)
Idaho	266,079 (0%)	1,255 (0%)
Illinois	2,642,154 (4%)	136,490 (1%)
Indiana	1,282,942 (2%)	152,688 (1%)
Iowa	454,419 (1%)	179,833 (1%)
Kansas	72,768 (0%)	304,390 (2%)
Kentucky	1,294,414 (2%)	250,263 (2%)
Louisiana	1,048,738 (2%)	578,020 (4%)
Maine	265,151 (0%)	26,010 (0%)
Maryland	1,134,363 (2%)	286,533 (2%)
Massachusetts	845,257 (1%)	855,420 (6%)
Michigan	2,278,587 (4%)	191,960 (1%)
Minnesota	974,553 (2%)	83,760 (1%)
Mississippi	580,452 (1%)	87,500 (1%)
Missouri	706,337 (1%)	221,113 (2%)
Montana	222,265 (0%)	30,576 (0%)
Nebraska	235,151 (0%)	19,363 (0%)
Nevada	619,594 (1%)	19,823 (0%)

© 2025 Sahu M et al. JAMA Health Forum.

New Hampshire	172,472 (0%)	23,690 (0%)
New Jersey	1,434,619 (2%)	166,405 (1%)
New Mexico	822,126 (1%)	13,741 (0%)
New York	4,383,822 (7%)	1,706,781 (12%)
North Carolina	2,084,024 (3%)	90,186 (1%)
North Dakota	86,286 (0%)	6,159 (0%)
Ohio	2,583,952 (4%)	204,052 (1%)
Oklahoma	643,939 (1%)	28,870 (0%)
Oregon	858,592 (1%)	159,494 (1%)
Pennsylvania	2,516,053 (4%)	141,412 (1%)
Rhode Island	1,327 (0%)	206,548 (1%)
South Carolina	823,518 (1%)	457,714 (3%)
South Dakota	115,843 (0%)	556 (0%)
Tennessee	13,141 (0%)	1,513,255 (11%)
Texas	3,600,919 (6%)	689,932 (5%)
Utah	175,588 (0%)	120,021 (1%)
Vermont	140,737 (0%)	28,680 (0%)
Virginia	1,347,105 (2%)	50,656 (0%)
Washington	1,586,872 (3%)	163,882 (1%)
West Virginia	359,660 (1%)	160,696 (1%)
Wisconsin	909,400 (1%)	175,866 (1%)
Wyoming	49,781 (0%)	12,760 (0%)

Notes: (1) Percents are column-wise. (2) "'Usable' race/ethnicity" includes respondents with race and ethnicity data coded into defined categories. "Missing" includes those marked as "Unknown" or "Other."

D. Kythera

Kythera is a commercial claims dataset that includes race and ethnicity information based on a combination of self-reported, provider-reported (from electronic health record data), and imputed data. Race and ethnicity are imputed by a third-party vendor using patient demographics and consumer information, and may also incorporate survey data. As typical of many commercial administrative claims databases,[17] the validation methods are not published or well described.

Race and ethnicity data were available for only 12% of enrollees; we excluded the remaining observations from race- and ethnicity-stratified analyses assuming the remaining 88% were missing at random, i.e., assuming that missingness was unrelated to individuals' actual race or ethnicity. Despite low coverage, the dataset still included 20.2 million beneficiaries with usable race and ethnicity information. The large sample size, combined with nationally representative survey data (MEPS), supported generally robust estimation for the privately insured population.

Table S1.2.5: Race and ethnicity data coverage in for private insurance beneficiaries in Kythera data by age, sex, and region (2019)

Category	With 'usable' race/ethnicity	Missing ('Unknown' or 'Other') race/ethnicity
**Sex**		
Female	11,917,642 (59%)	115,856,652 (55%)
Male	8,295,358 (41%)	94,185,884 (45%)

© 2025 Sahu M et al. JAMA Health Forum.

**Age**		
<20	2,360,588 (12%)	50,610,536 (24%)
20-44	4,829,304 (24%)	58,808,479 (28%)
45-64	6318573 (31%)	54,991,639 (26%)
65+	6,704,535 (33%)	45,631,882 (22%)
**US Region**		
South	8,759,606 (43%)	80,317,389 (38%)
West	4,121,188 (20%)	43,880,251 (21%)
Northeast	3,359,561 (17%)	41,199,194 (20%)
Midwest	3,915,305 (19%)	44,178,802 (21%)

Notes: (1) Percents are column-wise. (2) "'Usable' race/ethnicity" includes respondents with race and ethnicity data coded into defined categories. "Missing" includes those marked as "Unknown" or "Other."

S1.3 Race-Specific Prevalence and Incidence

As described in Beauchamp and Thomson,[2] we used estimates from the GBD 2021 study[18] of condition-specific incidence (for cancers and injuries) or prevalence (for all other health conditions) by state, age, and sex for 52 causes. These were combined with race- and ethnicity-specific mortality estimates from IHME[19] to generate incidence and prevalence by race and ethnicity, using estimated incidence-to-mortality or prevalence-to-mortality ratios (by state, age, sex, and race/ethnicity). We calculated spending per incident or prevalent case, referring to both as "spending per prevalent case" for simplicity.

S1.4 Population Denominators

Finally, we estimated coverage of each payer by race and ethnicity in order to generate estimates of pharmaceutical spending and utilization by payer and disaggregated by our other dimensions (age, sex, race/ethnicity, and US state). These coverage rates were specific to 6 insurance types: 1) private insurance only, 2) Medicaid only, 3) Medicare only, 4) uninsured, 5) Medicare and Medicaid, and 6) Medicare and private. For Medicare, we distinguished between Fee-For-Service coverage and managed care (Medicare Advantage) coverage. Further detail can be found in the Supplement to Beauchamp and Thomson,[2] but in brief, several sources were used to determine the fraction of each race/ethnicity that comprised the all-population payer denominators (the methods of which can be found in pages 81-84 of *Dieleman et al, 2025*[1]).

To generate the race fractions for the uninsured, privately insured, and those with Medicare and private insurance, we extracted data for years 2009 to 2019 from the American Community Survey (ACS)[20] and data for years 2008 to 2019 from the State Health Access Data Assistance Center (SHADAC).[21] Data were proportionally split into the 19 age bins and 2 sexes in our final estimates using the all-race population denominators as a reference. Smooth time trends between 2000 and 2019 were ensured using loess and linear regression. To generate race fractions for Medicaid and Medicare, we used the enrollment data to create a set of race proportions by age group and sex. We fit a loess regression for years 2009 to 2019 to create a complete time trend and then a linear regression to predict estimates back to 2000. All race fractions were then multiplied by all-population payer-specific totals.

© 2025 Sahu M et al. JAMA Health Forum.

eAppendix 2. IMPUTATION AND MODELLING METHODS

The steps involved in the estimation of pharmaceutical utilization and spending are described in detail in ‘Tracking US County Health Spending by Health condition, 2010-2019,”[1] with key elements summarized here. State-level estimates of prescription drug utilization and spending were then disaggregated by race and ethnicity as described in further detail here and in a forthcoming paper.[2]

Figure S2.0: Overview of datasets and steps used to generate estimates of pharmaceutical spending and utilization by race and ethnicity

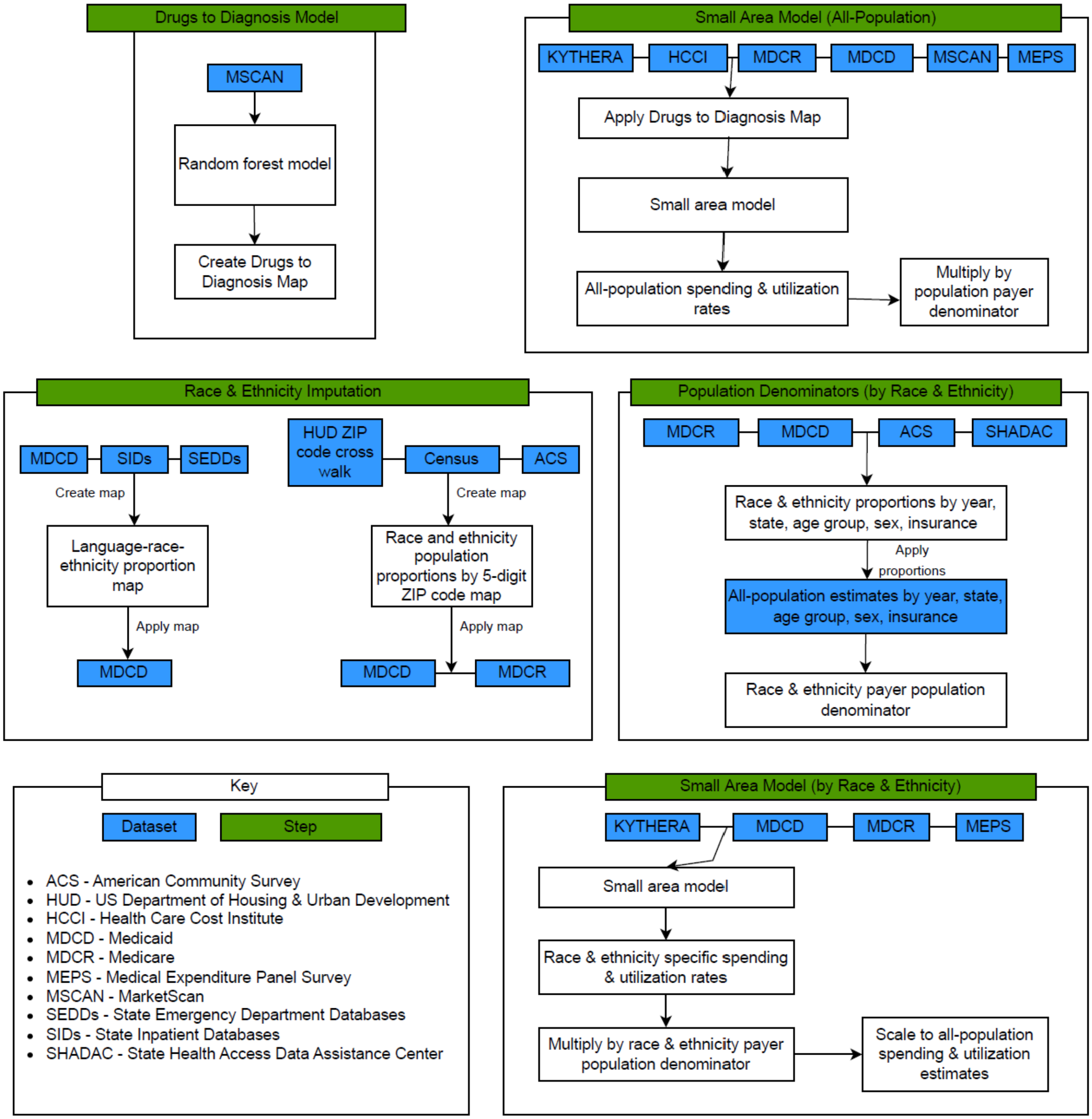

© 2025 Sahu M et al. JAMA Health Forum.

S2.1 Race and Ethnicity Imputation

Where race and ethnicity were missing, we applied imputation methods consistent with recommendations from the Institute of Medicine (US) Subcommittee on Standardized Collection of Race/Ethnicity,[22] as briefly outlined in **Appendix S2 Imputation and Modelling Methods** and discussed in greater detail in a forthcoming paper by Beauchamp and Thomson.[2]

Missingness of race and ethnicity information across our datasets is described in Section S.1.2. Overall, 12.7% of records were missing race/ethnicity information across MEPS, Medicaid, and Medicare Part D (RTI);[2] the proportion of missing data was lower for retail pharmaceutical data only (Table S1.2.1). Race and ethnicity were imputed for Medicare and Medicaid only. For Kythera, which had limited race/ethnicity coverage (~12%; see S1.2D), we used available data to inform distributions, scaling them to payer-specific totals estimated from Kythera, MarketScan, and HCCI (Section S2.4).

Table S2.1.1: Dataset and whether imputation was performed

Dataset	Race/Ethnicity Imputed?	Notes
MEPS	No	High completeness; no imputation performed.
Medicare (RTI)	Yes	Race/ethnicity imputed.
Medicaid	Yes	Race/ethnicity imputed.
Kythera	No	Poor coverage (~12% had race/ethnicity). Assumed missingness at random and dropped all data from beneficiaries with unknown race/ethnicity.

To impute missing race and ethnicity data for Medicare and Medicaid, we used a two-step modeling process. These methods are described in more detail in the forthcoming manuscript 'Tracking US State Health Spending by Race and Ethnicity, 2010-2019,"[2] and are summarized here. The imputation approach drew on not only pharmaceutical data but also the State Inpatient Databases (SIDs) and State Emergency Department Databases (SEDDs), which include race and ethnicity information.

A. Language to race and ethnicity mapping

Using Medicaid as well as SIDs and SEDDs, which record both the patient's primary language and their race and ethnicity, we generated a map from language to race and ethnicity. All native languages present in our data (Achinese, Aleut, Algonquian languages, Apache languages, Central American Indian languages, Choctaw, Dakota, Guarani Native American Language, Haida, Inuktitut, Inupiaq, Mi'kmaq, North American Indian languages, Navajo, Quechua, Salishan languages, Siouan languages, Zuni) were assigned to American Indian/Alaska Native. In addition, a primary language of Spanish was always assigned to Hispanic ethnicity. Then, all remaining data containing valid language values other than English and valid race values were used to generate a set of proportions of each race and ethnicity within a language. This map was validated using Medicaid, SIDS and SEDDS data where both language and race and ethnicity were known.

© 2025 Sahu M et al. JAMA Health Forum.

Table S2.1.2: Dataset and whether primary language was included

Dataset	Primary Language Included?	% of total observations with imputed race based on primary language	% observations with imputed matched self-report race/ethnicity
Medicaid	Yes	2.13%	99.2%
Medicare	No	N/A	N/A
SEDDs	Yes	0.03%	93.5%
SIDs	Yes	0.03%	88.5%

B. Probabilistic zip code level imputation

The Decennial Census and American Community Survey (ACS) report population data by race and ethnicity at the census tract level.[23,24] We applied the U.S. Department of Housing & Urban Development (HUD) zip code crosswalk files[3] to the census and ACS data to map race population proportions from census tracts to 5-digit zip code geographic areas. Medicaid and Medicare beneficiaries with outstanding missing race information after language imputation but known 5-digit ZIP code were probabilistically assigned a race based on the racial composition of the population in that ZIP code, i.e. each individual was randomly assigned a race according to the ZIP code's population distribution. This map was validated using Medicaid, Medicare, SIDs, and SEDDs data where both zip code and race and ethnicity were known.

Table S2.1.3: Dataset and whether zip code was available

Dataset	Zip code included?	% of total observations with imputed race based on zip code	% observations with imputed matched self-report race/ethnicity
Medicaid	Yes	11.4%	57.2%
Medicare	Yes	1.6%	76.3%
SEDDs	Yes	8.3%	60.7%
SIDs	Yes	11.0%	68.6%

© 2025 Sahu M et al. JAMA Health Forum.

S2.2 Drugs to Diagnosis Model

The methods for linking drugs to their corresponding indications are published as part of the methods appendix in a recent study, 'Tracking US County Health Spending by Health Condition, 2010-2019," in **Section S3.1H National Drug Codes (NDCs) for pharmaceuticals –** the excerpt from this appendix is provided below with minimal edits.[1] A forthcoming paper from Sahu and Weil (focused on off-label prescribing) further describes the internal and external validation as well as the model section process.

A. National Drug Codes (NDCs) for pharmaceuticals

Prescription drug claims data contain 11-digit FDA-assigned National Drug Codes (NDCs) identifying pharmaceuticals by the manufacturer, product formulation, and package details, but not the diagnoses for which they are prescribed. We probabilistically mapped NDCs to the list of health conditions outlined in S3.1E in a multi-step modeling process as follows: (1) using MarketScan retail pharmaceutical and outpatient visit data, we linked prescription drug claims to the most recent outpatient visit occurring up to 30 days before the pharmaceutical claim, (2) we restricted the data to on- and off-label uses from the Merative Micromedex pharmaceutical compendium, which we also manually mapped to our 143 conditions, and (3) we used a random forest classification model to predict one of 143 on- and off-label uses given the generic drug name, for 8 age/sex groups and leveraging drug details including dosage, package size, and route of administration, and (4) we probabilistically applied the model to our full set of pharmaceutical data containing NDCs including Medicare, Medicaid, private, and out-of-pocket spending.

B. MarketScan data processing

To develop our training dataset, we first linked MarketScan pharmaceutical claims and diagnoses from outpatient visits as follows. The pharmaceutical claims included NDCs, which we linked to their corresponding generic drug names using Market Scan's Redbook. We restricted the data to only original prescriptions (not refills), to capture claims likely to be associated with a recent provider visit. Using the beneficiary ID, prescription drug claims were then linked to the most recent outpatient visit occurring within 30 days prior to the date of the pharmaceutical claim; this 30-day window aligns with the duration often allotted to pharmacies for reimbursement by insurers. The outpatient visit data contained up to 4 diagnoses, recorded using ICD codes, per visit. These ICD codes were then comprehensively mapped to the 143 health conditions using a process described in detail in Dieleman et al.[1] This resulted in a dataset with pharmaceutical claims linked to up to 4 diagnoses (i.e., up to 4 rows per pharmaceutical claim), but since diagnoses were not necessarily the condition for which the drug was prescribed, this dataset was insufficient for our purposes because it included a large number of commonly occurring co-morbidities (e.g., insulin being linked to both Type 2 Diabetes and hypertension).

Table S2.2.1: MarketScan data used to inform classification model

Pharmaceutical observations [2019]	Beneficiaries [2019]
240 million<br>(196 Commercial Claims & Encounters + 44 Medicare Advantage)	20,159,606

© 2025 Sahu M et al. JAMA Health Forum.

C. Restriction using the Merative Micromedex pharmaceutical compendium

To restrict our training dataset to only plausible drug-cause pairs, we used the Merative Micromdex database which is a pharmaceutical compendium that describes both the on- and off-label indications for each drug's generic name. Because the indications are given in plain English language rather than any standardized coding (such as ICDs), we had to manually map indications listed in Micromedex to the set of 148 health conditions in our analysis. We conducted this mapping for 755 drugs that covered >95% of total spending, utilization, and out-of-pocket spending in the MarketScan data. Drug-cause pairs were then further restricted to plausible demographics by age and sex. The resulting dataset included 160,329,831 rows of plausible drug-cause pairs on which we trained our model. Figure 2 gives a full description of the data processing and restriction steps to generate the final training and test datasets for the model.

Figure S2.2.1: MarketScan pharmaceutical (RX) data processing

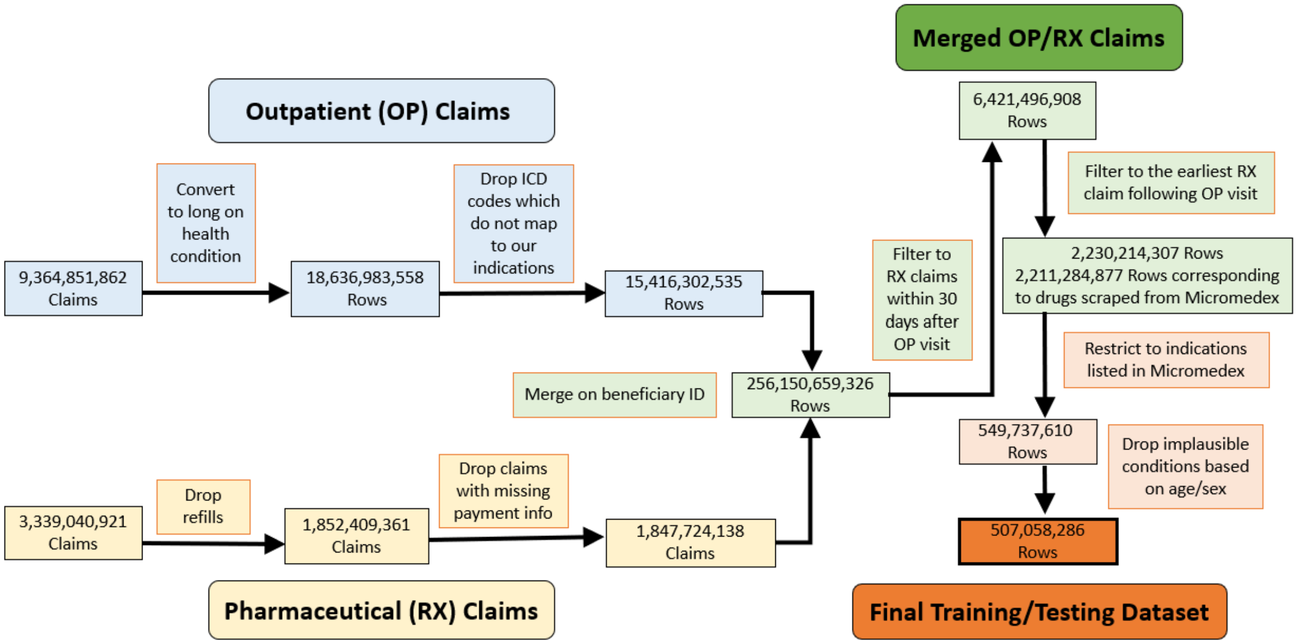

D. Random forest model

To predict the probability of each of our 143 health conditions for each drug, we trained a random forest model with 1000 estimators. We ran a separate model for each of our 755 drugs and 8 age/sex groups, and included as covariates the package size, dose strength, the route of administration, and the total spending. For the remainder of the ~8500 drugs covering <5% of spending, utilization and out-of-pocket spending, we applied the same model to the dataset that included restrictions by age and sex but not by indication. After modelling, the output probabilities from the random forest models were used to estimate the likelihood of a NDC being prescribed to a condition. Probabilities for a condition were computed as the average prediction

© 2025 Sahu M et al. JAMA Health Forum.

probability for that condition across all trees, where the predicted probability for a single tree is the fraction of leaves that result in the condition. Full details of the model selection and validation to choose this model are described in a forthcoming paper from Sahu and Weil.

E. Model Application

Finally, these probabilities (which were specific to generic drug name and age/sex group) were then used to assign health conditions across our prescription claims data from Medicare, Medicaid, HCCI, Kythera, and MEPS; specifically, each pharmaceutical claim with an NDC was probabilistically assigned to a single health condition from the set of possible conditions associated with that code.

S2.3 Small Area Modelling

Our all-population pharmaceutical spending and utilization estimates (and associated uncertainty) were generated using a small area model as described in the detailed methods appendix of the published study 'Tracking US County Health Spending, 2010-2019."[1] The full model specification is available on pages 69-70 of the published appendix. The model was implemented using Template Model Builder (TMB), an open-source R package designed for fitting nonlinear random effects models.[25] Race-specific estimates were generated using a similar modeling approach, with an additional level of disaggregation, as detailed in the forthcoming study 'Tracking US State Health Spending by Race and Ethnicity, 2010-2019."[2] The full model specification, which is similar to that for the all-population estimates but includes an additional term for race/ethnicity, is described in the methods appendix of Beauchamp and Thomson et al.[2]

S2.4 Post-Model Steps and Scaling

Two steps were required to ensure that our race/ethnicity results summed to all-population estimates, which are scaled to the total expenditures reported by the government State Health Expenditure Account (SHEA). First, we needed to ensure that for every all-population estimate combination,[1] (geography-type of care-primary payer-payer-sex- health condition) we had at least one race-specific estimate, such that all race-specific estimates summed to the total population. This step was referred to as post model imputation, and more detail is available in the Supplement to *Tracking US State Health Spending by Race and Ethnicity, 2010-2019*.[2]

The second step, used for generating all-population estimates, ensured that all estimates summed to the total expenditures reported by SHEA (state, year, payer, and category—"type of care"—specific). SHEA data was downloaded from the official Centers for Medicare & Medicaid Services website[26] and converted to 2019 currency. Our state-level retail pharmaceutical expenditure envelopes were the sum of two categories in the SHEA data, 'Prescription Drug Expenditures' and 'Other Non-Durable Medical Products Expenditures.' The SHEA rebate adjustment uses Medical Loss Ratio data to adjust retail pharmaceutical sales data.[27]

© 2025 Sahu M et al. JAMA Health Forum.

S2.5 Validation

Given the substantial missingness in race and ethnicity data and our reliance on imputation in some cases, we conducted sensitivity analyses to test the robustness of our findings: (1) we re-estimated results without imputing race or ethnicity for any individuals (note: imputation was used only for Medicare and Medicaid data); (2) to assess whether our private insurance estimates were biased due to poor data quality in the Kythera dataset, we compared our national-level estimates to those from MEPS, a nationally representative survey (note: both Kythera and MEPS data were used to generate our private insurance estimates).

A. Imputation sensitivity analysis

We conducted a sensitivity analysis of our imputation methods using language and zip information by creating estimates with and without imputation of missing race and ethnicity information. We found a negligible difference between the two.

Figure S.5.1: Sensitivity analysis national pharmaceutical spending per capita variation by race and ethnicity, 2019

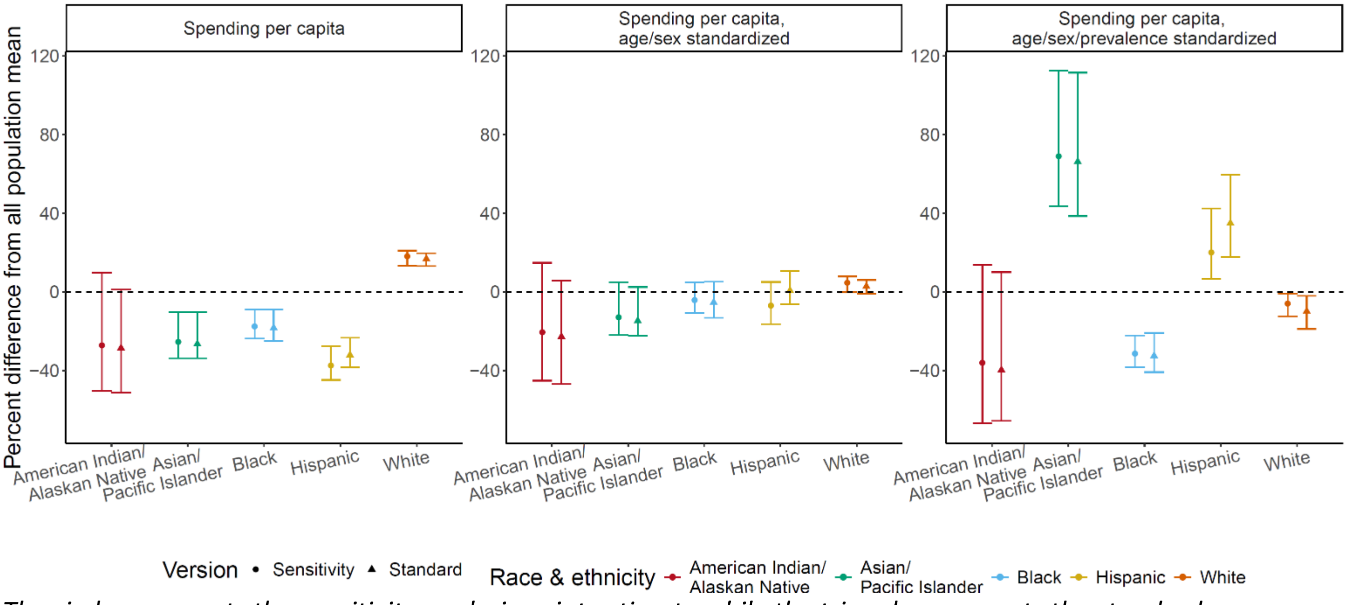

The circle represents the sensitivity analysis point estimate while the triangle represents the standard analysis. The facets from left to right represent the unstandardized, age/sex standardized, and age/sex/prevalence standardized spending. The far-right panel includes spending for only the 52 causes for which prevalence information was available while the left and middle panels include spending for all 143 causes. The dashed line indicates the all-population weighted mean, where each data point is the percent difference from that value. Error bars indicate 95% uncertainty intervals (UIs). UIs were based on completing the analysis on 50 independent samples of the underlying data.

B. Private insurance benchmarking

Finally, to assess the reasonableness of our private insurance estimates—which draw from Kythera data with substantial missingness as well as the nationally representative Medical Expenditure Panel Survey (MEPS)—we benchmarked our national-level estimates against age-

© 2025 Sahu M et al. JAMA Health Forum.

and sex-standardized MEPS data for privately insured individuals for 2019. MEPS is nationally representative across race/ethnicity groups, and allows for national disaggregation of pharmaceutical utilization and spending by both payer type and race/ethnicity. Prior literature using MEPS, suggests that even among privately insured individuals, White beneficiaries use health care at a higher rate than other groups.[28]

As shown in **Figure S.5.2**, our modeled estimates align with MEPS-derived estimates for age- and sex-standardized prescription fills per privately insured beneficiary across racial and ethnic groups. In **Panel A**, we present nationally representative, age- and sex-standardized estimates of 2019 prescription fills and pharmaceutical spending per beneficiary using MEPS, applying survey weights and accounting for the complex survey design. **Panel B** is from the main paper Figure 2, using our study estimates (DEX). Both demonstrate a similar pattern: White individuals had the highest fill rates, Asian/Pacific Islander individuals the lowest, and Hispanic and Black individuals had somewhere in between. Although absolute values and exact rank ordering differ slightly, the general alignment supports the validity of our model-based estimates despite limitations in the underlying Kythera dataset.

Figure S.5.2: Benchmarking prescription drug utilization per beneficiary for private insurance using the Medical Expenditure Panel Survey (MEPS), 2019

A) MEPS-derived estimates, age-standardized

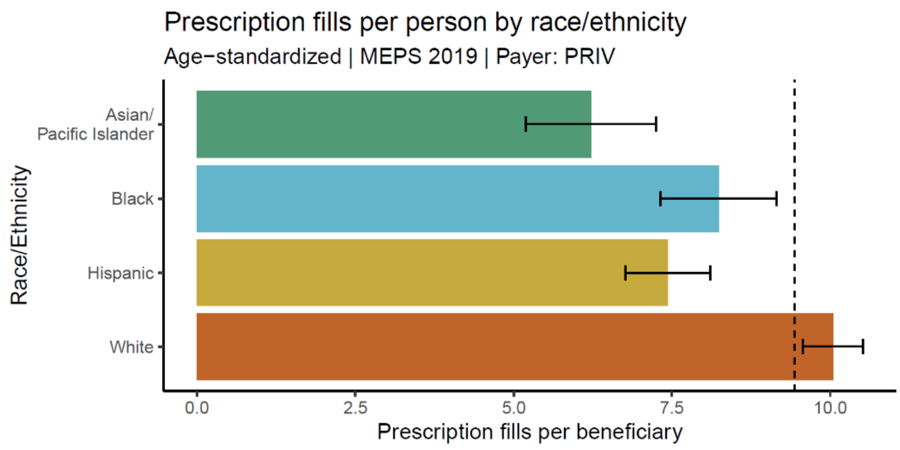

B) DEX estimates, age-standardized (main paper Figure 2)

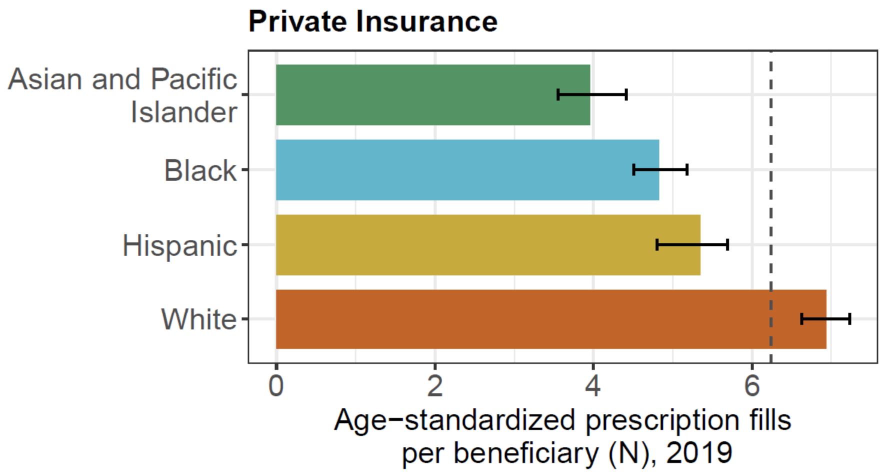

© 2025 Sahu M et al. JAMA Health Forum.

eAppendix 3. NATIONAL-LEVEL SUPPLEMENTAL RESULTS

A. Table S3.1: *Pharmaceutical spending and utilization per capita by race and ethnicity (age-standardized), for 143 health conditions*

Here we present spending and utilization per capita by race/ethnicity, for each of 143 health conditions included in the US Disease Expenditure (DEX) project. Health conditions are ranked by total retail pharmaceutical spending. All groups other than Hispanic are non-Hispanic ethnicity. The mean values across 100 draws are presented in the top row, with the 95% Uncertainty Interval below.

Rank (total drug spending)	Health Condition	Spending per capita (US$)				Prescription fills per capita (n)			
		Asian and Pacific Islander	Black	Hispanic	White	Asian and Pacific Islander	Black	Hispanic	White
1	Diabetes mellitus type 2	149.1	193.3	213.7	167.8	0.97	1.13	1.2	0.71
		(130.2, 177.2)	(173.6, 221.3)	(194.2, 236.5)	(160.6, 176)	(0.9, 1.1)	(1, 1.3)	(1.1, 1.3)	(0.7, 0.7)
2	Skin and subcutaneous diseases	59.1	45.6	63.7	83.1	0.42	0.35	0.48	0.43
		(46.5, 79.5)	(37, 54.6)	(51.4, 76.2)	(78.4, 86.8)	(0.4, 0.5)	(0.3, 0.4)	(0.4, 0.6)	(0.4, 0.4)
3	Endocrine, metabolic, blood, and immune disorders	38.8	27.7	45.8	69.6	0.38	0.26	0.45	0.47
		(28.8, 52.4)	(24.8, 31.3)	(39.9, 51.6)	(67, 72.4)	(0.3, 0.5)	(0.2, 0.3)	(0.4, 0.5)	(0.5, 0.5)
4	Treatment of hypertension	42.1	74.3	37.6	40.5	1.07	1.41	0.9	0.8
		(36.8, 48.7)	(67, 80)	(34.2, 42.5)	(39.1, 41.7)	(0.9, 1.2)	(1.3, 1.5)	(0.8, 1)	(0.8, 0.8)
5	Other musculoskeletal disorders	40.7	31.7	32.6	47.3	0.23	0.22	0.21	0.22
		(32.3, 53.8)	(25.8, 38.7)	(27.2, 39.4)	(45.2, 49.5)	(0.2, 0.3)	(0.2, 0.2)	(0.2, 0.2)	(0.2, 0.2)
6	HIV/AIDS	18.3	60.5	76.3	26.1	0.01	0.03	0.03	0.02
		(7.2, 45.3)	(42, 94.6)	(38, 124.4)	(12, 38.6)	(0, 0)	(0, 0)	(0, 0)	(0, 0)
7	Multiple sclerosis	8.9	29.3	15.7	43.7	0	0.01	0	0.01
		(4.1, 17.2)	(19.2, 47)	(8.1, 27.6)	(38.6, 48.8)	(0, 0)	(0, 0)	(0, 0)	(0, 0)
8	Atrial fibrillation and flutter	58.4	24.9	41.4	47.4	0.27	0.14	0.22	0.2
		(39.9, 79.7)	(21.2, 31)	(32.8, 55.2)	(43.4, 51.8)	(0.2, 0.4)	(0.1, 0.2)	(0.2, 0.3)	(0.2, 0.2)
9	Asthma	16.2	20.5	25.8	30.5	0.12	0.17	0.19	0.2
		(13.4, 20.1)	(18.8, 22.5)	(22.8, 30.3)	(29.6, 31.6)	(0.1, 0.1)	(0.2, 0.2)	(0.2, 0.2)	(0.2, 0.2)
10	Rheumatoid arthritis	9.3	14.3	31.8	28.3	0.02	0.03	0.05	0.05
		(4.9, 18.6)	(11.1, 20.4)	(21.8, 38.7)	(26.2, 31.1)	(0, 0)	(0, 0)	(0, 0.1)	(0, 0)
11	Treatment of hyperlipidemia	25	18.8	24.1	22.9	0.43	0.34	0.41	0.35

© 2025 Sahu M et al. JAMA Health Forum.

		(21, 28.8)	(16.8, 21.8)	(21.8, 27.6)	(21.7, 23.9)	(0.4, 0.5)	(0.3, 0.4)	(0.4, 0.4)	(0.3, 0.4)
12	Chronic obstructive pulmonary disease	25.8	18.3	22	28.9	0.13	0.12	0.14	0.12
		(17.7, 36)	(16.1, 21.1)	(17.3, 28.5)	(27.9, 30.1)	(0.1, 0.2)	(0.1, 0.1)	(0.1, 0.2)	(0.1, 0.1)
13	Inflammatory bowel disease	17.9	12.2	15.8	24.3	0.03	0.03	0.04	0.05
		(7.5, 41.3)	(7, 18.6)	(9.9, 24.6)	(20.8, 27)	(0, 0)	(0, 0)	(0, 0)	(0, 0.1)
14	Leukemia	31.5	28	25.7	15.5	0.01	0.01	0.01	0
		(16, 62.6)	(18.5, 40.6)	(15.2, 39)	(12.9, 18.4)	(0, 0)	(0, 0)	(0, 0)	(0, 0)
15	Multiple myeloma	45	38.3	36.7	19.5	0.01	0.01	0.01	0
		(18.6, 85.7)	(24.9, 56.2)	(22.2, 55.9)	(16, 23.5)	(0, 0)	(0, 0)	(0, 0)	(0, 0)
16	Urinary diseases and male infertility	13.9	14.4	17.7	19.3	0.21	0.21	0.26	0.22
		(10.7, 18)	(12.7, 16.1)	(15, 20.2)	(18.8, 19.8)	(0.2, 0.3)	(0.2, 0.2)	(0.2, 0.3)	(0.2, 0.2)
17	Attention-deficit/hyperactivity disorder	3.5	6.1	7.2	26.3	0.02	0.04	0.05	0.16
		(2.5, 5.4)	(5.3, 7.2)	(6.1, 8.4)	(24.7, 27.8)	(0, 0)	(0, 0)	(0, 0.1)	(0.2, 0.2)
18	Breast cancer	16.4	24.8	23.8	20.7	0.02	0.04	0.04	0.04
		(9, 29.3)	(16.5, 35.2)	(14.8, 37.4)	(19.3, 22.8)	(0, 0)	(0, 0.1)	(0, 0.1)	(0, 0)
19	Ischemic heart disease	14.2	13.4	13.8	21	0.29	0.3	0.32	0.32
		(10.1, 19.4)	(11.4, 15.8)	(11.6, 17.2)	(20.2, 22.2)	(0.2, 0.4)	(0.3, 0.3)	(0.3, 0.4)	(0.3, 0.3)
20	Depressive disorders	4.5	7.2	9.8	19.9	0.11	0.17	0.25	0.38
		(3.4, 6.4)	(6.3, 8)	(8.1, 11.1)	(18.8, 21.5)	(0.1, 0.2)	(0.1, 0.2)	(0.2, 0.3)	(0.4, 0.4)
21	Prostate cancer	31.7	37.8	46.1	14	0.02	0.02	0.02	0.01
		(15.8, 51.4)	(27.8, 54.7)	(28.7, 68.9)	(11.8, 15.9)	(0, 0)	(0, 0)	(0, 0)	(0, 0)
22	Diabetes mellitus type 1	26.7	16.1	18.1	12.4	0.05	0.03	0.03	0.03
		(14.4, 45.6)	(10.2, 26)	(12.2, 25.1)	(10, 15)	(0, 0.1)	(0, 0)	(0, 0)	(0, 0)
23	Other chronic respiratory diseases	11.1	10.7	11.3	16.8	0.16	0.17	0.2	0.23
		(8.3, 14.4)	(9.3, 12.6)	(9.5, 13.4)	(15.9, 17.5)	(0.1, 0.2)	(0.1, 0.2)	(0.2, 0.2)	(0.2, 0.2)
24	Other neurological disorders	7.6	12	9.1	16	0.09	0.16	0.12	0.19
		(5.2, 10.5)	(10.3, 13.9)	(6.9, 12.1)	(15.4, 16.5)	(0.1, 0.1)	(0.1, 0.2)	(0.1, 0.1)	(0.2, 0.2)
25	Other neoplasms	16.2	17.9	16.6	12.6	0.03	0.04	0.04	0.04
		(9.9, 22.5)	(12.8, 24.2)	(11.7, 21.1)	(11.4, 13.5)	(0, 0)	(0, 0)	(0, 0)	(0, 0)
26	Other sense organ diseases	15.5	12.9	13.6	12.5	0.1	0.09	0.09	0.07
		(11.4, 21)	(10.2, 15.8)	(9.9, 17.1)	(11.7, 13.2)	(0.1, 0.1)	(0.1, 0.1)	(0.1, 0.1)	(0.1, 0.1)
27	Upper digestive system diseases	7.1	9.3	11.7	12.9	0.19	0.24	0.28	0.27
		(5.5, 8.7)	(8.2, 10.5)	(10.1, 14.1)	(12.1, 13.8)	(0.2, 0.2)	(0.2, 0.3)	(0.2, 0.3)	(0.3, 0.3)
28	Anxiety disorders	3.4	5.3	5.4	15.5	0.09	0.17	0.22	0.48

© 2025 Sahu M et al. JAMA Health Forum.

		(2.7, 4.4)	(4.8, 6.2)	(4.8, 6.5)	(14.7, 16.7)	(0.1, 0.1)	(0.2, 0.2)	(0.2, 0.2)	(0.5, 0.5)
29	Tracheal, bronchus, and lung	42.1	13.4	32.1	9.7	0.01	0.01	0.01	0
	cancer	(24.2, 70.4)	(8.1, 22.8)	(17.6, 53.8)	(7.4, 12.2)	(0, 0)	(0, 0)	(0, 0)	(0, 0)
30	Other digestive diseases	8.1	7.8	13.1	10.6	0.06	0.05	0.08	0.07
		(5.3, 12.3)	(6.1, 14.2)	(9.8, 17.3)	(9.7, 11.5)	(0, 0.1)	(0, 0.1)	(0.1, 0.1)	(0.1, 0.1)
31	Other infectious diseases	8.1	7.5	6	10.5	0.08	0.1	0.09	0.13
		(5.9, 11.7)	(5.9, 9.9)	(5.2, 7.6)	(10, 11.1)	(0.1, 0.1)	(0.1, 0.1)	(0.1, 0.1)	(0.1, 0.1)
32	Bipolar disorder	3.7	9.3	5	12.7	0.02	0.06	0.04	0.09
		(2.5, 6.5)	(8.1, 11.4)	(4.3, 5.7)	(11.9, 13.6)	(0, 0)	(0.1, 0.1)	(0, 0)	(0.1, 0.1)
33	Gynecological diseases	4.3	5.7	4.9	13.2	0.07	0.1	0.09	0.16
		(3.4, 5.5)	(4.6, 7.1)	(3.9, 6.5)	(12.4, 13.9)	(0.1, 0.1)	(0.1, 0.1)	(0.1, 0.1)	(0.2, 0.2)
34	Acute hepatitis C	12.1	14.6	9.9	7.3	0	0	0	0
		(6.5, 19.3)	(10.5, 20.8)	(6, 13.9)	(6.4, 8.5)	(0, 0)	(0, 0)	(0, 0)	(0, 0)
35	Idiopathic epilepsy	4.9	11.4	6.4	8.3	0.02	0.05	0.03	0.05
		(2.8, 8.3)	(9.5, 13.3)	(4.9, 7.8)	(7.6, 8.8)	(0, 0)	(0, 0.1)	(0, 0)	(0, 0)
36	Other mental disorders	2.7	4.7	5.4	9.7	0.06	0.08	0.14	0.15
		(1.6, 4.8)	(3.6, 6.2)	(4.1, 6.5)	(9, 10.2)	(0, 0.1)	(0.1, 0.1)	(0.1, 0.2)	(0.1, 0.2)
37	Other cardiovascular and	5	7.5	3.7	8.2	0.05	0.06	0.04	0.05
	circulatory diseases	(2, 9.1)	(5.1, 10.4)	(2, 7.6)	(7.6, 8.7)	(0, 0.1)	(0, 0.1)	(0, 0.1)	(0, 0.1)
38	Family planning	2.5	3.4	3.5	10.2	0.04	0.04	0.05	0.15
		(1.8, 3.9)	(2.6, 4.4)	(2.7, 4.4)	(9.2, 11.3)	(0, 0)	(0, 0.1)	(0, 0.1)	(0.1, 0.2)
39	Treatment of obesity	5.7	5.8	4	7.9	0.05	0.04	0.03	0.05
		(3.5, 8.6)	(4.5, 7.1)	(2.6, 5.8)	(7.4, 8.3)	(0, 0.1)	(0, 0.1)	(0, 0)	(0, 0)
40	Non-Hodgkin lymphoma	6.7	10.9	8.8	5.5	0	0	0	0
		(4.3, 10.3)	(8.5, 13)	(5.9, 12.5)	(4.9, 6.1)	(0, 0)	(0, 0)	(0, 0)	(0, 0)
41	Kidney cancer	7.5	5.8	10.1	5.8	0	0	0	0
		(3.9, 14.2)	(3.9, 8.4)	(7.3, 13.7)	(5.2, 6.3)	(0, 0)	(0, 0)	(0, 0)	(0, 0)
42	Blindness and vision loss	6.4	11	5.5	5.7	0.07	0.14	0.07	0.06
		(5.1, 8.3)	(9.1, 12.8)	(4.6, 6.4)	(5.4, 5.9)	(0.1, 0.1)	(0.1, 0.2)	(0.1, 0.1)	(0.1, 0.1)
43	Schizophrenia	3.7	12.8	4.4	6.6	0.01	0.03	0.01	0.02
		(3.2, 4.3)	(11.7, 14)	(4, 4.8)	(6.1, 7)	(0, 0)	(0, 0)	(0, 0)	(0, 0)
44	Headache disorders	1.4	2.7	3.4	8.6	0.04	0.07	0.07	0.14
		(0.9, 2.3)	(2.3, 3.1)	(2.6, 4.1)	(8.2, 9.1)	(0, 0)	(0.1, 0.1)	(0.1, 0.1)	(0.1, 0.1)
45	Lower respiratory infections	2.6	4.1	5.3	6.2	0.07	0.07	0.11	0.11

© 2025 Sahu M et al. JAMA Health Forum.

		(2.1, 3.4)	(3.5, 4.8)	(4.7, 6.1)	(5.9, 6.4)	(0.1, 0.1)	(0.1, 0.1)	(0.1, 0.1)	(0.1, 0.1)
46	Opioid use disorders	2.5	5.4	4.4	7.5	0.02	0.03	0.02	0.04
		(1.3, 4.1)	(4.3, 7.2)	(3.2, 5.5)	(6.9, 8)	(0, 0)	(0, 0)	(0, 0)	(0, 0)
47	Upper respiratory infections	2.7	3.4	3.7	5.7	0.12	0.13	0.16	0.2
		(2, 3.4)	(2.9, 3.9)	(3.1, 4.4)	(5.4, 6)	(0.1, 0.1)	(0.1, 0.1)	(0.1, 0.2)	(0.2, 0.2)
48	Heart Failure	4.3	5.9	5.2	4.4	0.07	0.12	0.1	0.08
		(2.1, 8)	(3.8, 9)	(3, 8.9)	(3.9, 4.8)	(0, 0.1)	(0.1, 0.1)	(0.1, 0.1)	(0.1, 0.1)
49	Low back pain	3.3	4.1	3.1	5.3	0.03	0.05	0.04	0.05
		(1.8, 5)	(3.4, 5)	(2.3, 4.2)	(4.8, 5.7)	(0, 0)	(0, 0.1)	(0, 0.1)	(0.1, 0.1)
50	Osteoarthritis	4.4	8.4	5.3	7.4	0.09	0.24	0.13	0.14
		(2.9, 6.2)	(6.9, 9.9)	(4.1, 6.5)	(6.7, 8.3)	(0.1, 0.1)	(0.2, 0.3)	(0.1, 0.2)	(0.1, 0.1)
51	Interstitial lung disease and pulmonary sarcoidosis	4.8	4.8	6.9	3.8	0	0	0	0
		(1.6, 11.3)	(2.6, 7.1)	(3.1, 12.2)	(3, 4.4)	(0, 0)	(0, 0)	(0, 0)	(0, 0)
52	Stroke	5.4	4.5	4.8	4	0.04	0.05	0.05	0.03
		(2.4, 10.2)	(3.4, 5.8)	(3, 7.2)	(3.7, 4.4)	(0, 0.1)	(0, 0.1)	(0, 0.1)	(0, 0)
53	Nutritional deficiencies	3.9	5.8	4.2	3.7	0.05	0.06	0.05	0.04
		(3.2, 4.9)	(4.6, 6.8)	(3.3, 5.1)	(3.4, 3.9)	(0, 0.1)	(0, 0.1)	(0, 0.1)	(0, 0)
54	Hemoglobinopathies and hemolytic anemias	3.1	5.4	3.3	3	0.01	0.01	0.01	0.01
		(2.1, 4.8)	(4.6, 6.3)	(2.3, 4.7)	(2.8, 3.3)	(0, 0)	(0, 0)	(0, 0)	(0, 0)
55	Pancreatitis	7.6	3.1	4.1	3	0.01	0	0	0
		(4.8, 13.4)	(1.9, 5.7)	(2.4, 6.9)	(2.4, 3.5)	(0, 0)	(0, 0)	(0, 0)	(0, 0)
56	Chronic kidney disease	4.4	6.2	5.1	2	0.04	0.05	0.04	0.02
		(2.8, 6.8)	(5, 7.6)	(3.9, 6.7)	(1.9, 2.3)	(0, 0.1)	(0, 0.1)	(0, 0.1)	(0, 0)
57	Colon and rectum cancer	6	4.9	3.8	3.2	0.01	0.01	0.01	0.01
		(4.1, 8.7)	(3.7, 6.3)	(2.7, 5.4)	(2.9, 3.6)	(0, 0)	(0, 0)	(0, 0)	(0, 0)
58	Gout	7.9	5.7	1.8	3.1	0.07	0.06	0.02	0.04
		(4.9, 11.6)	(3.9, 7.9)	(1.3, 3)	(2.7, 3.5)	(0.1, 0.1)	(0, 0.1)	(0, 0)	(0, 0)
59	Congenital birth defects	2.2	2.1	2.1	3.1	0.01	0.01	0.02	0.01
		(1.4, 4)	(1.6, 2.7)	(1.7, 2.6)	(2.8, 3.4)	(0, 0)	(0, 0)	(0, 0)	(0, 0)
60	Alzheimer's disease and other dementias	7.8	6	14	4.7	0.08	0.12	0.18	0.08
		(3.3, 16.5)	(4.2, 8.5)	(7.3, 21.6)	(3.7, 5.9)	(0, 0.1)	(0.1, 0.2)	(0.1, 0.3)	(0.1, 0.1)
61	Tobacco intervention	1.6	2.6	2.2	3.1	0.01	0.01	0.01	0.02
		(1, 2.2)	(2.1, 3.1)	(1.6, 2.8)	(2.9, 3.3)	(0, 0)	(0, 0)	(0, 0)	(0, 0)
62		4	2.8	4.4	2.3	0.03	0.03	0.03	0.02

© 2025 Sahu M et al. JAMA Health Forum.

	Cirrhosis and other chronic liver diseases	(2.5, 6.6)	(2, 3.4)	(3.4, 6.3)	(2.1, 2.5)	(0, 0)	(0, 0)	(0, 0)	(0, 0)
63	Liver cancer	7.1	2.8	5	2	0	0	0	0
		(4, 10.5)	(1.9, 3.7)	(3.8, 6.6)	(1.8, 2.2)	(0, 0)	(0, 0)	(0, 0)	(0, 0)
64	Non-rheumatic valvular heart disease	5.6	4.2	3.9	2.7	0.04	0.04	0.03	0.02
		(2.5, 11.1)	(2.7, 6.7)	(2, 6.7)	(2.2, 3.2)	(0, 0.1)	(0, 0.1)	(0, 0.1)	(0, 0)
65	Ovarian cancer	5.1	3.3	4.9	2.4	0	0	0	0
		(1.9, 11.4)	(1.6, 6.3)	(2.5, 8.9)	(1.6, 3)	(0, 0)	(0, 0)	(0, 0)	(0, 0)
66	Acute renal failure	2.4	4.4	3.5	1.7	0.01	0.02	0.01	0.01
		(1.9, 2.9)	(3.7, 5.1)	(2.8, 4.1)	(1.5, 1.8)	(0, 0)	(0, 0)	(0, 0)	(0, 0)
67	Parkinson's disease	3.4	1.1	1.7	3.2	0.02	0.01	0.02	0.02
		(1.6, 6.6)	(0.6, 1.6)	(1, 2.6)	(3, 3.4)	(0, 0)	(0, 0)	(0, 0)	(0, 0)
68	Otitis media	1.4	1.3	1.7	2.5	0.05	0.04	0.06	0.08
		(0.9, 2)	(1.1, 1.6)	(1.4, 2)	(2.3, 2.6)	(0, 0.1)	(0, 0)	(0.1, 0.1)	(0.1, 0.1)
69	Other unintentional injuries	0.9	1.4	1.5	2.3	0.01	0.01	0.02	0.02
		(0.7, 1.2)	(1.2, 1.5)	(1.3, 1.6)	(2.2, 2.4)	(0, 0)	(0, 0)	(0, 0)	(0, 0)
70	Autism spectrum disorders	1.4	1.3	1.1	2.5	0.01	0.01	0.01	0.01
		(0.3, 4.1)	(0.9, 2)	(0.7, 1.9)	(2, 2.9)	(0, 0)	(0, 0)	(0, 0)	(0, 0)
71	Neck pain	1.2	1.5	1.6	2.4	0.01	0.01	0.01	0.01
		(0.7, 1.7)	(1.1, 1.8)	(1.2, 2.3)	(2.2, 2.6)	(0, 0)	(0, 0)	(0, 0)	(0, 0)
72	Lower extremity peripheral arterial disease	4.1	5.6	4.8	3.3	0.06	0.09	0.07	0.04
		(2.4, 6.9)	(3.5, 8.3)	(2.7, 6.7)	(2.8, 3.8)	(0, 0.1)	(0.1, 0.1)	(0, 0.1)	(0, 0)
73	Malignant neoplasm of bone and articular cartilage	3.6	1.4	1.6	1.3	0.01	0	0	0
		(2.3, 5.6)	(0.8, 2)	(0.9, 2.3)	(1.1, 1.4)	(0, 0)	(0, 0)	(0, 0)	(0, 0)
74	Cardiomyopathy and myocarditis	1.5	1.9	1.7	1.2	0.02	0.02	0.02	0.01
		(0.5, 4.2)	(1.2, 2.9)	(0.9, 2.9)	(1, 1.4)	(0, 0)	(0, 0)	(0, 0)	(0, 0)
75	Non-melanoma skin cancer	1.9	0.8	1.1	2	0.01	0.01	0.01	0.01
		(0.8, 4.4)	(0.5, 1.4)	(0.6, 2.1)	(1.8, 2.1)	(0, 0)	(0, 0)	(0, 0)	(0, 0)
76	Malignant skin melanoma	0.9	1	0.9	1.8	0	0	0	0
		(0.5, 1.4)	(0.6, 1.5)	(0.6, 1.3)	(1.6, 2)	(0, 0)	(0, 0)	(0, 0)	(0, 0)
77	Gallbladder and biliary diseases	1.2	0.8	1.5	1.2	0	0	0.01	0
		(0.7, 2)	(0.5, 1.2)	(0.7, 2.2)	(1.1, 1.3)	(0, 0)	(0, 0)	(0, 0)	(0, 0)
78	Pregnancy and postpartum care	1.2	2.1	1.5	2.1	0.01	0.01	0.01	0.01
		(0.8, 2.1)	(1.5, 2.7)	(1.1, 2.4)	(1.8, 2.5)	(0, 0)	(0, 0)	(0, 0)	(0, 0)

© 2025 Sahu M et al. JAMA Health Forum.

79	Enteric infections	0.9	1	1	1.1	0.01	0.01	0.01	0.01
		(0.5, 1.4)	(0.7, 1.3)	(0.8, 1.3)	(1, 1.2)	(0, 0)	(0, 0)	(0, 0)	(0, 0)
80	Hypertensive heart disease	1.9	1.2	1.6	1	0.02	0.02	0.02	0.01
		(1, 3.6)	(0.7, 1.9)	(1, 2.6)	(0.9, 1.1)	(0, 0)	(0, 0)	(0, 0)	(0, 0)
81	Brain and central nervous system cancer	0.4	0.5	0.4	0.9	0	0	0	0
		(0.2, 1)	(0.3, 0.7)	(0.3, 0.6)	(0.9, 1)	(0, 0)	(0, 0)	(0, 0)	(0, 0)
82	Thyroid cancer	0.7	0.6	0.8	0.8	0.01	0.01	0.01	0.01
		(0.3, 1.5)	(0.4, 0.9)	(0.4, 1.1)	(0.7, 0.9)	(0, 0)	(0, 0)	(0, 0)	(0, 0)
83	Sexually transmitted infections excluding HIV	0.8	1.1	0.7	0.5	0.01	0.01	0.01	0.01
		(0.3, 1.3)	(0.7, 1.5)	(0.4, 1)	(0.4, 0.7)	(0, 0)	(0, 0)	(0, 0)	(0, 0)
84	Inguinal, femoral, and abdominal hernia	1.1	0.8	1	0.5	0.01	0.01	0.01	0.01
		(0.4, 2)	(0.3, 1.4)	(0.4, 1.5)	(0.4, 0.6)	(0, 0)	(0, 0)	(0, 0)	(0, 0)
85	Oral disorders	0.6	0.7	0.6	0.6	0.03	0.03	0.03	0.02
		(0.5, 0.8)	(0.6, 0.9)	(0.5, 0.8)	(0.5, 0.6)	(0, 0)	(0, 0)	(0, 0)	(0, 0)
86	Alcohol use disorders	0.2	0.5	0.3	0.7	0.01	0.01	0.01	0.01
		(0.1, 0.3)	(0.4, 0.6)	(0.3, 0.4)	(0.7, 0.7)	(0, 0)	(0, 0)	(0, 0)	(0, 0)
87	Pulmonary Arterial Hypertension	1.1	0.7	0.9	0.5	0.01	0.01	0.01	0
		(0.5, 2.3)	(0.4, 1.1)	(0.4, 1.6)	(0.4, 0.6)	(0, 0)	(0, 0)	(0, 0)	(0, 0)
88	Soft tissue and other extraosseous sarcomas	0.2	0.3	0.3	0.6	0	0	0	0
		(0.1, 0.3)	(0.2, 0.4)	(0.2, 0.4)	(0.6, 0.7)	(0, 0)	(0, 0)	(0, 0)	(0, 0)
89	Other malignant neoplasms	0.2	0.3	0.3	0.6	0	0	0	0
		(0.1, 0.2)	(0.2, 0.3)	(0.2, 0.3)	(0.6, 0.7)	(0, 0)	(0, 0)	(0, 0)	(0, 0)
90	Aortic aneurysm	1.6	0.8	1	0.4	0.01	0.01	0.01	0
		(0.6, 2.9)	(0.3, 1.6)	(0.6, 1.5)	(0.4, 0.6)	(0, 0)	(0, 0)	(0, 0)	(0, 0)
91	Pancreatic cancer	0.6	0.5	0.9	0.4	0	0	0	0
		(0.3, 1)	(0.3, 0.9)	(0.5, 1.5)	(0.4, 0.6)	(0, 0)	(0, 0)	(0, 0)	(0, 0)
92	Other direct maternal disorders	0.4	0.8	0.4	0.8	0	0	0	0
		(0.4, 0.5)	(0.7, 1)	(0.3, 0.4)	(0.7, 0.9)	(0, 0)	(0, 0)	(0, 0)	(0, 0)
93	Neglected tropical diseases and malaria	0.2	0.2	0.3	0.4	0	0	0	0
		(0.2, 0.5)	(0.2, 0.4)	(0.2, 0.5)	(0.4, 0.5)	(0, 0)	(0, 0)	(0, 0)	(0, 0)
94	Rheumatic heart disease	0.6	0.6	0.5	0.3	0.01	0.01	0.01	0
		(0.3, 1.1)	(0.3, 0.8)	(0.3, 0.7)	(0.3, 0.3)	(0, 0)	(0, 0)	(0, 0)	(0, 0)
95	Other substance use disorders	0.1	0.3	0.2	0.4	0	0	0	0
		(0.1, 0.3)	(0.2, 0.4)	(0.1, 0.4)	(0.3, 0.4)	(0, 0)	(0, 0)	(0, 0)	(0, 0)

© 2025 Sahu M et al. JAMA Health Forum.

96	Septicemia	0.3	0.3	0.3	0.3	0	0	0	0
		(0.1, 0.6)	(0.2, 0.5)	(0.2, 0.4)	(0.3, 0.3)	(0, 0)	(0, 0)	(0, 0)	(0, 0)
97	Eating disorders	0.5	0.5	0.2	0.6	0	0	0	0
		(0.2, 1.1)	(0.2, 0.8)	(0.1, 0.4)	(0.5, 0.7)	(0, 0)	(0, 0)	(0, 0)	(0, 0)
98	Age-related and other hearing loss	0.4	0.2	0.3	0.2	0	0	0	0
		(0.2, 0.7)	(0.2, 0.4)	(0.2, 0.5)	(0.2, 0.3)	(0, 0)	(0, 0)	(0, 0)	(0, 0)
99	Uterine cancer	0.3	0.4	0.3	0.3	0	0	0	0
		(0.1, 0.6)	(0.2, 0.6)	(0.2, 0.5)	(0.2, 0.4)	(0, 0)	(0, 0)	(0, 0)	(0, 0)
100	Falls	0.1	0.2	0.2	0.2	0	0	0	0
		(0.1, 0.2)	(0.1, 0.3)	(0.1, 0.3)	(0.2, 0.2)	(0, 0)	(0, 0)	(0, 0)	(0, 0)
101	Idiopathic developmental intellectual disability	0.1	0.1	0.1	0.2	0	0	0	0
		(0, 0.3)	(0.1, 0.1)	(0.1, 0.1)	(0.2, 0.3)	(0, 0)	(0, 0)	(0, 0)	(0, 0)
102	Cervical cancer	0.1	0.2	0.2	0.2	0	0	0	0
		(0.1, 0.2)	(0.1, 0.3)	(0.1, 0.3)	(0.2, 0.3)	(0, 0)	(0, 0)	(0, 0)	(0, 0)
103	Motor neuron disease	0.1	0.1	0.1	0.2	0	0	0	0
		(0.1, 0.2)	(0.1, 0.1)	(0.1, 0.1)	(0.2, 0.2)	(0, 0)	(0, 0)	(0, 0)	(0, 0)
104	Bladder cancer	0.4	0.2	0.2	0.2	0	0	0	0
		(0.2, 0.6)	(0.1, 0.5)	(0.1, 0.4)	(0.2, 0.2)	(0, 0)	(0, 0)	(0, 0)	(0, 0)
105	Paralytic ileus and intestinal obstruction	0.1	0.1	0.1	0.1	0	0	0	0
		(0.1, 0.2)	(0.1, 0.2)	(0.1, 0.2)	(0.1, 0.1)	(0, 0)	(0, 0)	(0, 0)	(0, 0)
106	Tuberculosis	0.1	0.1	0.1	0.1	0	0	0	0
		(0.1, 0.1)	(0.1, 0.1)	(0.1, 0.1)	(0.1, 0.1)	(0, 0)	(0, 0)	(0, 0)	(0, 0)
107	Stomach cancer	0.1	0.1	0	0.1	0	0	0	0
		(0, 0.1)	(0, 0.1)	(0, 0.1)	(0.1, 0.1)	(0, 0)	(0, 0)	(0, 0)	(0, 0)
108	Endocarditis	0	0	0.1	0.1	0	0	0	0
		(0, 0.1)	(0, 0.1)	(0, 0.1)	(0.1, 0.1)	(0, 0)	(0, 0)	(0, 0)	(0, 0)
109	Esophageal cancer	0.1	0.1	0.1	0.1	0	0	0	0
		(0, 0.1)	(0.1, 0.1)	(0, 0.1)	(0.1, 0.1)	(0, 0)	(0, 0)	(0, 0)	(0, 0)
110	Lip and oral cavity cancer	0.1	0.1	0	0.1	0	0	0	0
		(0, 0.1)	(0, 0.1)	(0, 0.1)	(0.1, 0.1)	(0, 0)	(0, 0)	(0, 0)	(0, 0)
111	Indirect maternal deaths	0.1	0.1	0.1	0.1	0	0	0	0
		(0, 0.1)	(0.1, 0.1)	(0, 0.1)	(0, 0.1)	(0, 0)	(0, 0)	(0, 0)	(0, 0)
112	Hodgkin lymphoma	0	0	0	0	0	0	0	0
		(0, 0)	(0, 0)	(0, 0)	(0, 0.1)	(0, 0)	(0, 0)	(0, 0)	(0, 0)

© 2025 Sahu M et al. JAMA Health Forum.

113	Other neonatal disorders	0	0	0	0	0	0	0	0
		(0, 0)	(0, 0)	(0, 0)	(0, 0)	(0, 0)	(0, 0)	(0, 0)	(0, 0)
114	Other pharynx cancer	0	0	0	0	0	0	0	0
		(0, 0.1)	(0, 0)	(0, 0)	(0, 0)	(0, 0)	(0, 0)	(0, 0)	(0, 0)
115	Maternal abortion and miscarriage	0.1	0	0	0	0	0	0	0
		(0, 0.1)	(0, 0.1)	(0, 0.1)	(0, 0.1)	(0, 0)	(0, 0)	(0, 0)	(0, 0)
116	Transport injuries	0	0	0	0	0	0	0	0
		(0, 0)	(0, 0)	(0, 0)	(0, 0)	(0, 0)	(0, 0)	(0, 0)	(0, 0)
117	Exposure to mechanical forces	0	0	0	0	0	0	0	0
		(0, 0)	(0, 0)	(0, 0)	(0, 0)	(0, 0)	(0, 0)	(0, 0)	(0, 0)
118	Vascular intestinal disorders	0	0	0	0	0	0	0	0
		(0, 0)	(0, 0)	(0, 0)	(0, 0)	(0, 0)	(0, 0)	(0, 0)	(0, 0)
119	Larynx cancer	0	0	0	0	0	0	0	0
		(0, 0)	(0, 0)	(0, 0)	(0, 0)	(0, 0)	(0, 0)	(0, 0)	(0, 0)
120	Gallbladder and biliary tract cancer	0	0	0	0	0	0	0	0
		(0, 0)	(0, 0)	(0, 0)	(0, 0)	(0, 0)	(0, 0)	(0, 0)	(0, 0)
121	Testicular cancer	0	0	0	0	0	0	0	0
		(0, 0)	(0, 0)	(0, 0)	(0, 0)	(0, 0)	(0, 0)	(0, 0)	(0, 0)
122	Neuroblastoma and other peripheral nervous cell tumors	0	0	0	0	0	0	0	0
		(0, 0)	(0, 0)	(0, 0)	(0, 0)	(0, 0)	(0, 0)	(0, 0)	(0, 0)
123	Maternal hemorrhage	0	0	0	0	0	0	0	0
		(0, 0)	(0, 0)	(0, 0)	(0, 0)	(0, 0)	(0, 0)	(0, 0)	(0, 0)
124	Meningitis	0	0	0	0	0	0	0	0
		(0, 0)	(0, 0)	(0, 0)	(0, 0)	(0, 0)	(0, 0)	(0, 0)	(0, 0)
125	Neonatal encephalopathy due to birth asphyxia and trauma	0	0	0	0	0	0	0	0
		(0, 0)	(0, 0)	(0, 0)	(0, 0)	(0, 0)	(0, 0)	(0, 0)	(0, 0)
126	Appendicitis	0	0	0	0	0	0	0	0
		(0, 0)	(0, 0)	(0, 0)	(0, 0)	(0, 0)	(0, 0)	(0, 0)	(0, 0)
127	Maternal hypertensive disorders	0	0	0	0	0	0	0	0
		(0, 0)	(0, 0)	(0, 0)	(0, 0)	(0, 0)	(0, 0)	(0, 0)	(0, 0)
128	Mesothelioma	0	0	0	0	0	0	0	0
		(0, 0)	(0, 0)	(0, 0)	(0, 0)	(0, 0)	(0, 0)	(0, 0)	(0, 0)
129	Maternal sepsis and other maternal infections	0	0	0	0	0	0	0	0
		(0, 0)	(0, 0)	(0, 0)	(0, 0)	(0, 0)	(0, 0)	(0, 0)	(0, 0)

© 2025 Sahu M et al. JAMA Health Forum.

130	Neonatal preterm birth	0.5	0.5	0.6	0.5	0.01	0.01	0.01	0.01
		(0.3, 1)	(0.3, 0.7)	(0.4, 0.7)	(0.4, 0.7)	(0, 0)	(0, 0)	(0, 0)	(0, 0)
131	Pneumoconiosis	0	0	0	0	0	0	0	0
		(0, 0)	(0, 0)	(0, 0)	(0, 0)	(0, 0)	(0, 0)	(0, 0)	(0, 0)
132	Interpersonal violence	0	0	0	0	0	0	0	0
		(0, 0)	(0, 0)	(0, 0)	(0, 0)	(0, 0)	(0, 0)	(0, 0)	(0, 0)
133	Eye cancer	0	0	0	0	0	0	0	0
		(0, 0)	(0, 0)	(0, 0)	(0, 0)	(0, 0)	(0, 0)	(0, 0)	(0, 0)
134	Nasopharynx cancer	0	0	0	0	0	0	0	0
		(0, 0)	(0, 0)	(0, 0)	(0, 0)	(0, 0)	(0, 0)	(0, 0)	(0, 0)
135	Maternal obstructed labor and uterine rupture	0	0	0	0	0	0	0	0
		(0, 0)	(0, 0)	(0, 0)	(0, 0)	(0, 0)	(0, 0)	(0, 0)	(0, 0)
136	Self-harm	0	0	0	0	0	0	0	0
		(0, 0)	(0, 0)	(0, 0)	(0, 0)	(0, 0)	(0, 0)	(0, 0)	(0, 0)
137	Late maternal deaths	0	0	0	0	0	0	0	0
		(0, 0)	(0, 0)	(0, 0)	(0, 0)	(0, 0)	(0, 0)	(0, 0)	(0, 0)
138	Donor	0	0	0	0	0	0	0	0
		(0, 0)	(0, 0)	(0, 0)	(0, 0)	(0, 0)	(0, 0)	(0, 0)	(0, 0)
139	Hemolytic disease and other neonatal jaundice	0	0	0	0	0	0	0	0
		(0, 0)	(0, 0)	(0, 0)	(0, 0)	(0, 0)	(0, 0)	(0, 0)	(0, 0)
140	Ectopic pregnancy	0	0	0	0	0	0	0	0
		(0, 0)	(0, 0)	(0, 0)	(0, 0)	(0, 0)	(0, 0)	(0, 0)	(0, 0)
141	Neonatal sepsis and other neonatal infections	0	0	0	0	0	0	0	0
		(0, 0)	(0, 0)	(0, 0)	(0, 0)	(0, 0)	(0, 0)	(0, 0)	(0, 0)
142	Acute glomerulonephritis	0	0	0	0	0	0	0	0
		(0, 0)	(0, 0)	(0, 0)	(0, 0)	(0, 0)	(0, 0)	(0, 0)	(0, 0)
143	Maternal deaths aggravated by HIV/AIDS	0	0	0	0	0	0	0	0
		(0, 0)	(0, 0)	(0, 0)	(0, 0)	(0, 0)	(0, 0)	(0, 0)	(0, 0)

© 2025 Sahu M et al. JAMA Health Forum.

B. Table S3.2: *Pharmaceutical spending and utilization per prevalent case by race and ethnicity (age-standardized), for 52 health conditions*

Here we present spending and utilization per prevalent case by race/ethnicity, for each of 52 health conditions with available prevalence/incidence estimates. Health conditions are ranked by total retail pharmaceutical spending. All groups other than Hispanic are non-Hispanic ethnicity. The mean values across 100 draws are presented in the top row, with the 95% Uncertainty Interval below.

Rank (Total drug spending)	Health Condition	Spending per prevalent case (US$)				Prescription fills per prevalent case (n)			
		Asian and Pacific Islander	Black	Hispanic	White	Asian and Pacific Islander	Black	Hispanic	White
1	Diabetes mellitus type 2	1005	553.7	918.8	1187.7	7.2	3.3	5.2	4.6
		(904.3, 1126.6)	(507.5, 612.7)	(846.7, 983)	(1120.6, 1241.9)	(6.6, 7.6)	(3, 3.6)	(4.8, 5.6)	(4.5, 4.8)
2	HIV/AIDS	18256.4	6078.6	13165.4	10558.5	9.5	4.7	7	9.4
		(6371.1, 51082.5)	(4726.3, 8726.6)	(7827.9, 20827.4)	(4850.7, 15692.7)	(5.3, 16.1)	(3.9, 5.4)	(5.8, 8.5)	(8.5, 11)
3	Multiple sclerosis	57755.3	13719.8	27099.3	26049.9	13.5	2.8	6	4.9
		(23107.1, 125175.9)	(9023.9, 21152.8)	(12376.4, 53288.3)	(22494.8, 29521)	(6.6, 25.9)	(2, 3.9)	(3.9, 10.5)	(4.5, 5.5)
4	Atrial fibrillation and flutter	1795	860.5	1708.9	1724.8	8.6	4.1	9.3	6.6
		(1356, 2303)	(763.3, 969.7)	(1353.3, 2091.1)	(1569.7, 1893)	(6.9, 10.7)	(3.5, 4.8)	(8.1, 11.2)	(6.3, 7)
5	Rheumatoid arthritis	2678.6	3301.6	3982.2	7379.2	6	9.1	7.1	15.8
		(1083.4, 6859.9)	(2109.9, 5338.9)	(2802.5, 5209.7)	(6506.7, 8324.8)	(3.2, 14)	(6.3, 12.9)	(6, 8.4)	(14, 17.1)
6	Chronic obstructive pulmonary disease	802.7	220.6	319.6	346.8	3.6	1.6	2.3	1.7
		(438, 1302)	(185.4, 261.9)	(261.3, 374.5)	(319.3, 371.4)	(2.6, 4.8)	(1.4, 1.8)	(1.9, 2.7)	(1.5, 1.7)
7	Leukemia	304139.6	137571.2	198000.6	133171.7	84.9	65.2	46.1	58.4
		(115217.2, 839019.2)	(67585.9, 263655)	(106546.4, 344080.3)	(91141.8, 184144)	(42.7, 143.8)	(40.5, 91.5)	(32.2, 65.8)	(47.5, 69.2)
8	Multiple myeloma	787512.9	246458.8	516394.8	474993.1	436.8	96.8	195.9	199.4

© 2025 Sahu M et al. JAMA Health Forum.

		(342180.9, 1581820.9)	(170763.1, 368745.4)	(329565.3, 800040.4)	(369643.9, 587760.4)	(155.6, 1049.1)	(46.3, 185.7)	(95.5, 312.9)	(133.4, 272.9)
9	Breast cancer	14643.4	10714.7	15854.5	27538.1	46.6	41.1	56.3	109.5
		(8346.9, 21594)	(7962, 15804.7)	(11509.9, 22453.9)	(24666.7, 30396.8)	(30.6, 76.8)	(30.4, 60)	(43.8, 77.8)	(98.6, 120.5)
10	Ischemic heart disease	796.7	571.4	664.7	1610.1	10.5	6	11.3	13.5
		(536, 1555.9)	(361.3, 920.3)	(493.4, 1013.3)	(1321, 1797)	(7.3, 14.4)	(5.4, 6.8)	(9.6, 13.4)	(12.5, 14.3)
11	Prostate cancer	19921.6	4734.3	12639.6	9947.4	217.6	49	78.5	103.5
		(8379, 48392)	(3139.7, 6411.5)	(7158.3, 19534.8)	(6392.9, 14423.4)	(41.3, 593.7)	(13.9, 93.8)	(26, 172.8)	(57.9, 165.4)
12	Tracheal, bronchus, and lung cancer	91102.7	23709.3	54980.9	35865.5	100.9	33.2	44.7	30.3
		(46217.5, 141585.7)	(14623, 35272)	(35935.3, 89790.5)	(28810, 43596)	(35, 201.2)	(17.8, 51)	(23.8, 79.9)	(22.5, 40.8)
13	Non-Hodgkin lymphoma	13958.2	17045.9	16133	11969.8	14.4	9.6	10.4	9.3
		(9891.6, 20353.4)	(12906.1, 20920.7)	(10915, 20973.7)	(10036.6, 13824.8)	(8.3, 23.4)	(6.7, 12.5)	(7.1, 13.1)	(7.5, 10.8)
14	Kidney cancer	31593.9	14544.6	27703.3	18855.4	21	6.7	12	9.9
		(18312.1, 57865.9)	(9960.7, 20311.3)	(21353.4, 34612.4)	(15959.5, 22093.3)	(11.4, 42.9)	(4.8, 8.8)	(9, 16.1)	(8, 12.2)
15	Lower respiratory infections	19624.8	16129.6	41130.1	52582.1	561.3	246.4	695.1	774
		(16433.5, 23851.6)	(13712.7, 18981.3)	(36811.1, 46363.8)	(49354.7, 55547.7)	(470.5, 682.3)	(225.4, 269.6)	(636.6, 764)	(742.1, 800.7)
16	Opioid use disorders	2801.9	643.7	1002.4	725.4	19	3.5	5.3	3.9
		(1173.3, 5211.7)	(443.6, 915.7)	(582.5, 1648.4)	(670.5, 782.3)	(10.2, 29.7)	(2.5, 5.5)	(3.3, 8.7)	(3.6, 4.3)
17	Stroke	140.8	66.3	118.1	159.2	1.2	0.8	1.2	1.3
		(76.3, 232.5)	(53.5, 79.8)	(84.9, 159.3)	(148.7, 168)	(0.6, 1.8)	(0.6, 0.9)	(1, 1.5)	(1.3, 1.4)
18	Nutritional deficiencies	132.8	74.3	99.7	65.6	1.7	0.7	1	0.7
		(102.6, 172.1)	(58.1, 88.9)	(79, 122.6)	(59.6, 69.4)	(1.3, 2.1)	(0.6, 0.8)	(0.9, 1.2)	(0.6, 0.7)
19	Colon and rectum cancer	7044.8	4159.6	5514.9	6286.2	25.9	19.8	22.4	28.7

© 2025 Sahu M et al. JAMA Health Forum.

		(4465.8, 12362.2)	(2577.5, 6073.6)	(3778.9, 7727.9)	(5452.7, 7226.8)	(16, 41.2)	(13.1, 27.3)	(14.5, 29.5)	(25, 31.9)
20	Liver cancer	46935.5	18790.6	22312.9	35627	52.1	44.9	38.7	51.7
		(20960.2, 77281.6)	(11411.5, 31118.9)	(17019.9, 30471.8)	(29594.2, 41928.7)	(23, 85.7)	(27.7, 66.8)	(25.6, 60.6)	(41.9, 62.6)
21	Ovarian cancer	24880.5	11399.1	18587.4	9018.5	11.6	6.4	7.8	5
		(10093.1, 50052.1)	(5189, 21677.1)	(9398.1, 31801.4)	(5320, 11391.1)	(5.8, 20.1)	(4.4, 9.9)	(4.8, 11.1)	(4, 6)
22	Malignant neoplasm of bone and articular cartilage	438166.4	81535.3	118127.6	110163.6	758	163.4	272.5	175.1
		(292980, 644293.4)	(50560.7, 117322.1)	(74250.1, 168777.5)	(98339, 121327.5)	(562.9, 923)	(113.4, 258.8)	(195.7, 347.8)	(159.1, 190.2)
23	Cardiomyopathy and myocarditis	1206.6	522.6	1171.4	798.8	13.7	5.2	13.8	7.3
		(394.7, 3068.5)	(304.7, 782.9)	(688.5, 1794.5)	(638.9, 933.1)	(5.8, 30.5)	(3.6, 7.4)	(8.7, 18.8)	(6.1, 8.6)
24	Malignant skin melanoma	10425.3	7628.8	5035	4508	22.8	15.7	8.8	3.9
		(5165.4, 17278.7)	(4875.8, 12679.1)	(2887.5, 7188.8)	(4000.9, 4988.9)	(10.2, 39.9)	(11.3, 26.3)	(5.3, 13.5)	(3.3, 4.6)
25	Hypertensive heart disease	700.6	96	374.1	276.3	10.3	1.7	4.8	4.2
		(354.6, 1438)	(57.6, 134.8)	(248.3, 562.4)	(236.8, 308.2)	(6, 15.6)	(1.1, 2.3)	(3.4, 6.3)	(3.8, 4.7)
26	Brain and central nervous system cancer	7664.4	5649.9	6393.5	11509.9	27	20.2	20.7	15.5
		(3803.4, 16961.1)	(3666.6, 8915.6)	(4902.1, 9805.2)	(10726.7, 12452.7)	(17.1, 43)	(14, 27.4)	(15.9, 26.2)	(14, 17.7)
27	Thyroid cancer	8621.1	6544.6	6525.4	11865.8	75.2	58.7	53.4	106.1
		(2678.9, 20121.3)	(4185.2, 11560.9)	(4511.8, 8393.9)	(9938.9, 14207.6)	(29.5, 180.9)	(44, 85.8)	(34, 71.9)	(97.2, 115.7)
28	Inguinal, femoral, and abdominal hernia	5992.7	1323	2080.6	1321.9	90.3	24	33.5	26.4
		(2347.7, 12221.3)	(797.1, 2156.2)	(1181.7, 3042.5)	(1130.7, 1534)	(45.1, 169.6)	(16.9, 36)	(22.3, 44.3)	(23, 29.8)
29	Alcohol use disorders	33.1	28.1	30.2	63.9	1.1	0.8	0.9	1.8
		(23.7, 46.3)	(24.2, 32.5)	(25.5, 34.5)	(55.3, 70.2)	(0.8, 1.4)	(0.7, 0.9)	(0.8, 1.1)	(1.7, 1.8)
30	Soft tissue and other extraosseous sarcomas	6963.7	7167.9	7078.5	14911.1	17.1	9.8	13.9	12.5
		(3212.1, 14233.5)	(4776.4, 11581.2)	(4804.7, 10068.3)	(12780.7, 17199.7)	(10.1, 24.5)	(7, 13.9)	(10.5, 18.5)	(10.8, 14.1)

© 2025 Sahu M et al. JAMA Health Forum.

31	Other malignant neoplasms	2861.1 (1532.7, 4989.2)	4752.2 (2461.9, 6830.3)	2376.5 (1666.7, 3460.6)	5784.2 (5134.2, 6381.2)	7.9 (6.8, 9.4)	7.6 (6.6, 9.3)	8 (7.2, 9.1)	9.6 (9.1, 10.1)
32	Pancreatic cancer	7608.6 (2736.4, 18264.8)	2796.9 (1831.9, 5202)	4560.6 (2486.9, 6234.5)	6102.7 (5082.7, 7156.3)	45.3 (21.2, 72.8)	15.6 (10, 27.1)	25.1 (14.8, 37.1)	23 (18.5, 27.9)
33	Uterine cancer	1421.3 (313.8, 3812.9)	450.2 (192.2, 834.4)	454.5 (225.3, 765.3)	1068.7 (622.1, 1459.6)	7.9 (2.2, 22.4)	3.7 (1.6, 6.8)	3.1 (1.8, 4.9)	4.5 (2.5, 6.9)
34	Falls	3.4 (2.3, 5.2)	3.8 (2.6, 5.8)	3.6 (2.6, 4.7)	3.8 (3.5, 4)	0.1 (0.1, 0.1)	0.1 (0.1, 0.1)	0.1 (0.1, 0.1)	0.1 (0.1, 0.1)
35	Cervical cancer	2783.7 (843.8, 7787.1)	990.3 (592.7, 1714.9)	1784.7 (1089, 2799.9)	2138.1 (1645.7, 2536.1)	24.5 (9.5, 56.9)	10.4 (6.4, 17.8)	20.5 (12.9, 32.2)	20.5 (15.3, 24.6)
36	Bladder cancer	1446.7 (708.1, 3083.4)	544.7 (311.6, 905.4)	770.5 (511.6, 1033.3)	586.1 (498, 708.2)	20.2 (11.6, 32.2)	9.8 (6, 13.8)	10.1 (7, 15.8)	7.1 (5.4, 9.1)
37	Tuberculosis	0.7 (0.5, 1)	0.9 (0.7, 1)	1.2 (1, 1.4)	1.6 (1.4, 1.8)	0 (0, 0)	0 (0, 0)	0 (0, 0)	0 (0, 0)
38	Stomach cancer	1210.6 (194.7, 2697.3)	536.6 (303.5, 961.8)	406.7 (196.6, 640.9)	2318.2 (1868, 2686.1)	9.3 (3.5, 19.5)	6.2 (4, 10.8)	5.7 (3.3, 9.7)	14.7 (11.6, 17.1)
39	Endocarditis	692.5 (391.3, 1294.8)	322.1 (199.5, 450.2)	472 (292.8, 729.6)	752.8 (680.6, 813)	15.2 (9, 30.2)	5.8 (4, 8)	14.9 (10.5, 20.5)	13.3 (11.8, 14.9)
40	Esophageal cancer	1963.6 (732.6, 3699.9)	1810.3 (704.6, 3789.3)	1244.8 (770.3, 1874)	1548.1 (1098.9, 1975.5)	25.1 (13, 44.1)	15.6 (9.3, 24.4)	15.6 (11, 21.2)	13.5 (10.7, 16.4)
41	Lip and oral cavity cancer	672.1 (273, 1313.5)	514.6 (272, 1066.7)	489.3 (287.6, 785.8)	715.9 (598.3, 805.9)	8.9 (4.6, 15.7)	8.8 (5.4, 13.6)	8.4 (6.3, 12.1)	6.7 (5.7, 7.7)
42	Hodgkin lymphoma	1643.1 (603.3, 4087.8)	2328.7 (1478.9, 3268.9)	1598.3 (649.9, 2900.4)	4445.2 (3169.2, 8764.1)	33.4 (14.3, 69.7)	25.7 (13.9, 53.5)	19.7 (10.8, 49.3)	21.8 (16.9, 33.1)

© 2025 Sahu M et al. JAMA Health Forum.

43	Other pharynx cancer	4971.7	1250.8	2212.5	1517.7	48	21.1	31.7	20.2
		(766.7, 9916.8)	(516.9, 2620.1)	(617.6, 4899.2)	(997.8, 2311.4)	(14.8, 96)	(9.9, 38.6)	(13.5, 61.8)	(12, 31.5)
44	Transport injuries	2.7	2.6	2.4	2.2	0.1	0.1	0.2	0.1
		(1.6, 4)	(1.8, 3.3)	(1.7, 3.5)	(2, 2.5)	(0.1, 0.2)	(0.1, 0.1)	(0.1, 0.2)	(0.1, 0.1)
45	Exposure to mechanical forces	1.9	1.2	1.3	0.9	0.1	0	0	0
		(1.1, 3.3)	(0.9, 1.6)	(1, 1.7)	(0.8, 1)	(0, 0.1)	(0, 0)	(0, 0.1)	(0, 0)
46	Vascular intestinal disorders	206.4	82.2	115.2	117.3	7.4	2.6	4.3	2.7
		(113.7, 342.6)	(56.7, 110.8)	(76.8, 163)	(104.1, 139.7)	(5.1, 9.4)	(2.1, 3.1)	(3.3, 5.5)	(2.4, 3)
47	Larynx cancer	1153.4	546.6	456.9	473.6	27	11.3	12.5	10.5
		(516.2, 2174.3)	(246.1, 855.4)	(276.3, 772.5)	(377.4, 635.8)	(13.5, 51.8)	(5, 21.3)	(8.5, 18.2)	(7.9, 13.5)
48	Gallbladder and biliary tract cancer	553.7	536.5	389.9	925.9	10.6	7.4	7.8	11
		(233.4, 1424.4)	(223.1, 1089.2)	(209.2, 682.4)	(661, 1114.6)	(5.2, 19.8)	(5, 12.2)	(4.7, 12.1)	(8.1, 14.3)
49	Mesothelioma	1560.8	1123.4	779.1	906	46.6	55.9	49	29.5
		(578.3, 3489.3)	(675.8, 1736.8)	(400.2, 1376.1)	(707.4, 1236.5)	(21.3, 121.1)	(23.2, 151.9)	(16.1, 129.9)	(12.3, 61.4)
50	Neonatal preterm birth	3.9	1.8	4.6	5.2	0.1	0	0	0.1
		(2.3, 7.4)	(1.2, 2.7)	(2.9, 5.9)	(4.1, 6.7)	(0, 0.1)	(0, 0)	(0, 0.1)	(0, 0.1)
51	Nasopharynx cancer	260	303.7	535.6	828.4	3.9	6.1	9.9	10.1
		(98.9, 797.8)	(157.6, 539.4)	(228.8, 834)	(662.4, 1077.6)	(1.7, 11.5)	(3.5, 9.9)	(6.3, 15.8)	(7.4, 14.4)
52	Self-harm	5.7	5.7	4.8	1.9	0.2	0.4	0.2	0.1
		(2, 12.8)	(2.9, 10.5)	(2.7, 9.2)	(1.3, 2.4)	(0.1, 0.4)	(0.2, 0.7)	(0.1, 0.4)	(0.1, 0.1)

© 2025 Sahu M et al. JAMA Health Forum.

C. **eFigure 1:** ***Age- and prevalence-standardized pharmaceutical spending and utilization by race and ethnicity (for 52 health conditions)***

Here we present the full set of figures for all 52 health conditions with available prevalence data. Each figure is organized as follows:

- (I) The top row represents utilization (prescription fills) and (II) the bottom row represents pharmaceutical spending.
- (i) The left column is non-age-standardized per capita estimate, (ii) the middle column is the age-standardized per capita estimate, and (iii) the right column is the age- and prevalence-standardized estimate (i.e. age-standardized fills/spending per prevalent case)

Health conditions are ranked by total retail pharmaceutical spending, as shown in Table S3.2.

(1) Diabetes mellitus type 2

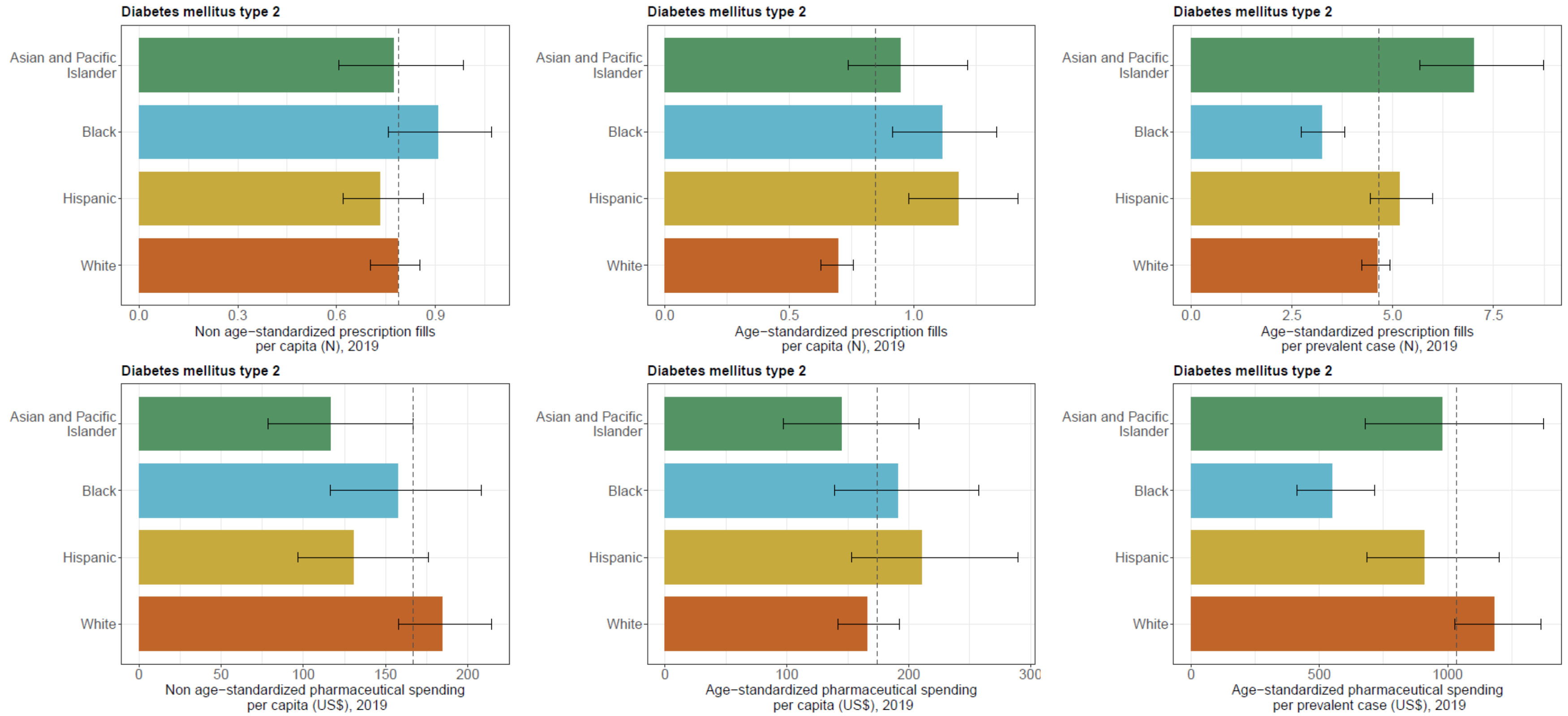

© 2025 Sahu M et al. JAMA Health Forum.

(2) HIV/AIDS

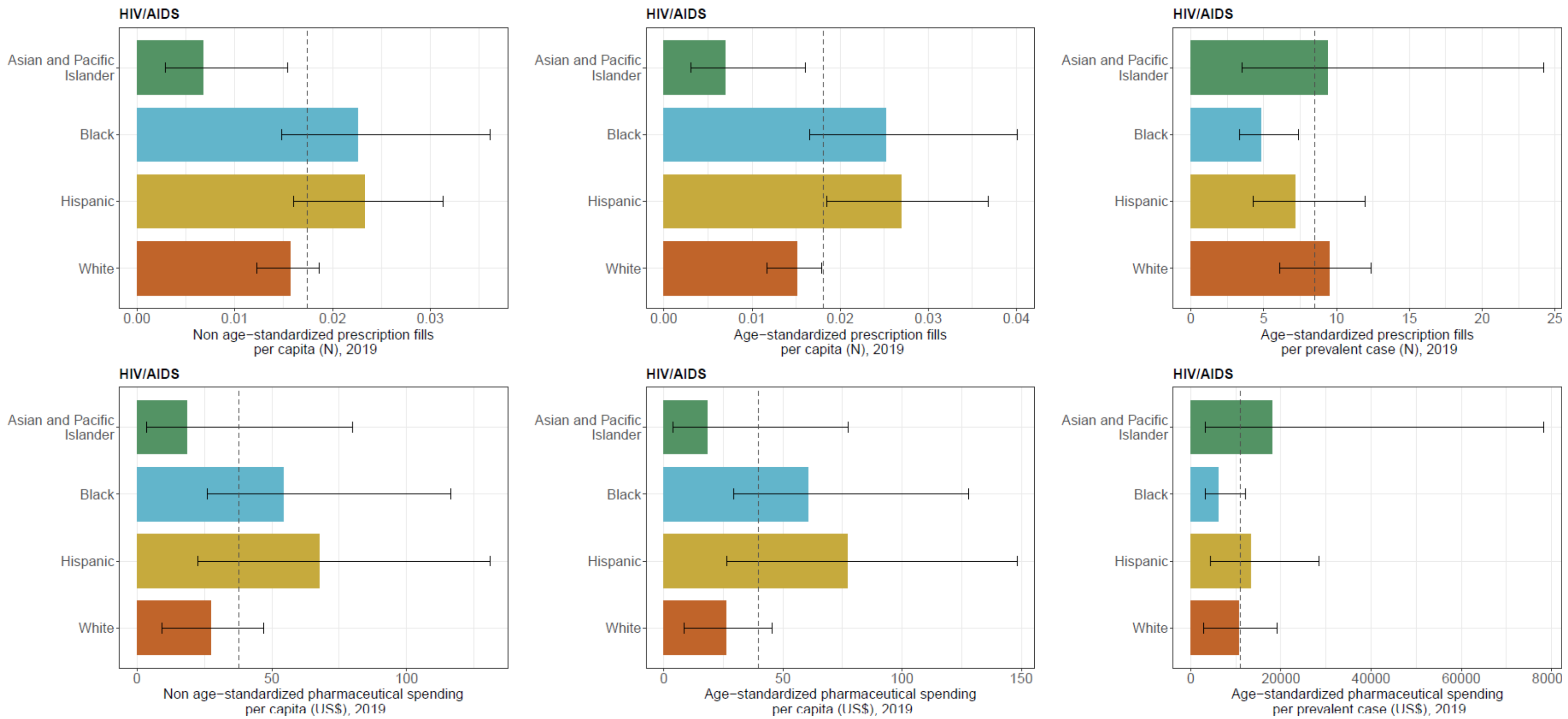

Note: Utilization/spending is inclusive of preventive therapy for HIV (PrEP), so 'per prevalent case' estimates should be interpreted with caution.

© 2025 Sahu M et al. JAMA Health Forum.

(3) Multiple sclerosis

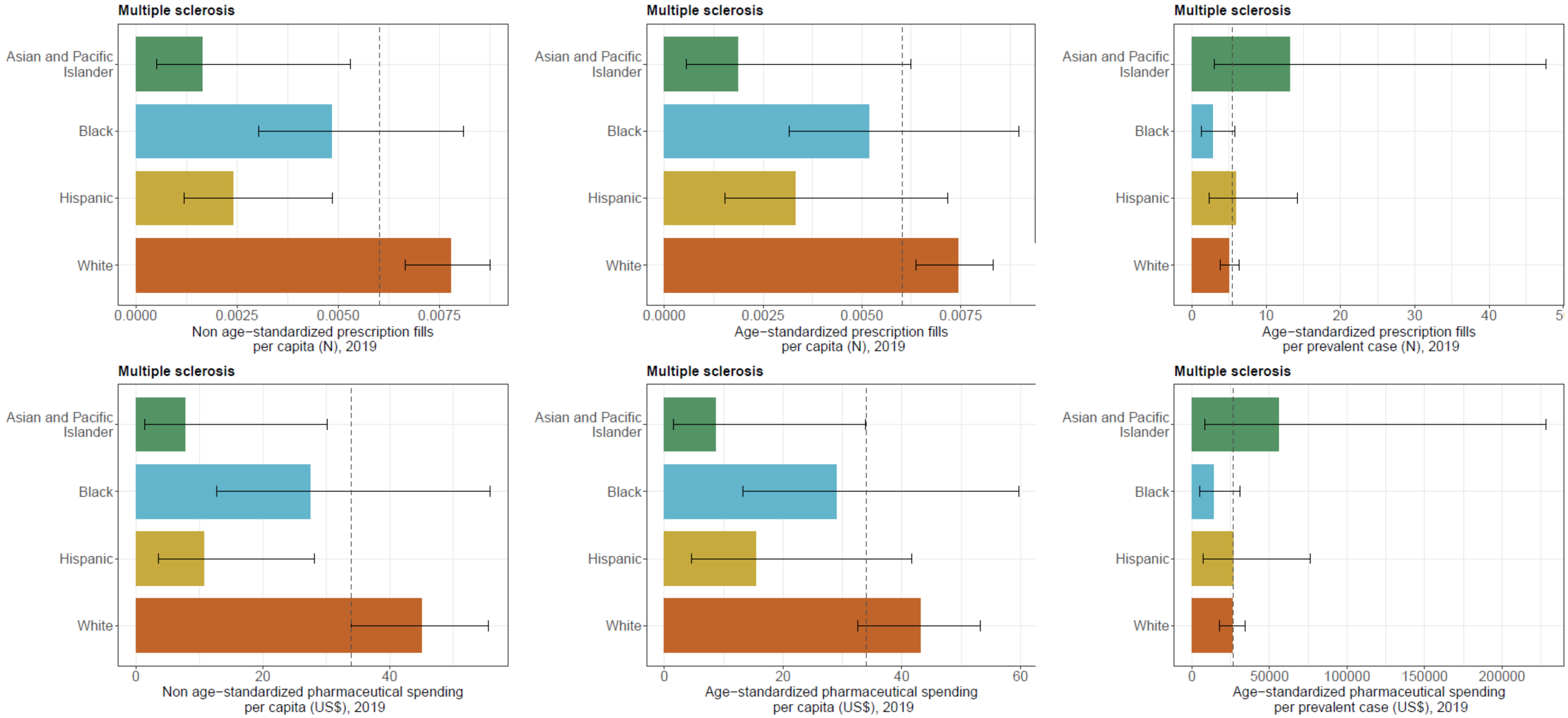

© 2025 Sahu M et al. JAMA Health Forum.

(4) Atrial fibrillation and flutter

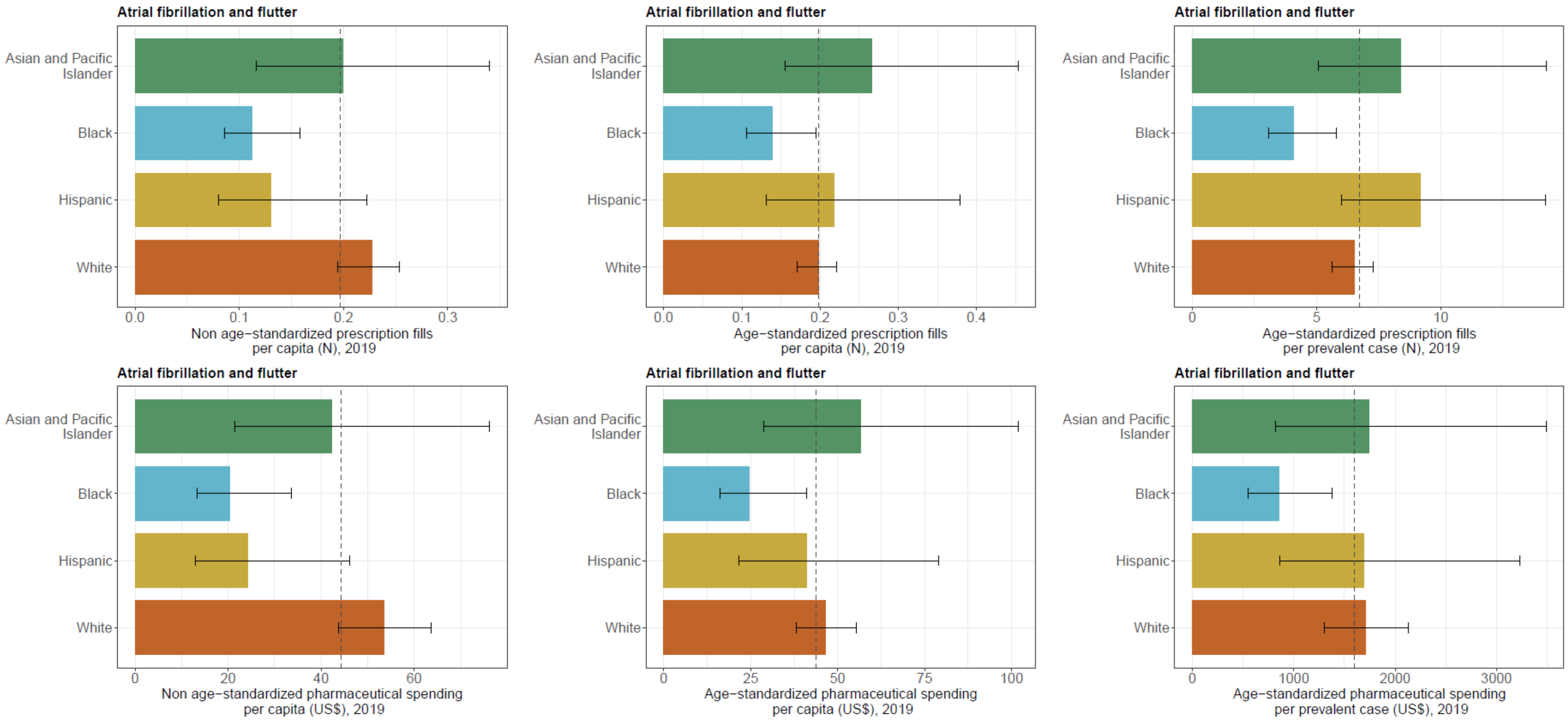

© 2025 Sahu M et al. JAMA Health Forum.

(5) Rheumatoid arthritis

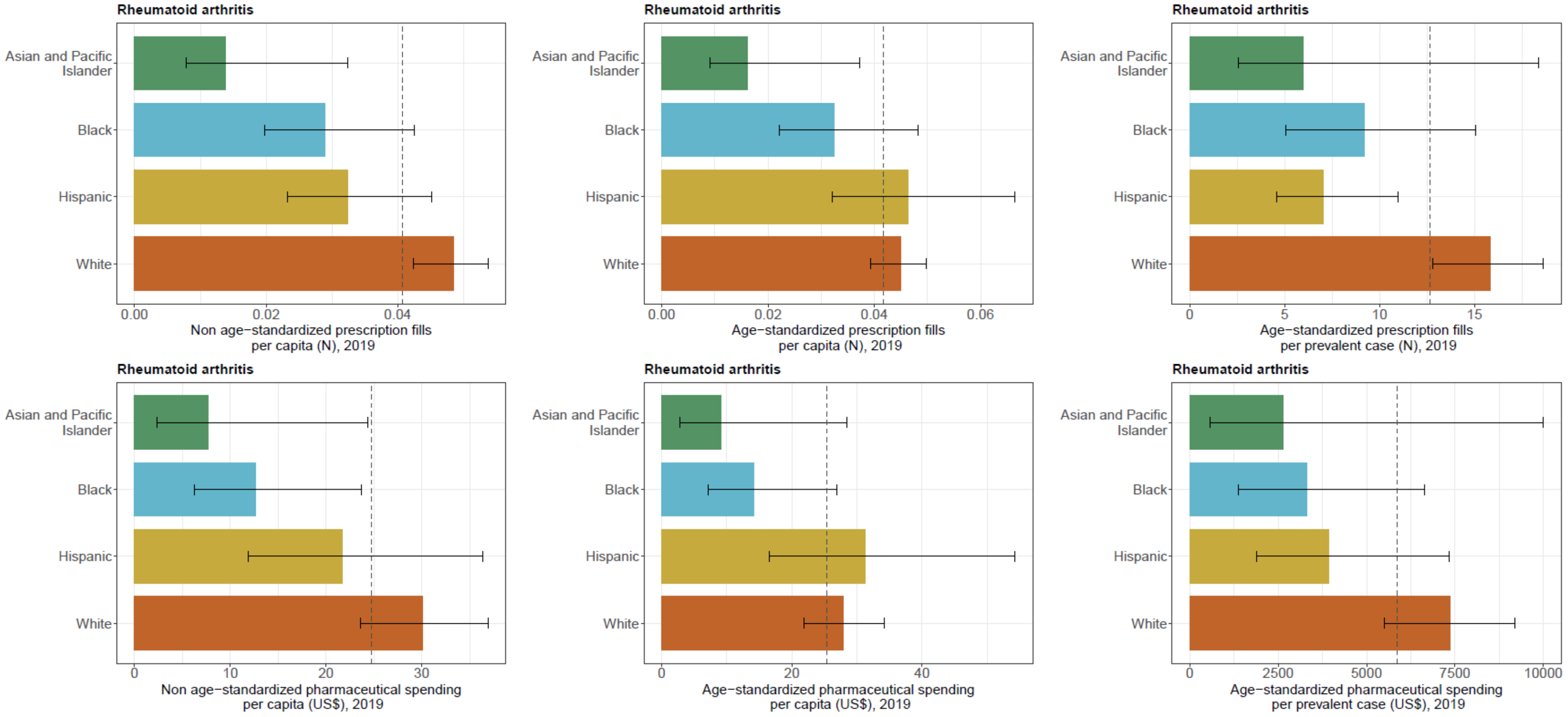

© 2025 Sahu M et al. JAMA Health Forum.

(6) Chronic obstructive pulmonary disease

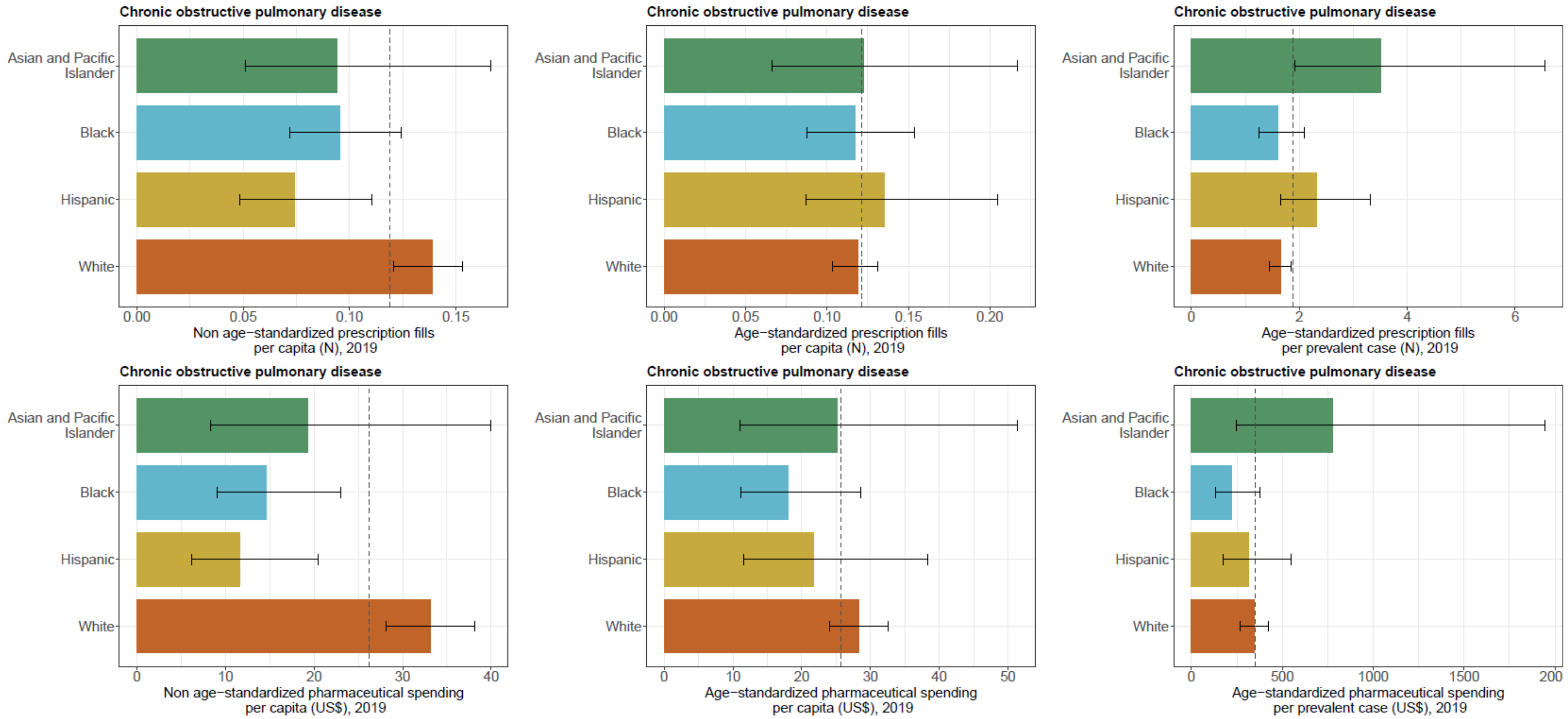

© 2025 Sahu M et al. JAMA Health Forum.

(7) Leukemia

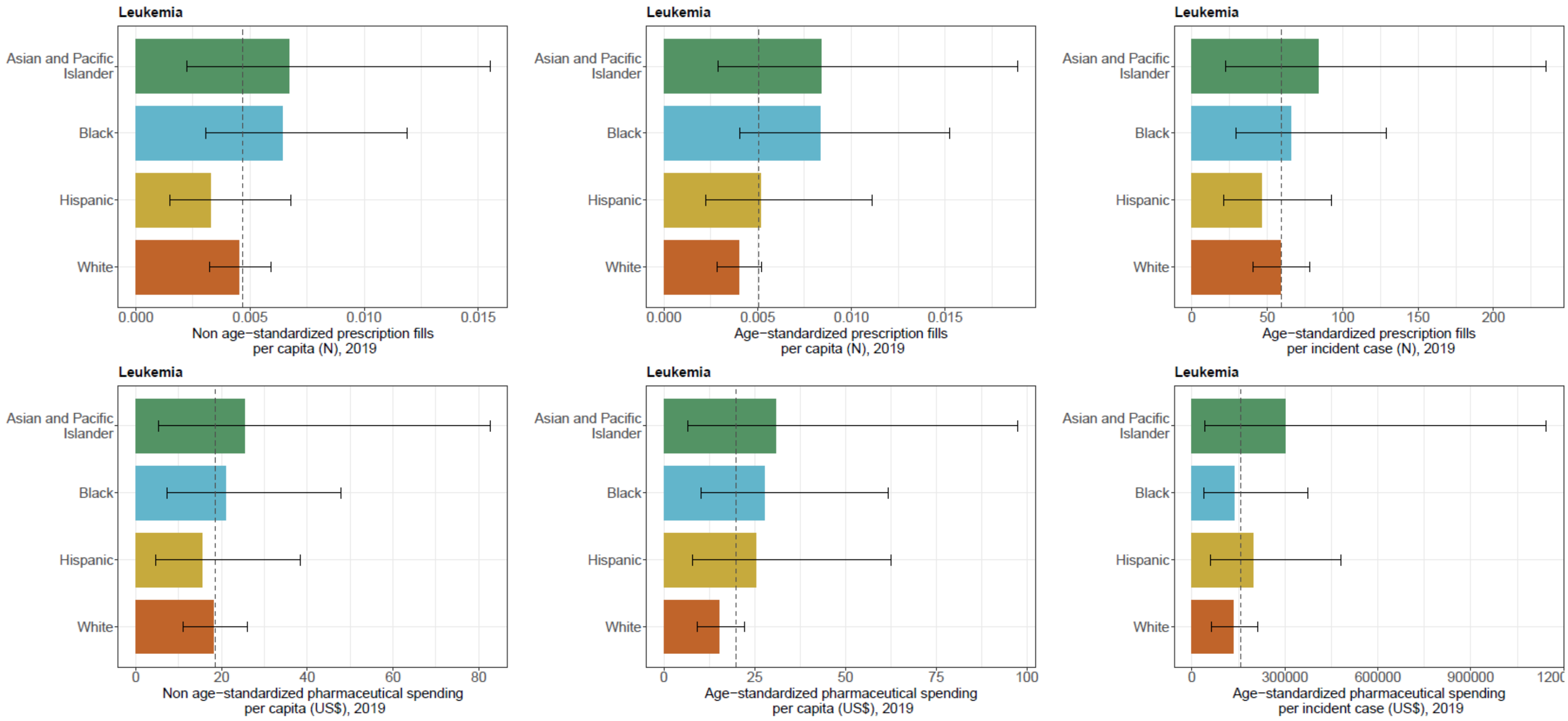

© 2025 Sahu M et al. JAMA Health Forum.

(8) Multiple myeloma

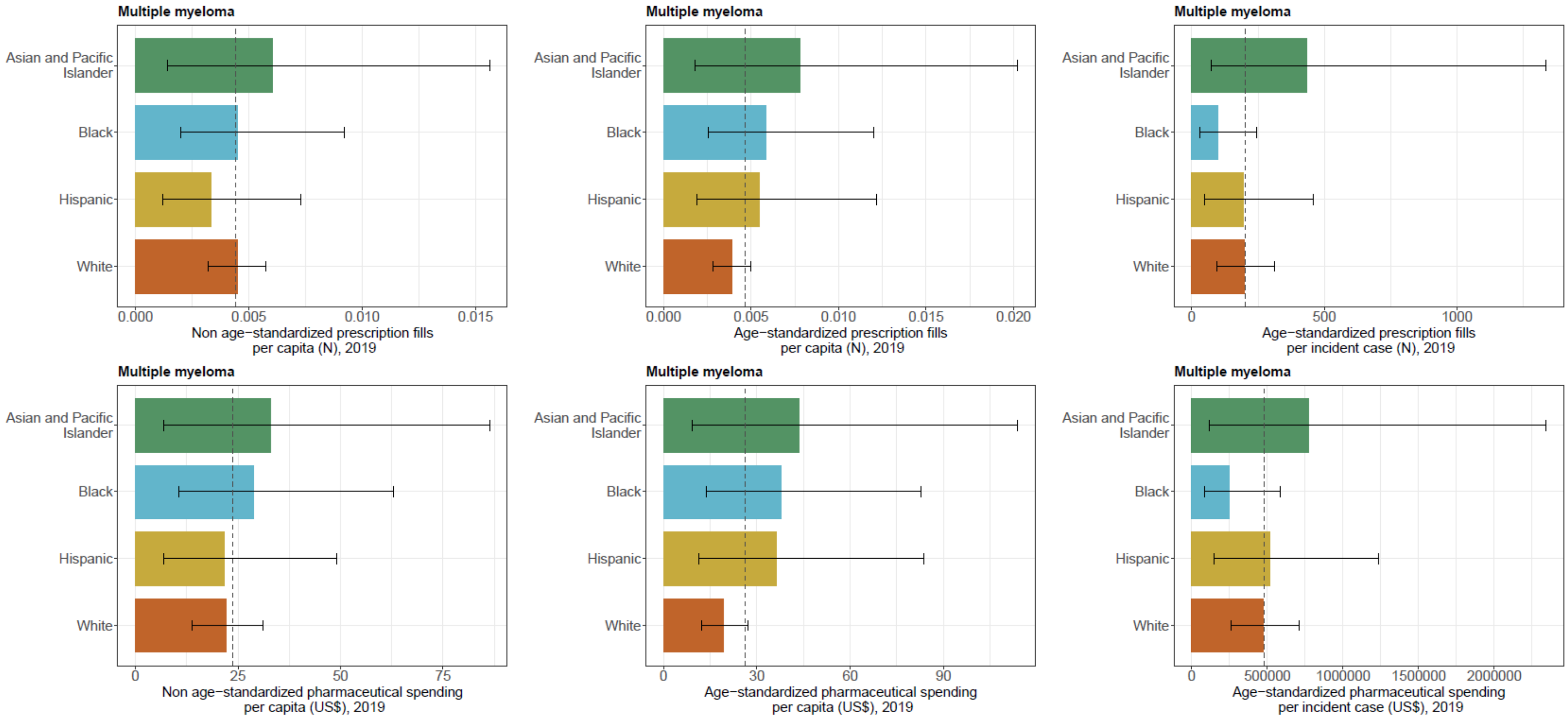

© 2025 Sahu M et al. JAMA Health Forum.

(9) Breast cancer

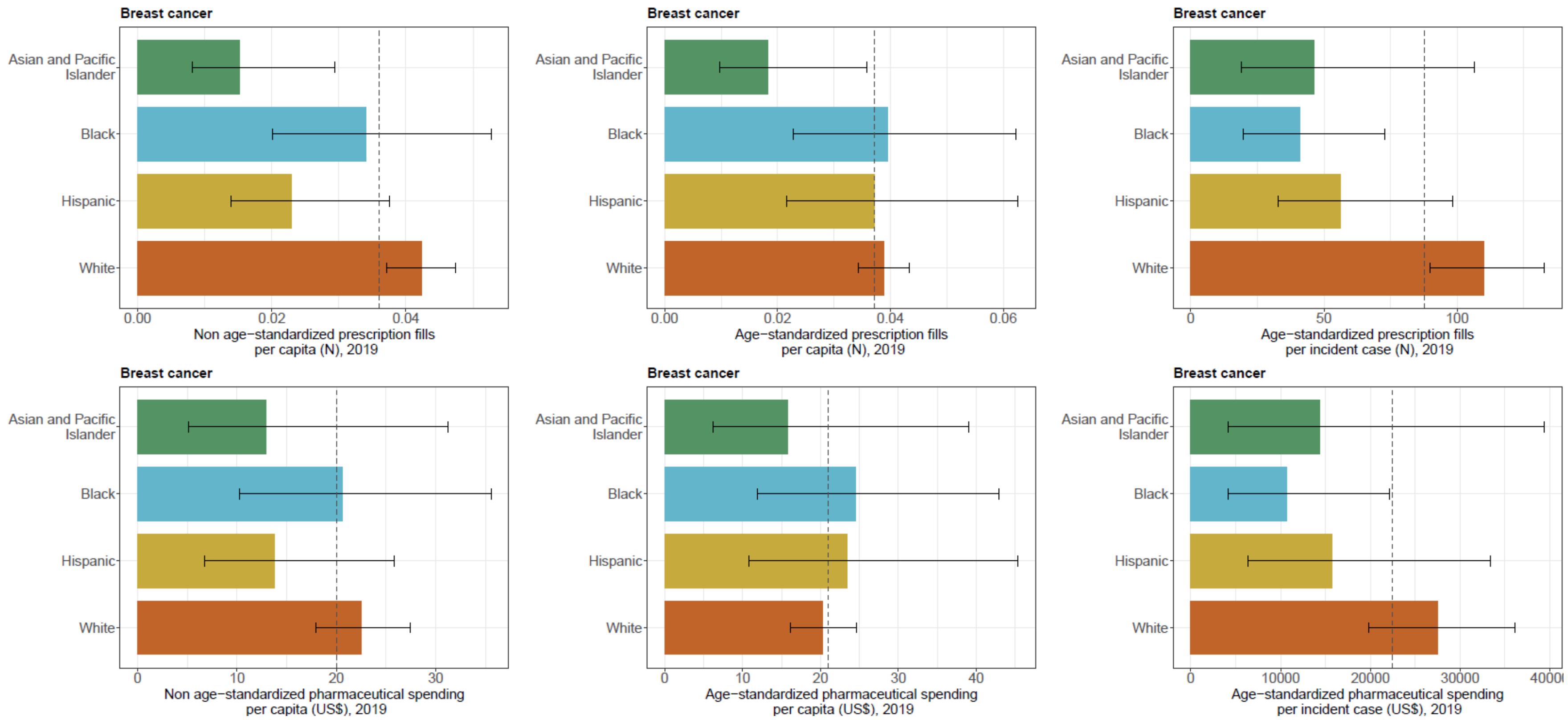

© 2025 Sahu M et al. JAMA Health Forum.

(10) Ischemic heart disease

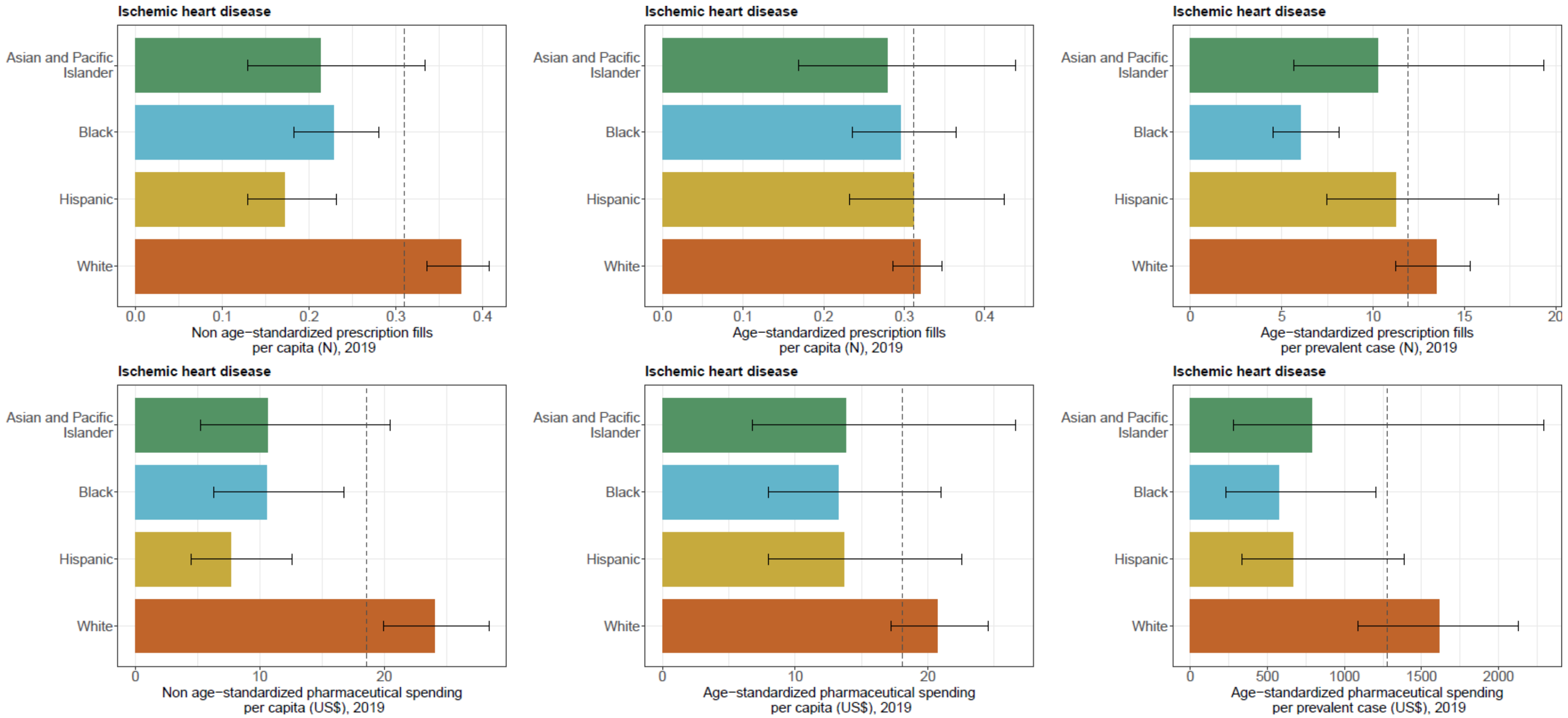

© 2025 Sahu M et al. JAMA Health Forum.

(11) Prostate cancer

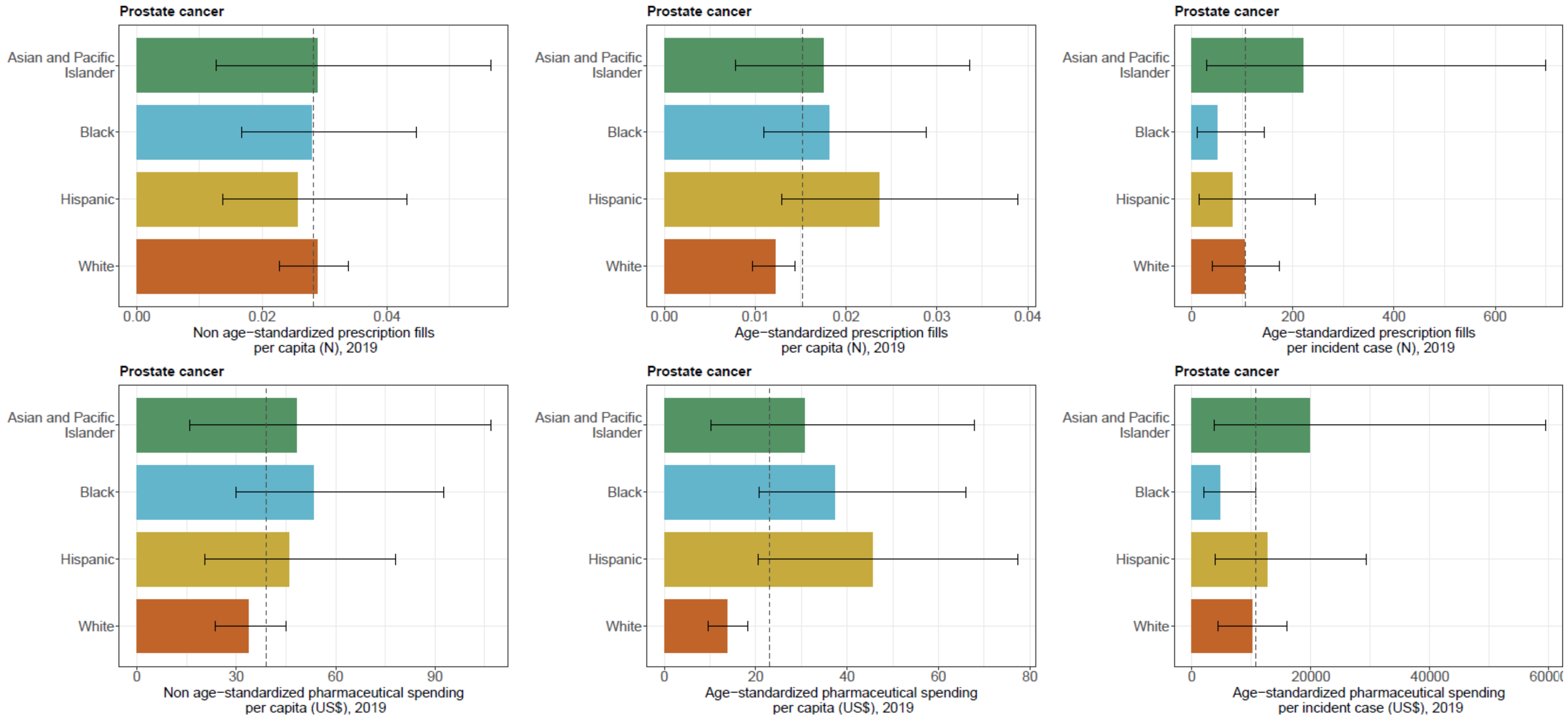

© 2025 Sahu M et al. JAMA Health Forum.

(12) Tracheal, bronchus, and lung cancer

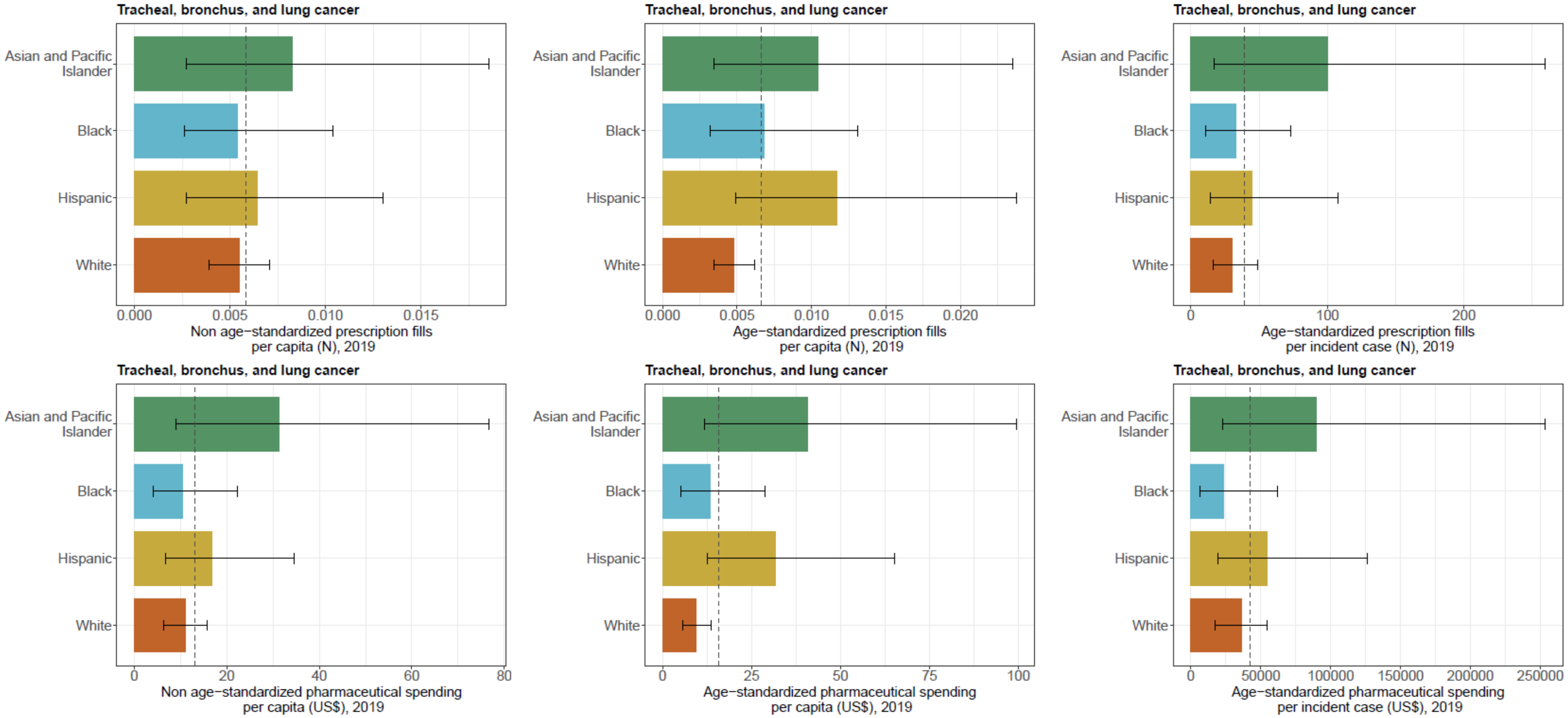

© 2025 Sahu M et al. JAMA Health Forum.

(13) Non-Hodgkin lymphoma

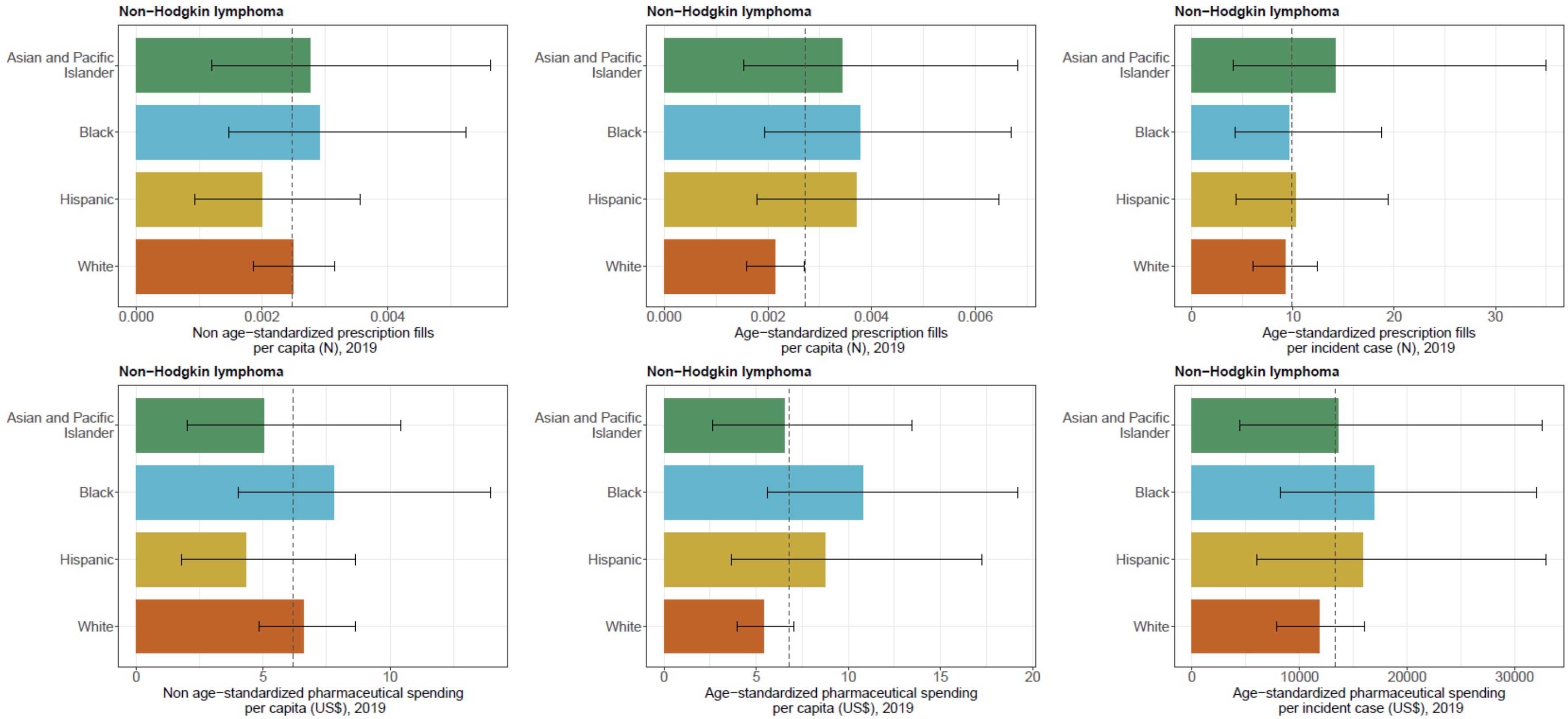

© 2025 Sahu M et al. JAMA Health Forum.

(14) Kidney cancer

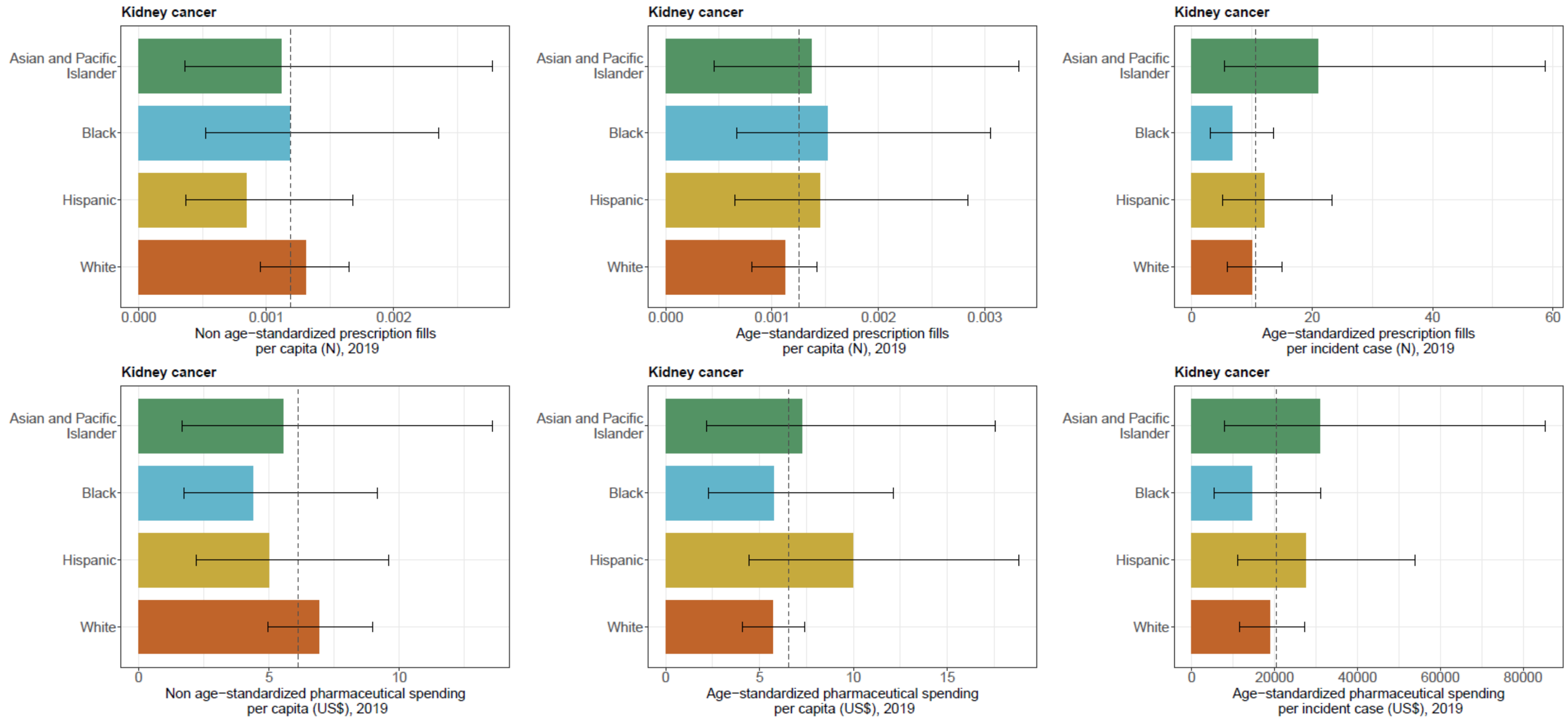

© 2025 Sahu M et al. JAMA Health Forum.

(15) Lower respiratory infections

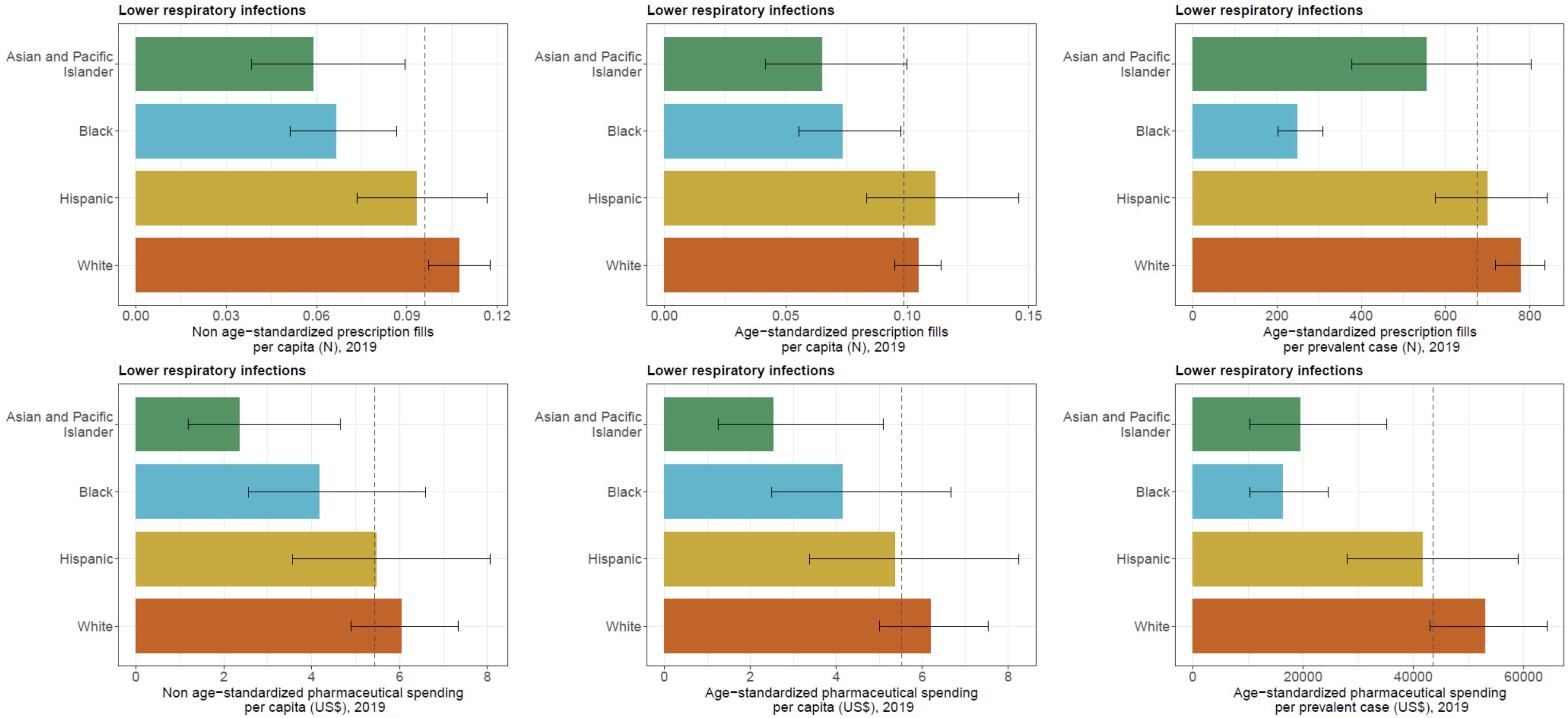

© 2025 Sahu M et al. JAMA Health Forum.

(16) Opioid use disorders

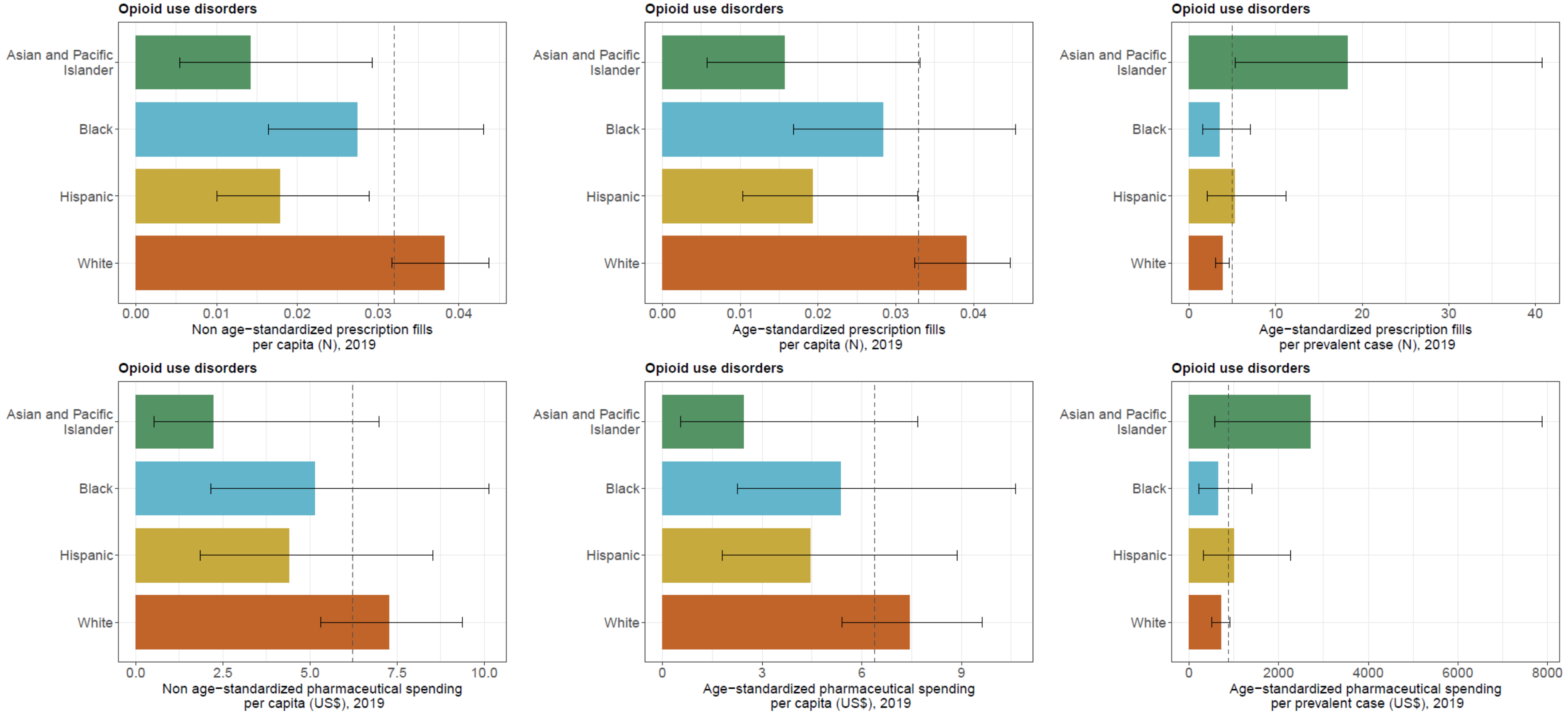

© 2025 Sahu M et al. JAMA Health Forum.

(17) Stroke

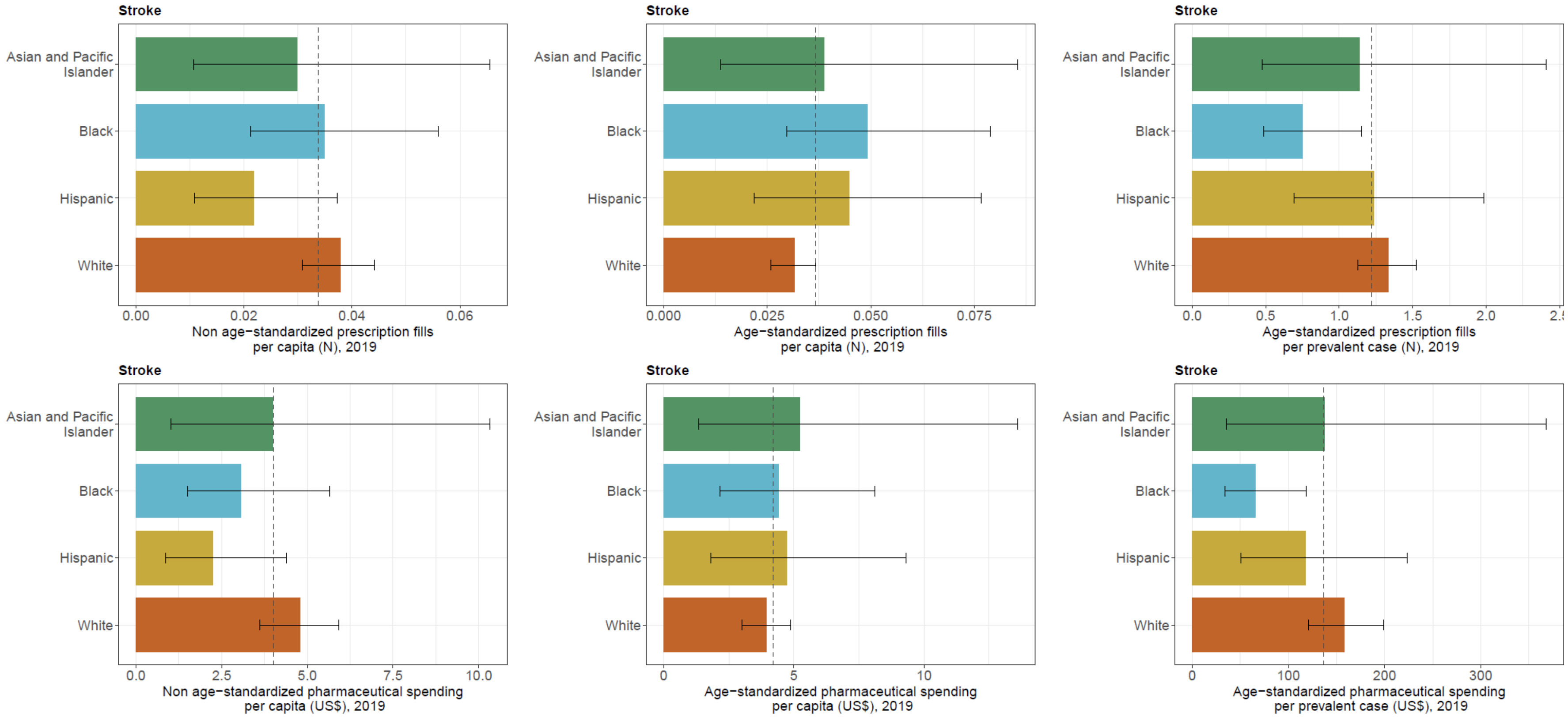

© 2025 Sahu M et al. JAMA Health Forum.

(18) Nutritional deficiencies

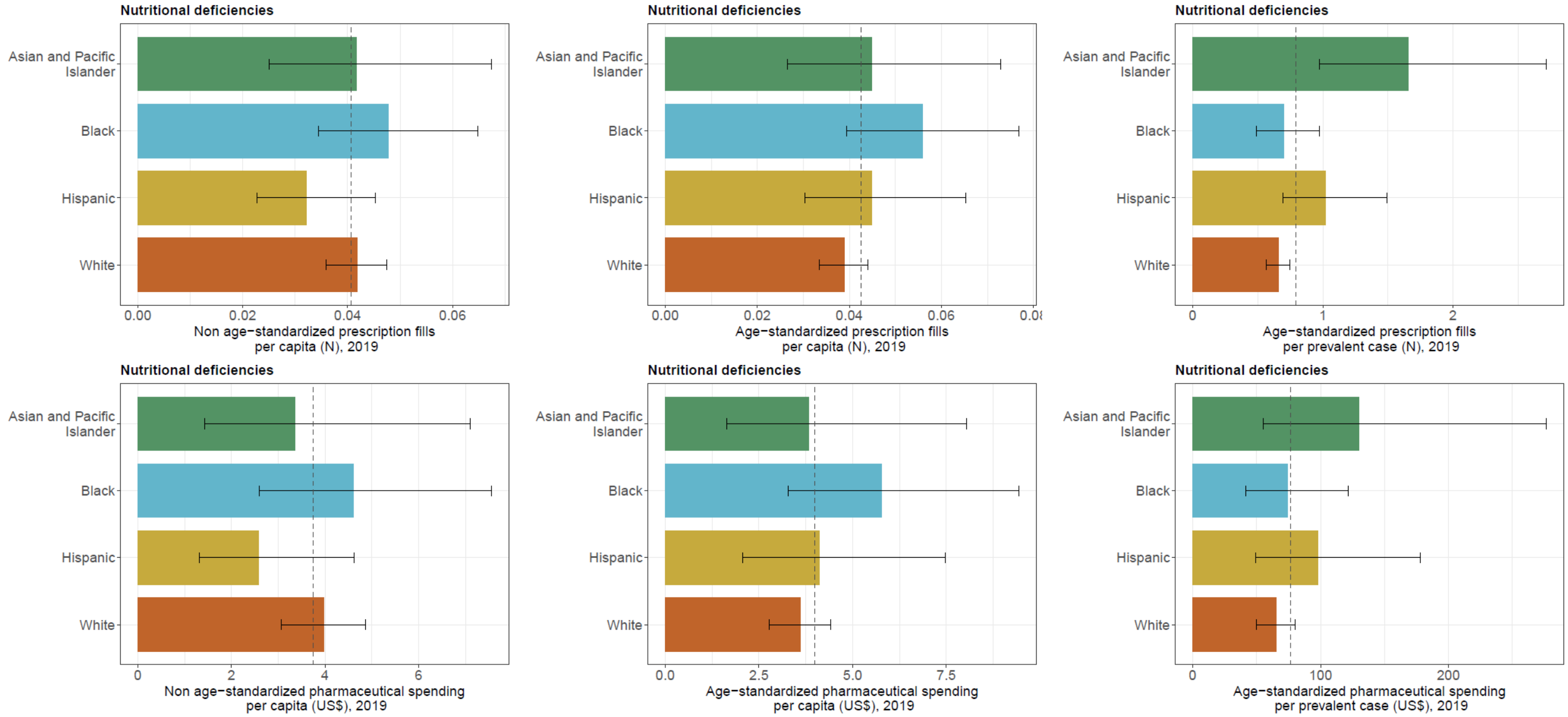

© 2025 Sahu M et al. JAMA Health Forum.

(19) Colon and rectum cancer

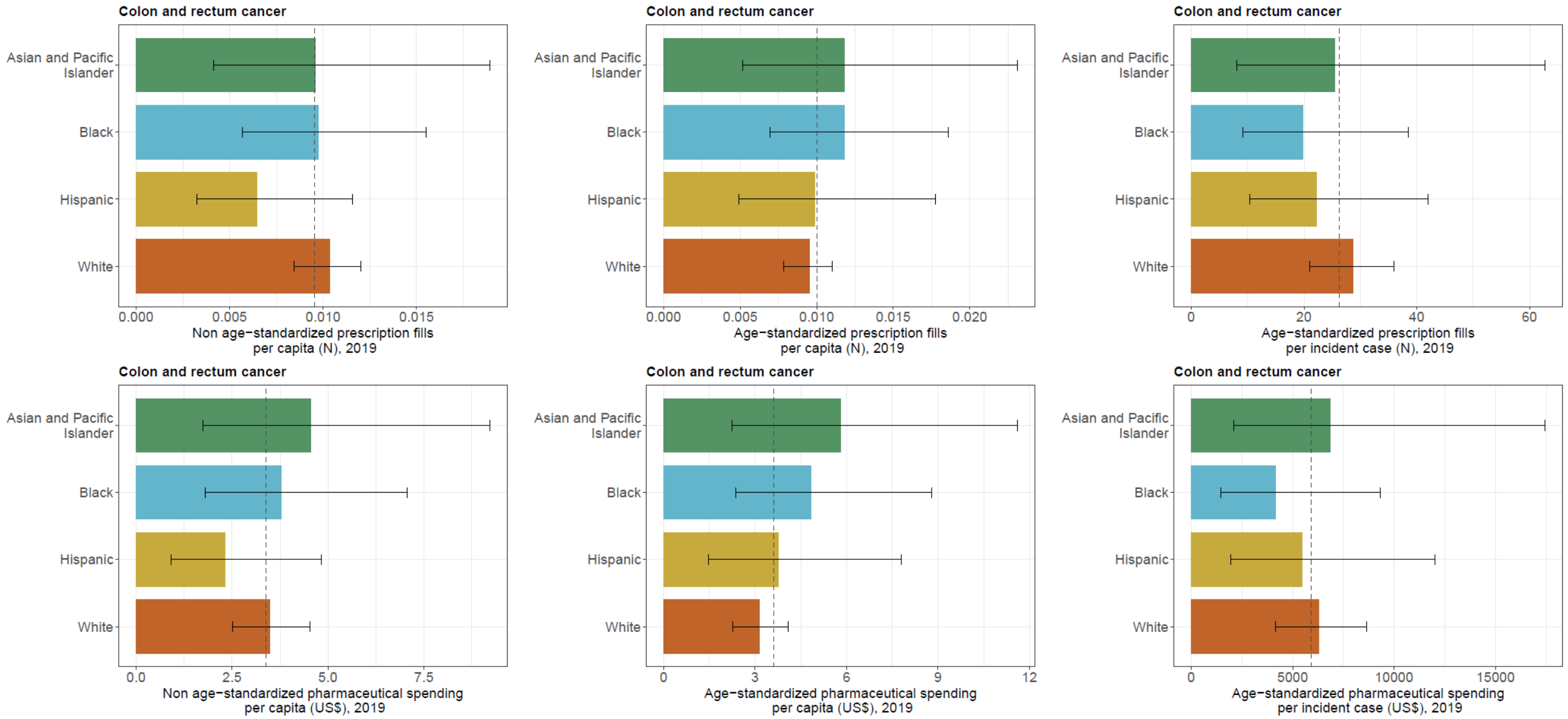

© 2025 Sahu M et al. JAMA Health Forum.

(20) Liver cancer

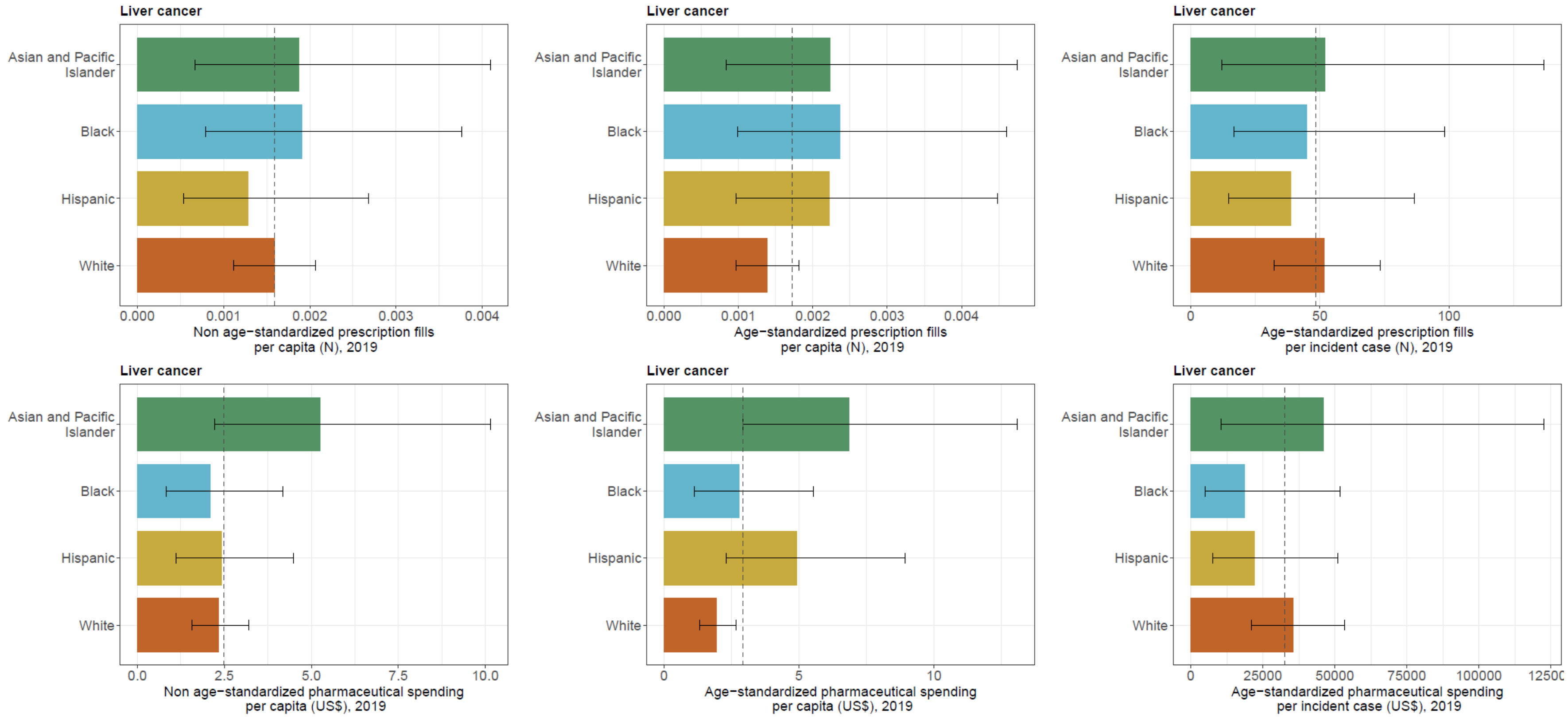

© 2025 Sahu M et al. JAMA Health Forum.

(21) Ovarian cancer

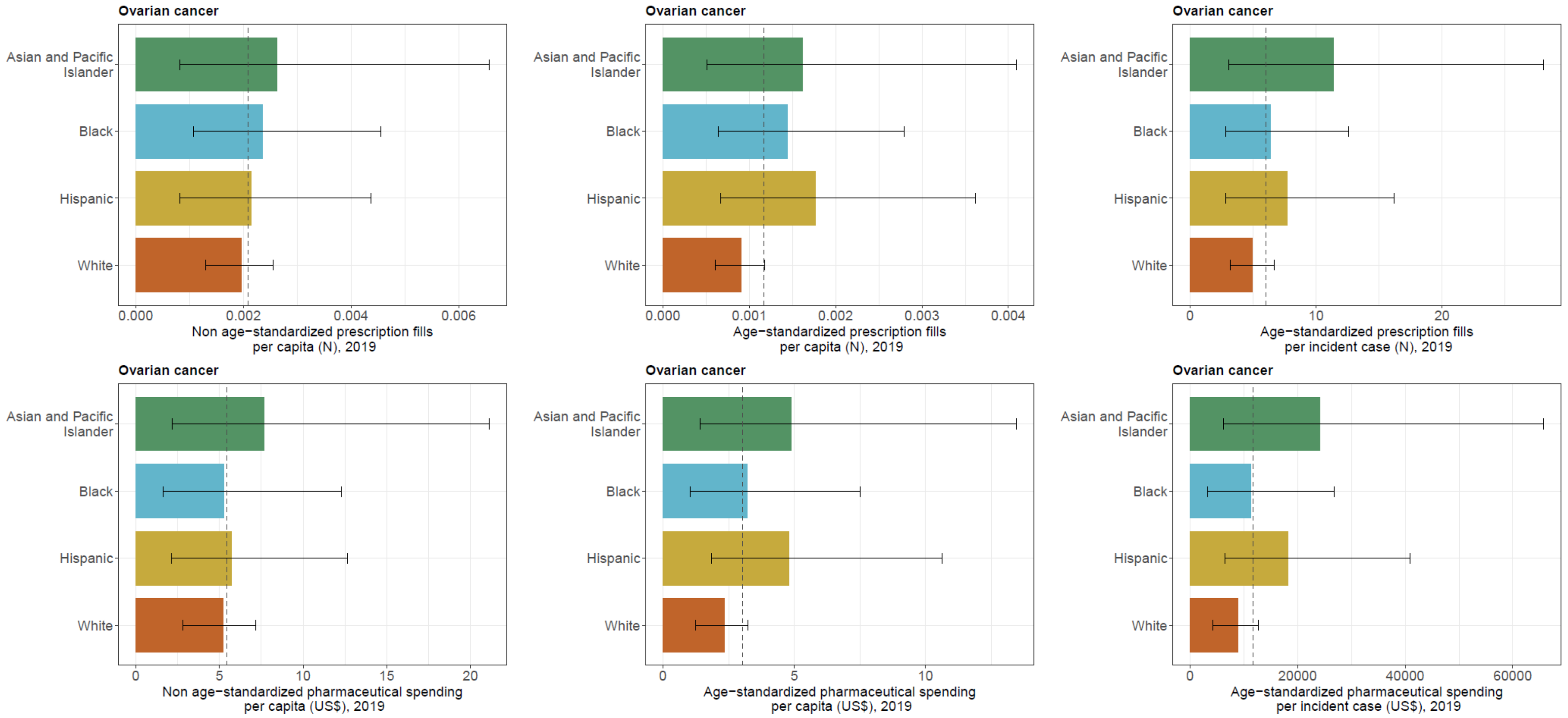

© 2025 Sahu M et al. JAMA Health Forum.

(22) Malignant neoplasm of bone and articular cartilage

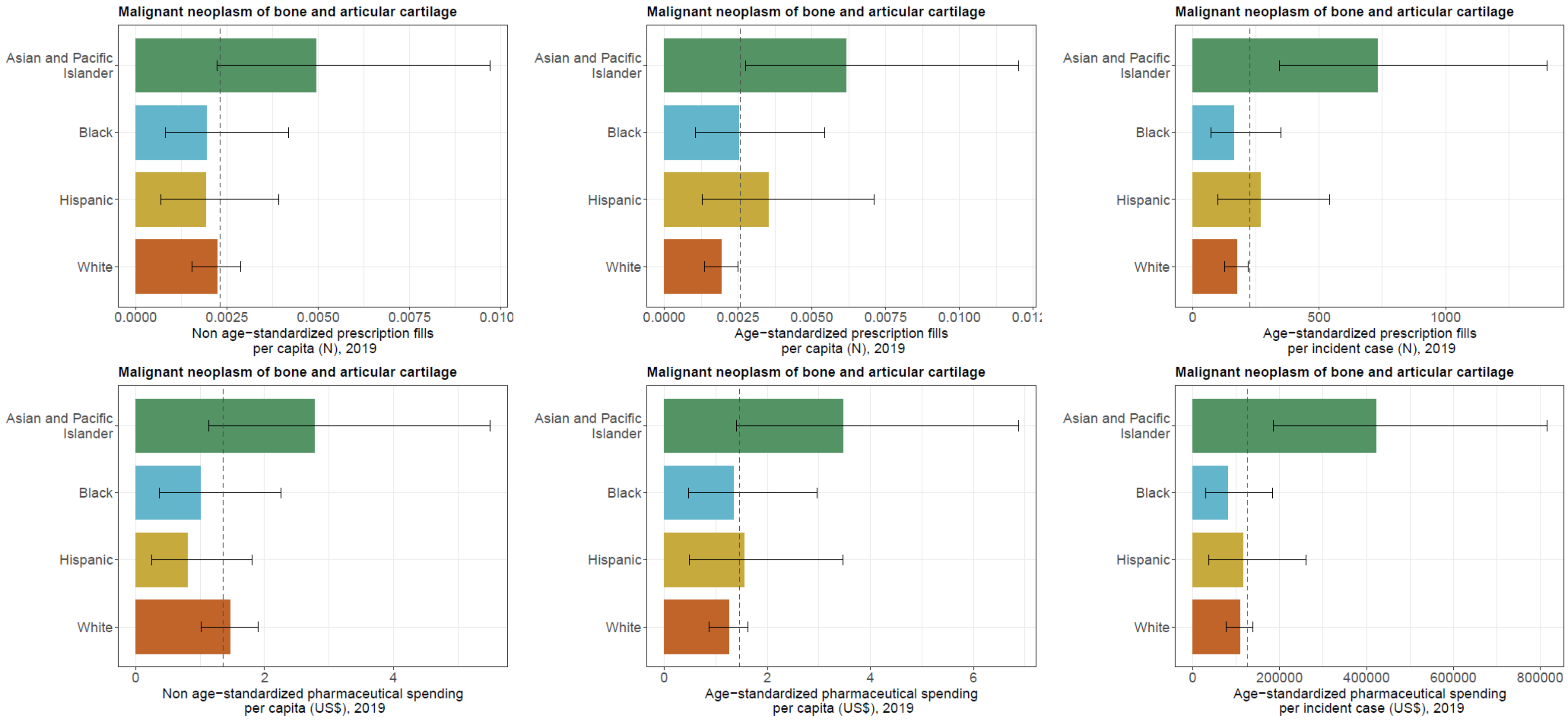

© 2025 Sahu M et al. JAMA Health Forum.

(23) Cardiomyopathy and myocarditis

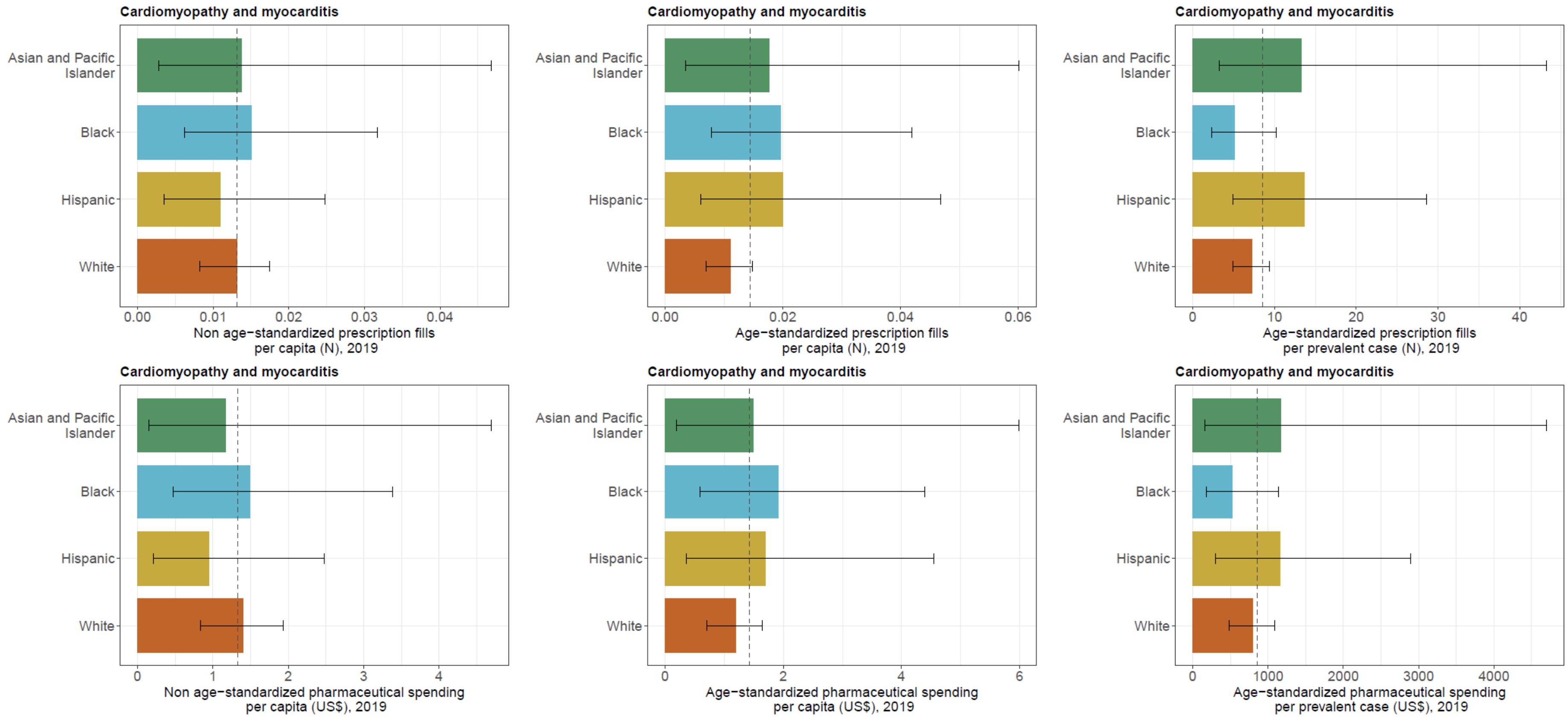

© 2025 Sahu M et al. JAMA Health Forum.

(24) Malignant skin melanoma

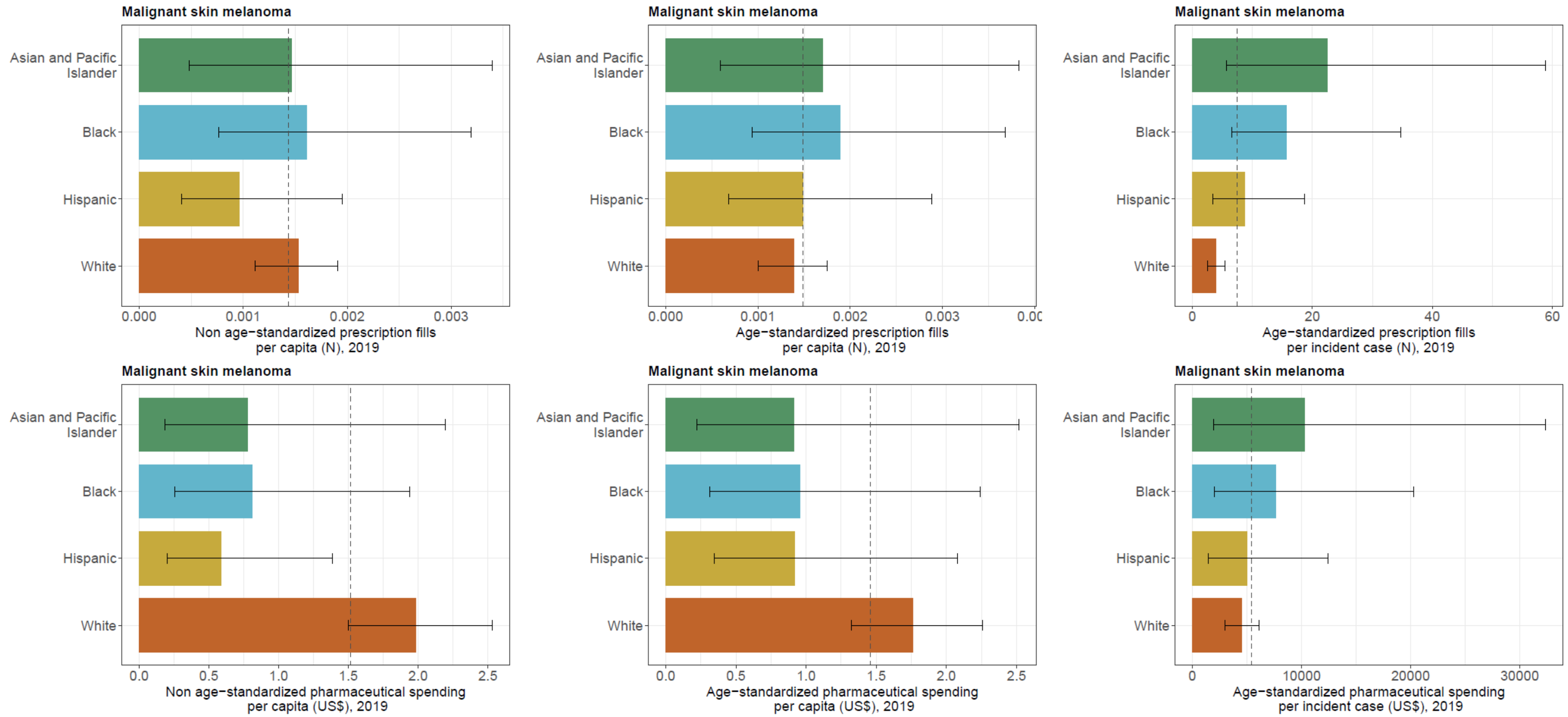

© 2025 Sahu M et al. JAMA Health Forum.

(25) Hypertensive heart disease

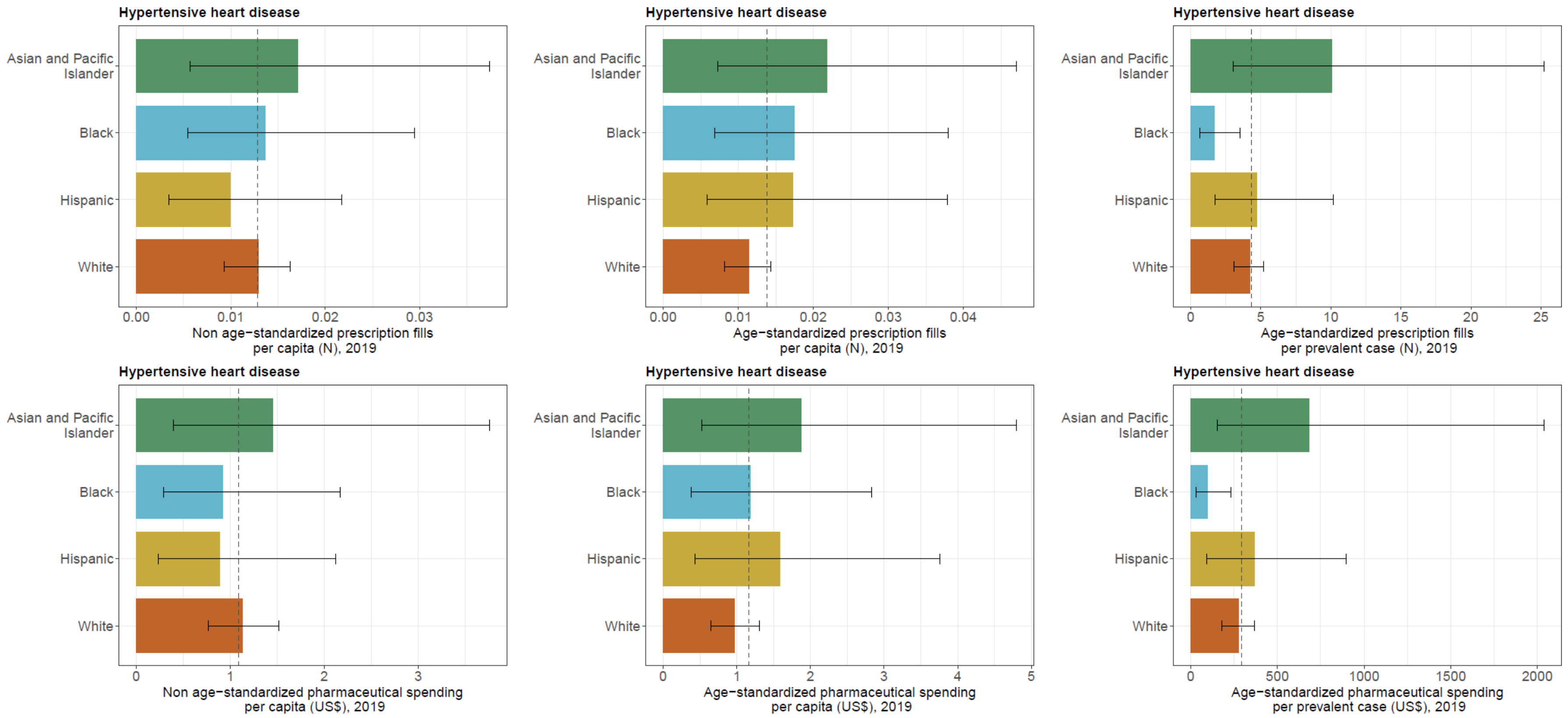

© 2025 Sahu M et al. JAMA Health Forum.

(26) Brain and central nervous system cancer

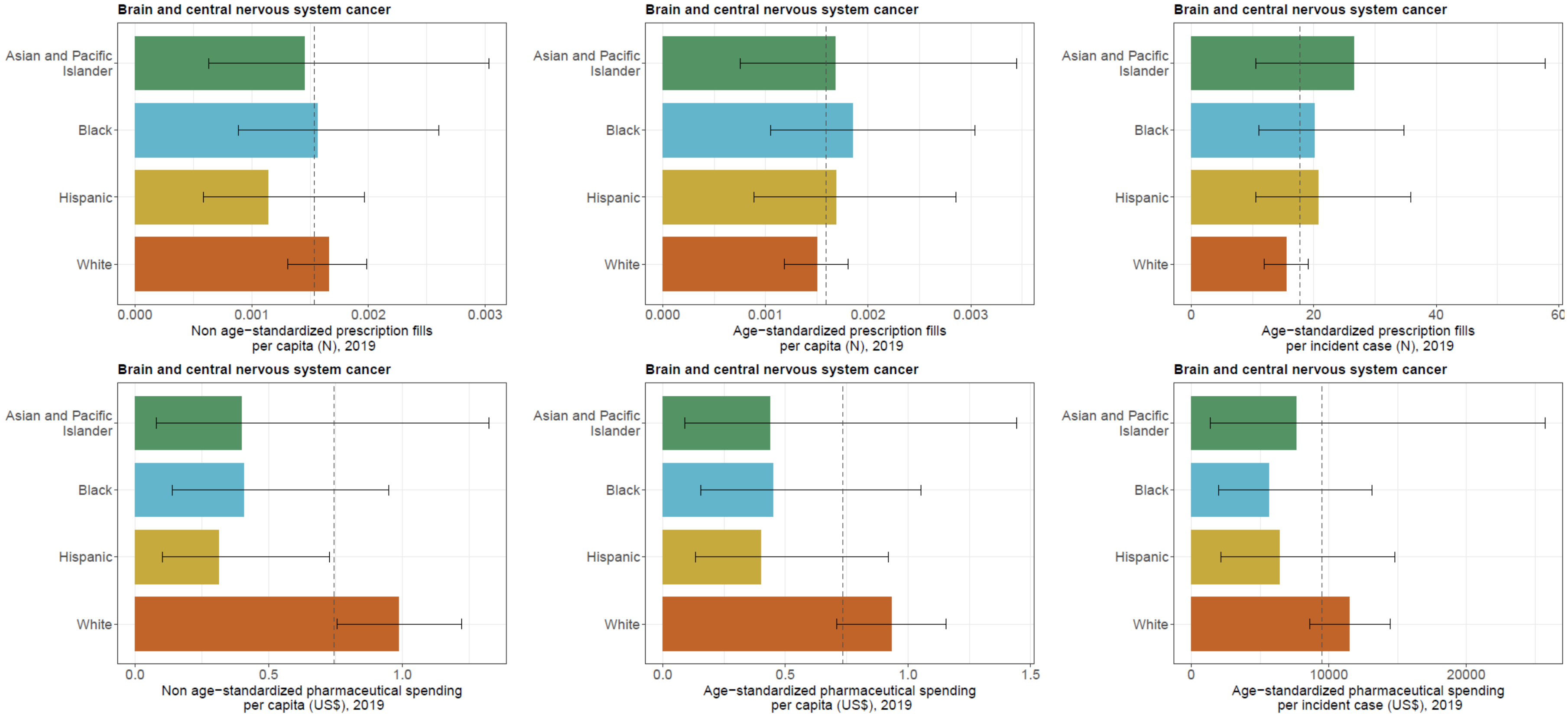

© 2025 Sahu M et al. JAMA Health Forum.

(27) Thyroid cancer

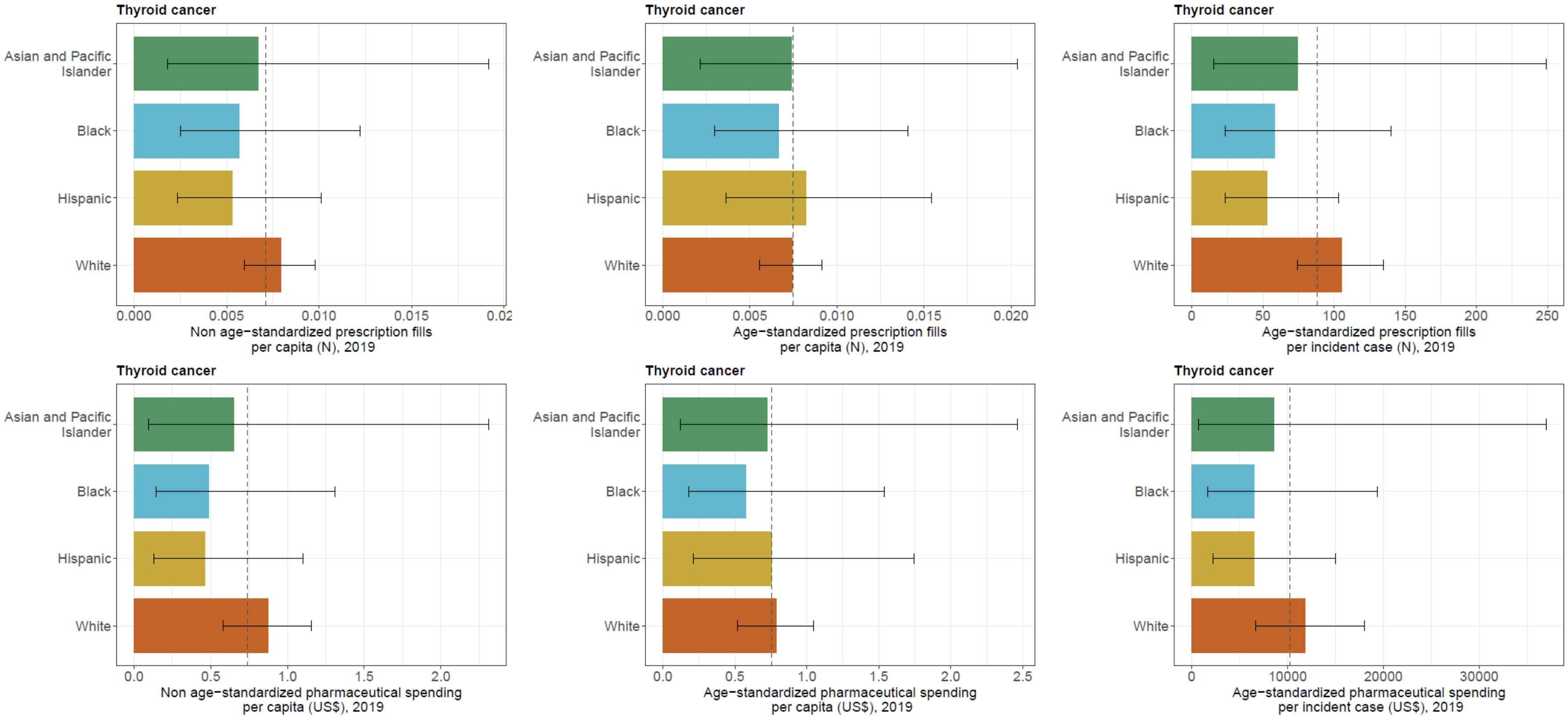

© 2025 Sahu M et al. JAMA Health Forum.

(28) Inguinal, femoral, and abdominal hernia

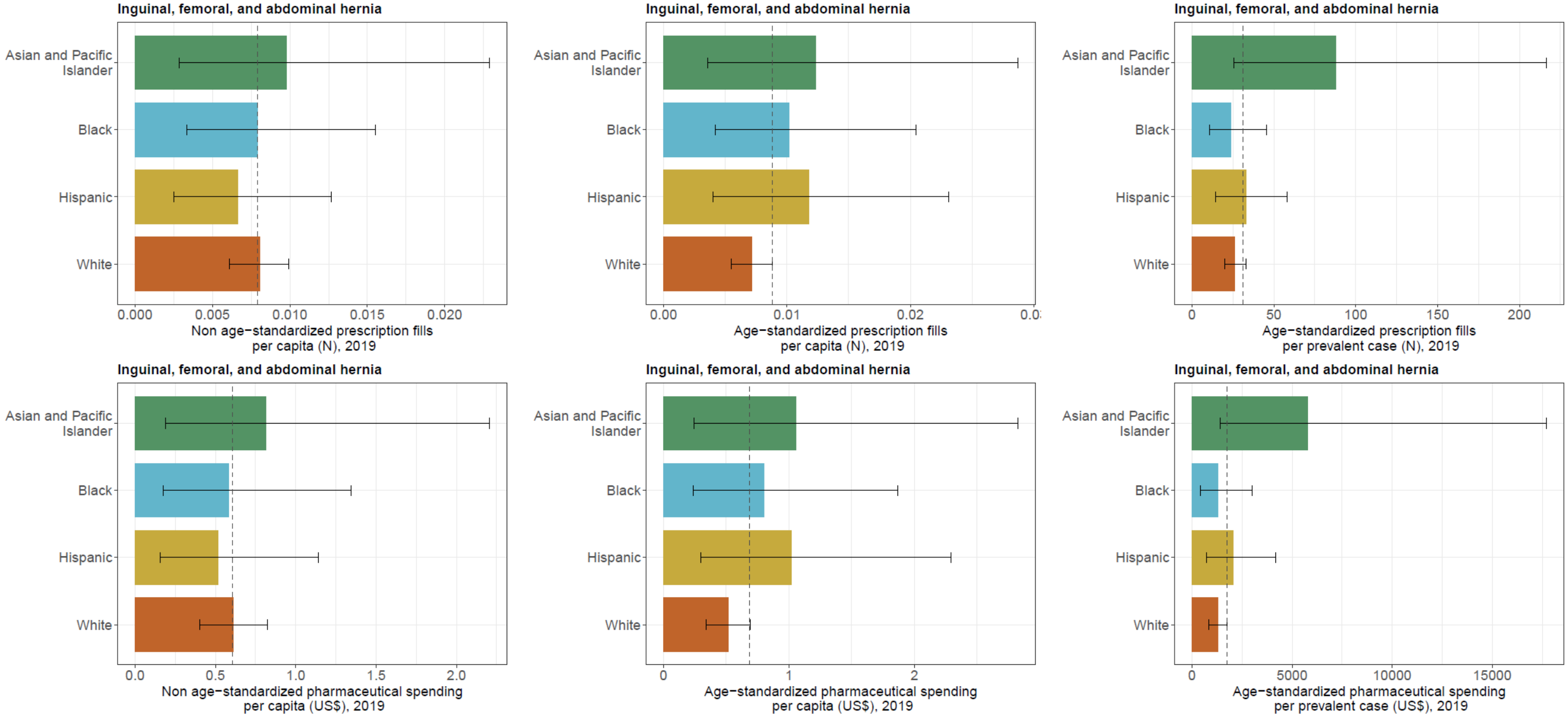

© 2025 Sahu M et al. JAMA Health Forum.

(29) Alcohol use disorders

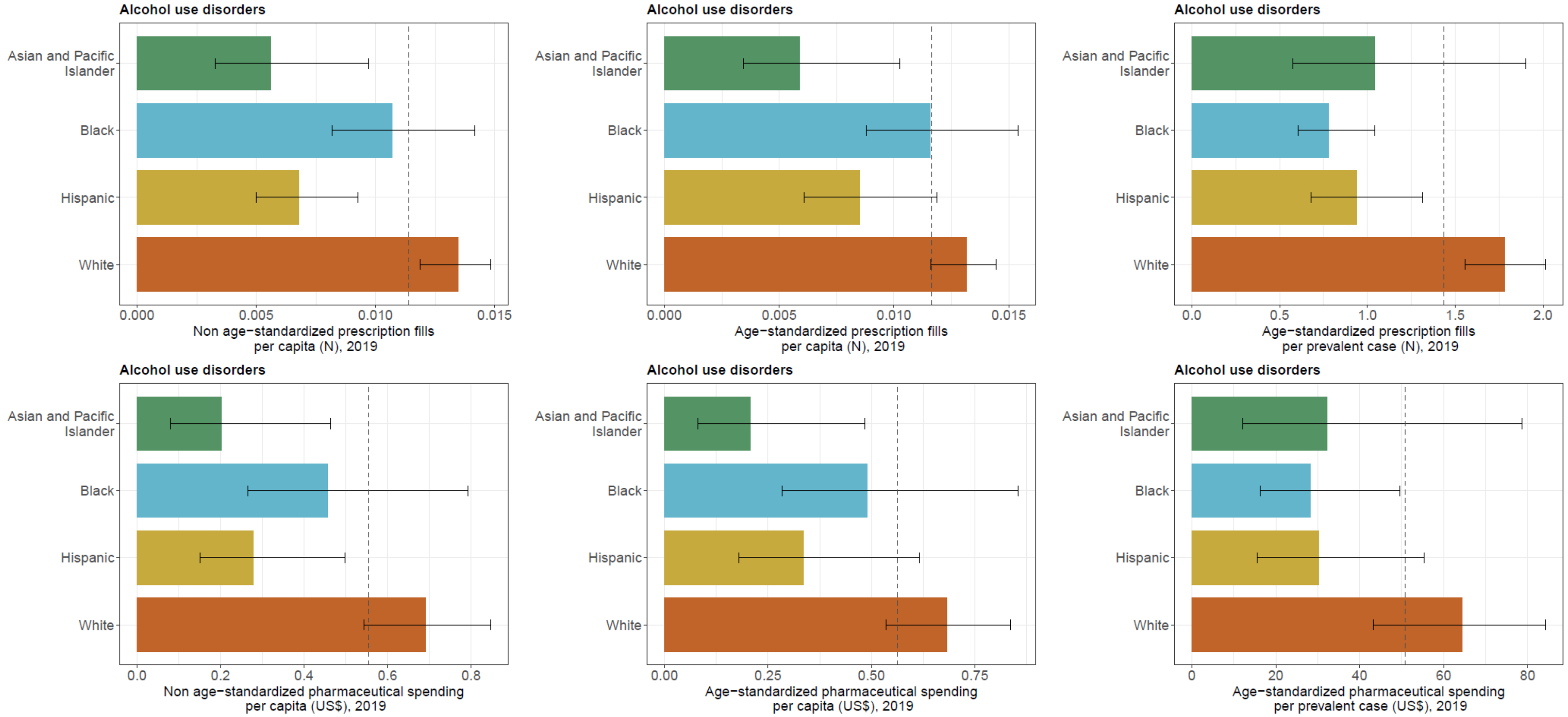

© 2025 Sahu M et al. JAMA Health Forum.

(30) Soft tissue and other extraosseous sarcomas

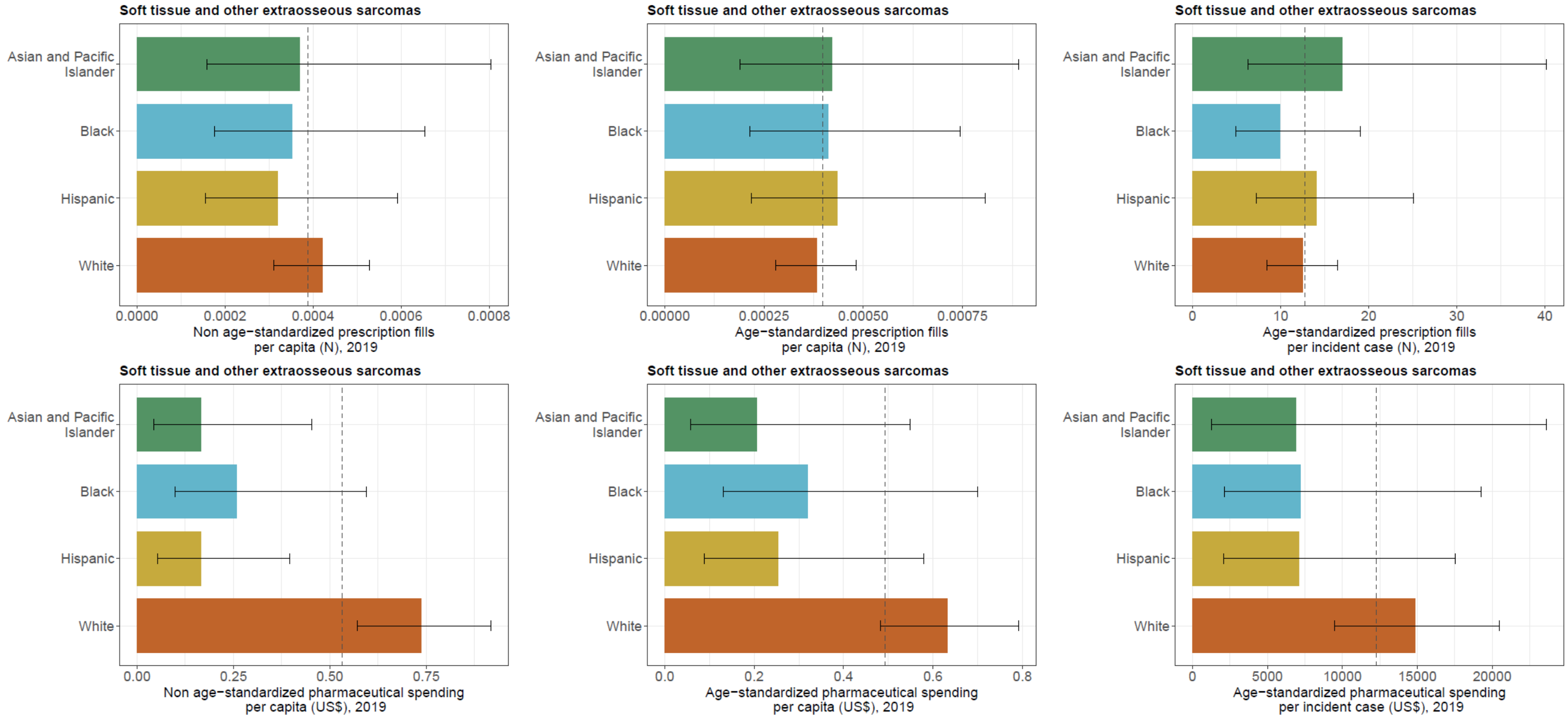

© 2025 Sahu M et al. JAMA Health Forum.

(31) Other malignant neoplasms

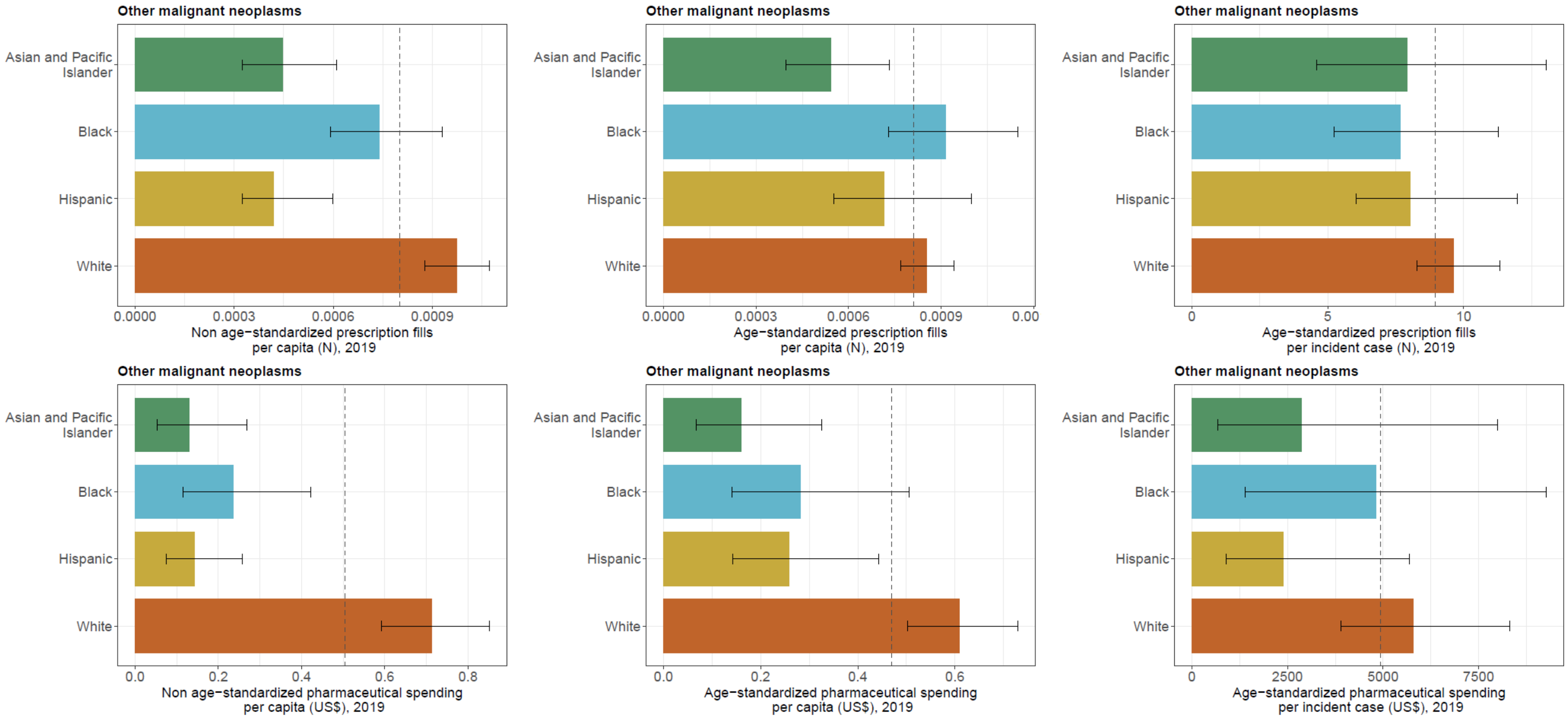

© 2025 Sahu M et al. JAMA Health Forum.

(32) Pancreatic cancer

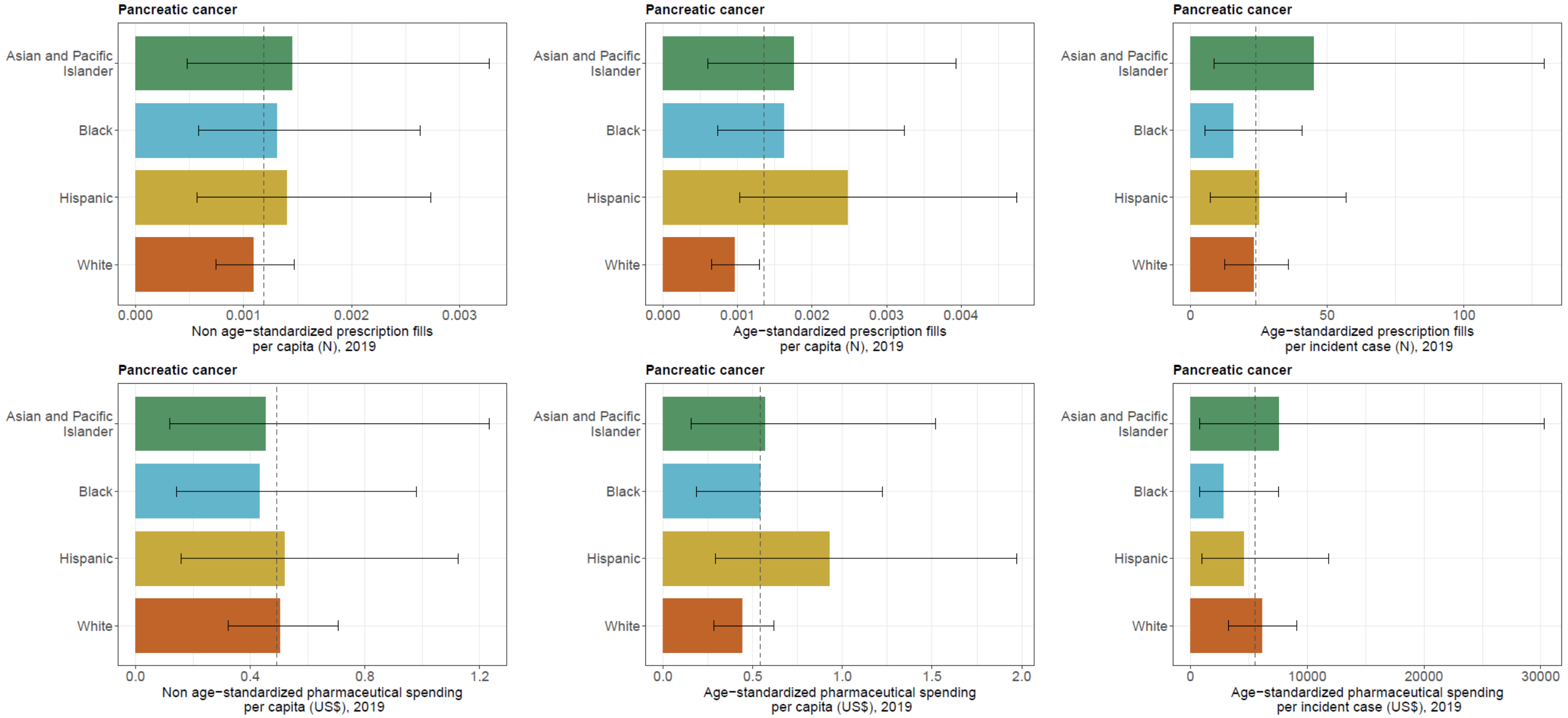

© 2025 Sahu M et al. JAMA Health Forum.

(33) Uterine cancer

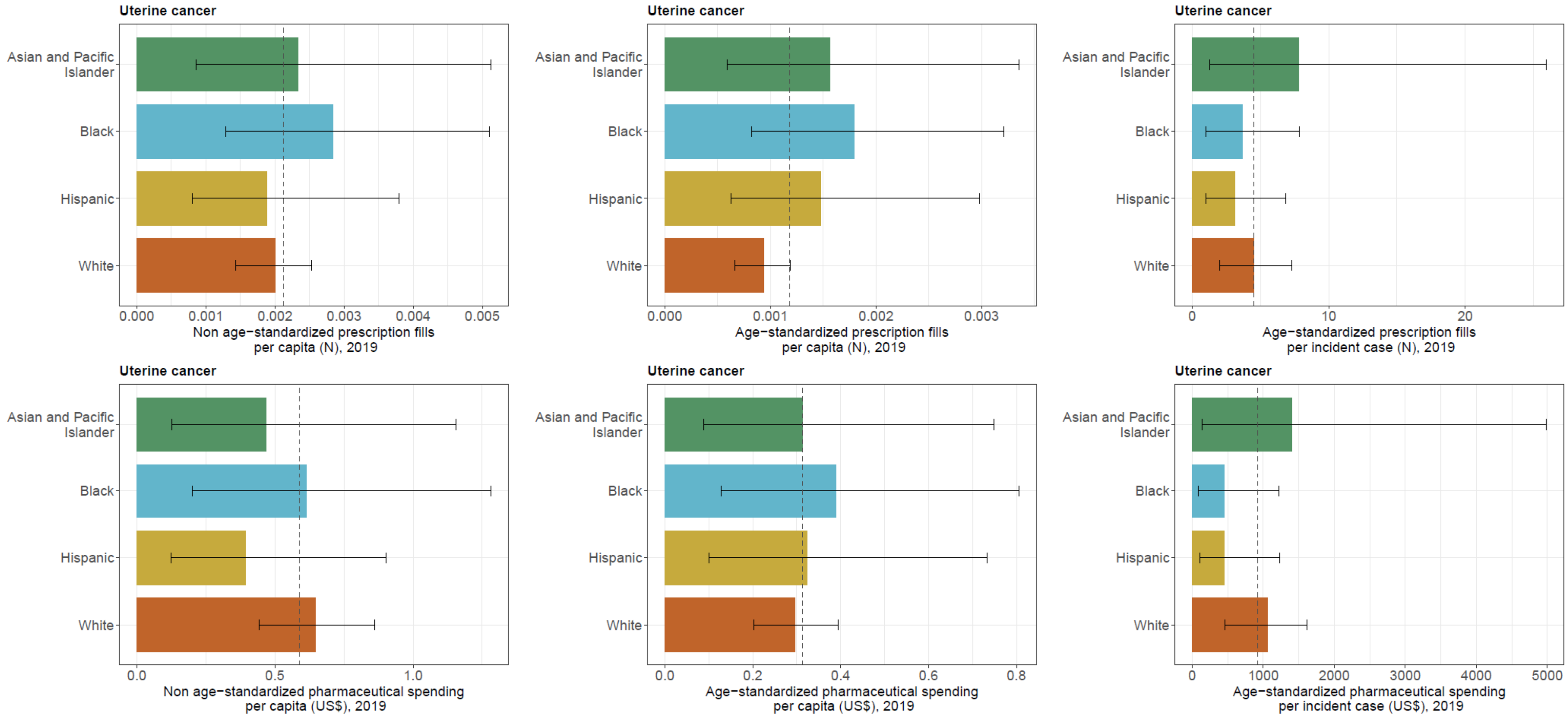

© 2025 Sahu M et al. JAMA Health Forum.

(34) Falls

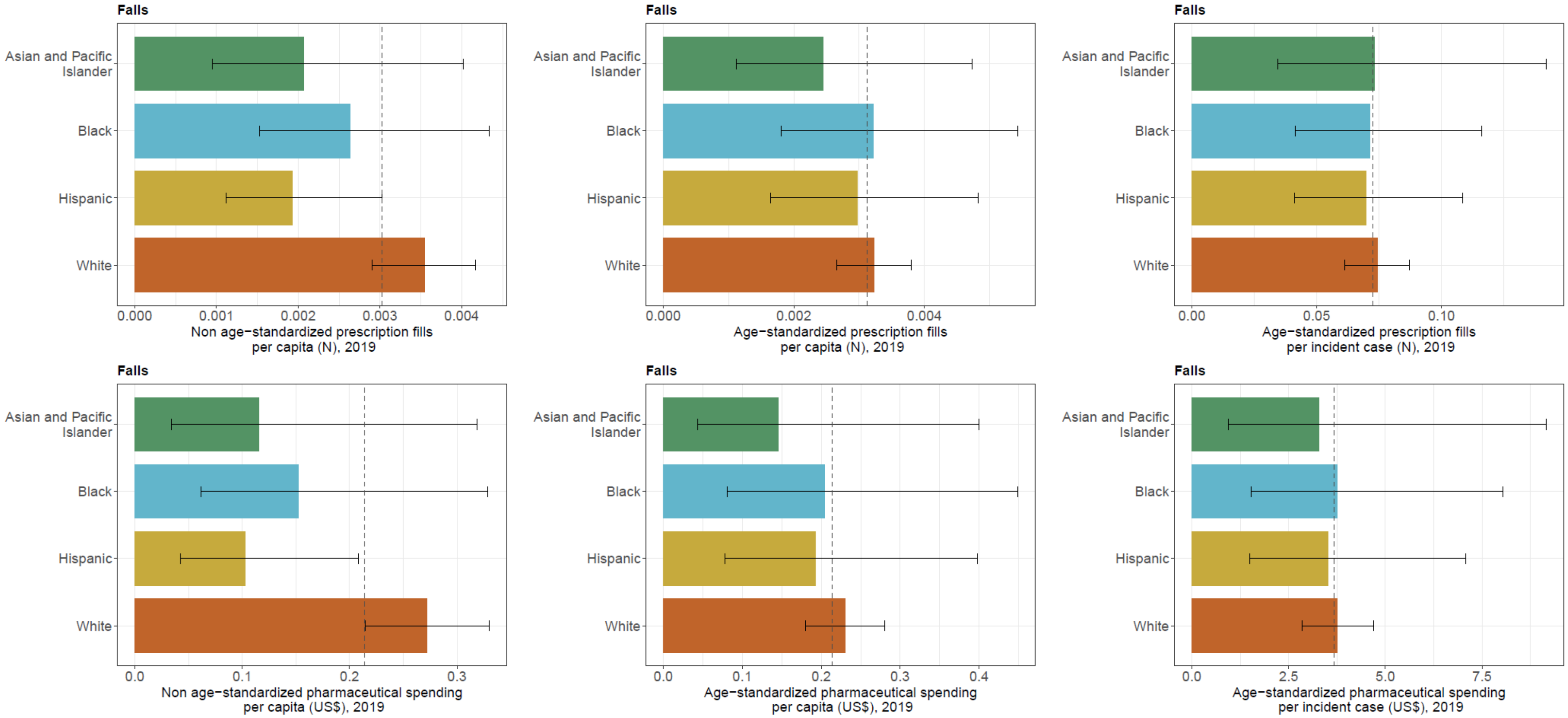

© 2025 Sahu M et al. JAMA Health Forum.

(35) Cervical cancer

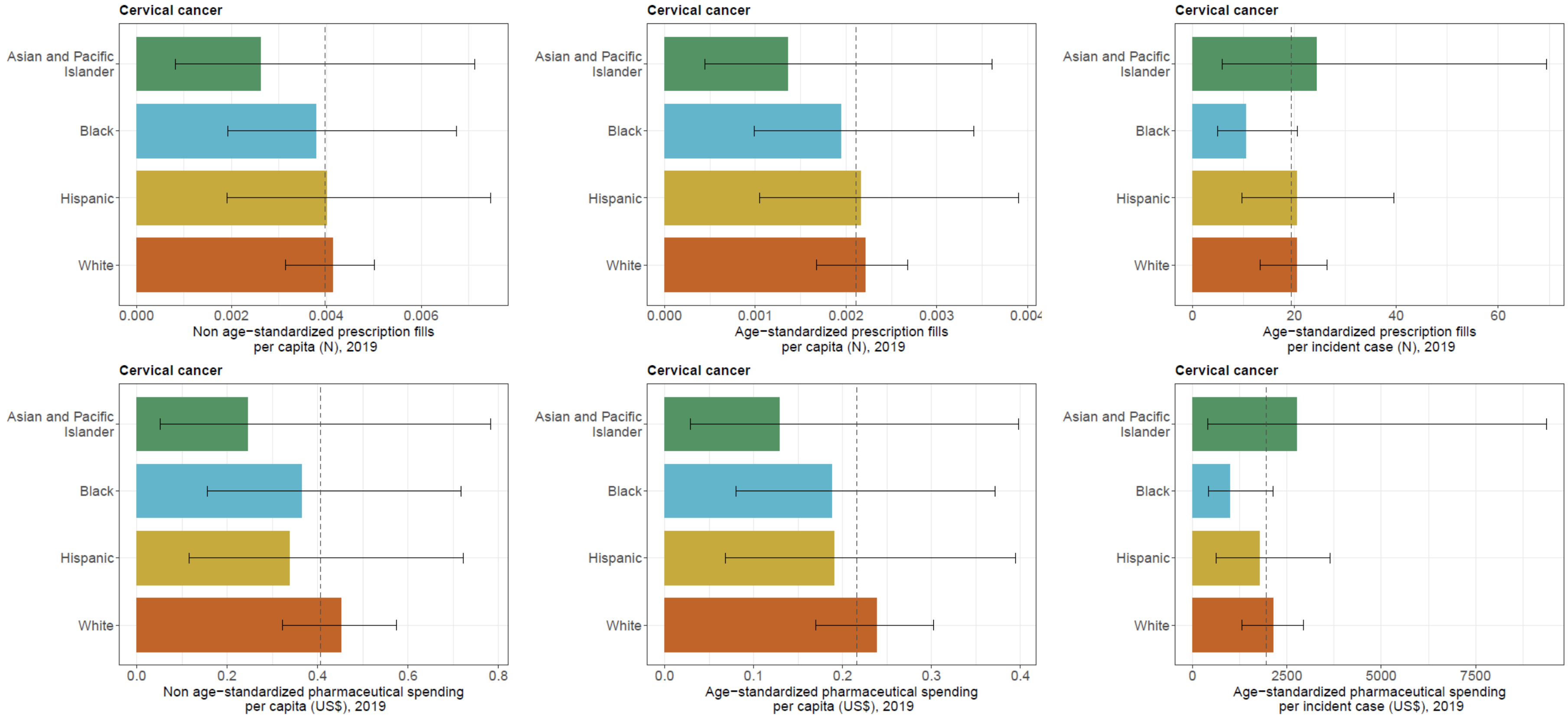

© 2025 Sahu M et al. JAMA Health Forum.

(36) Bladder cancer

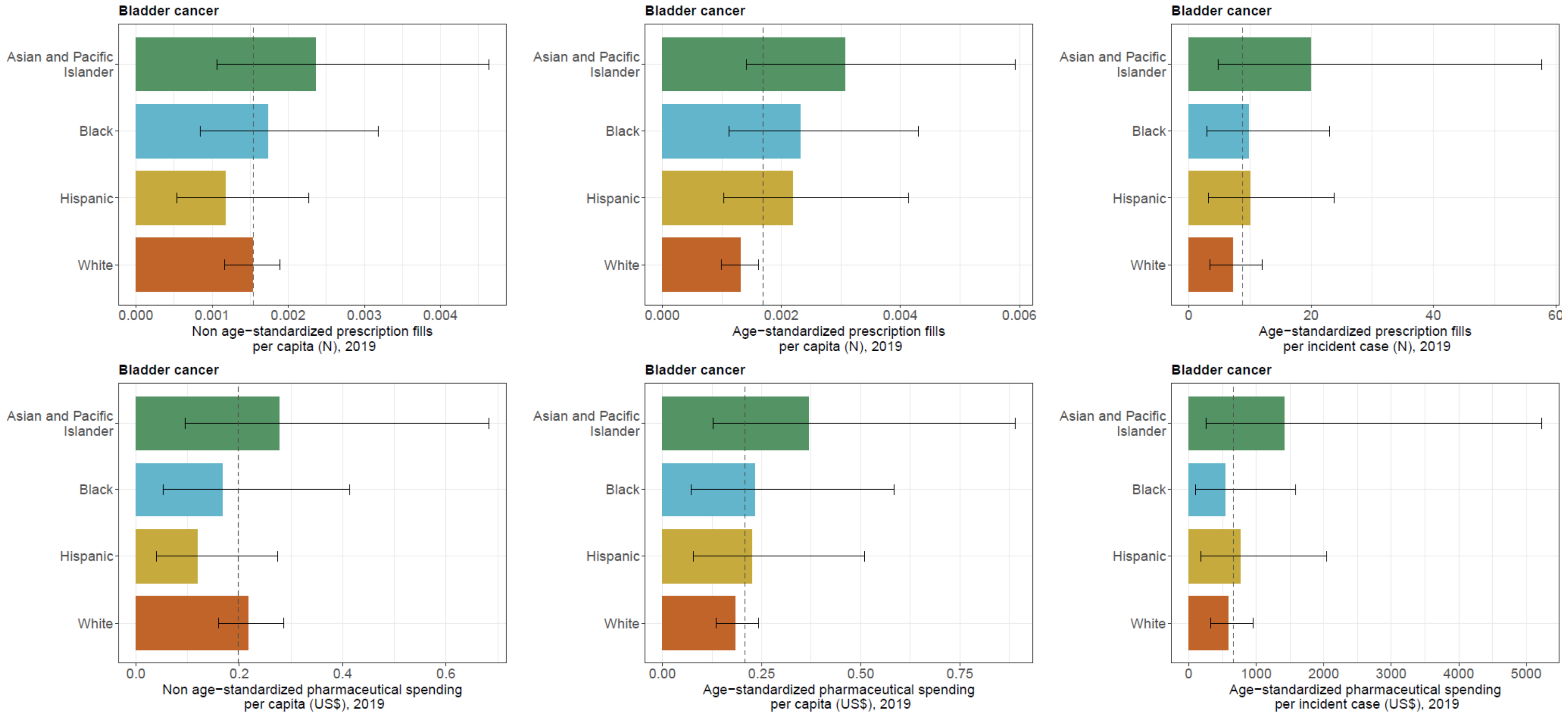

© 2025 Sahu M et al. JAMA Health Forum.

(37) Tuberculosis

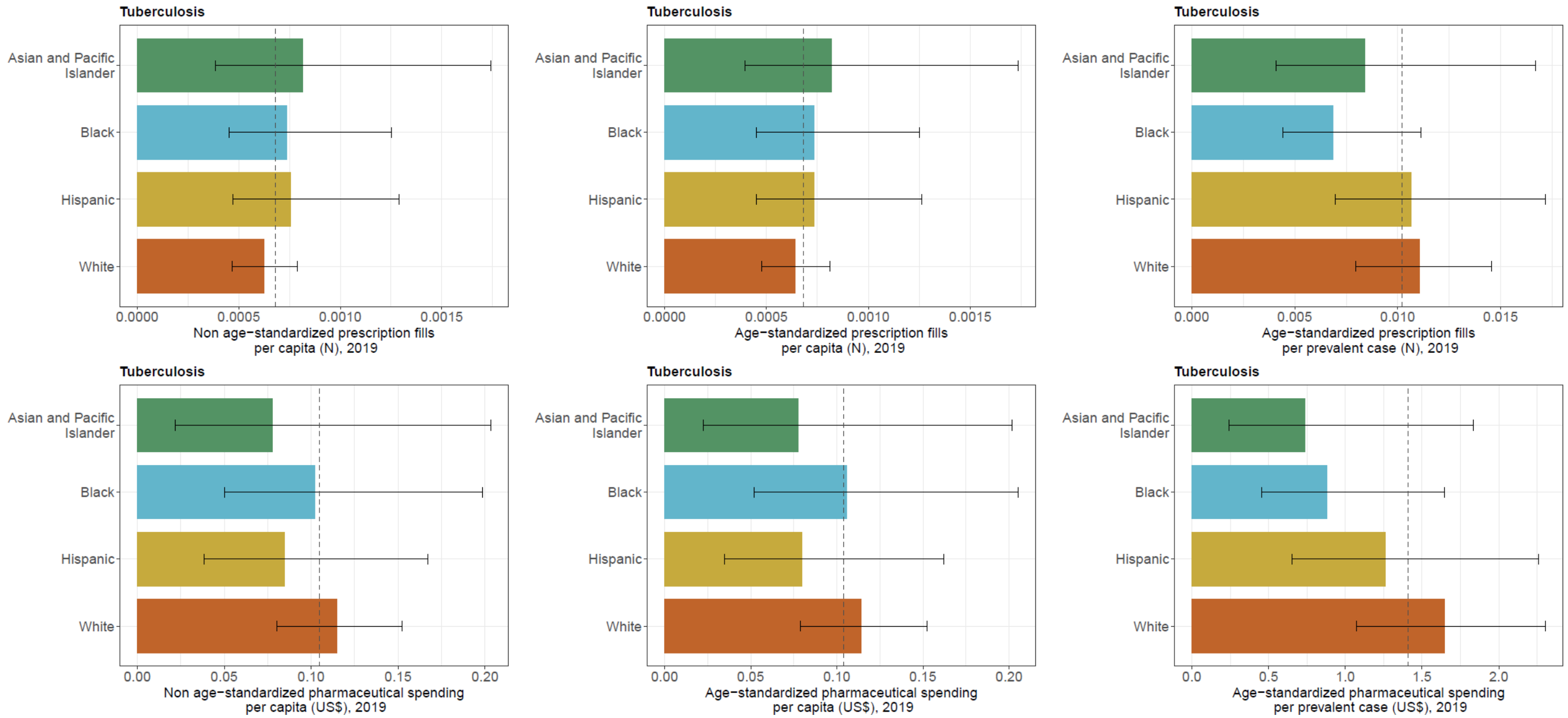

© 2025 Sahu M et al. JAMA Health Forum.

(38) Stomach cancer

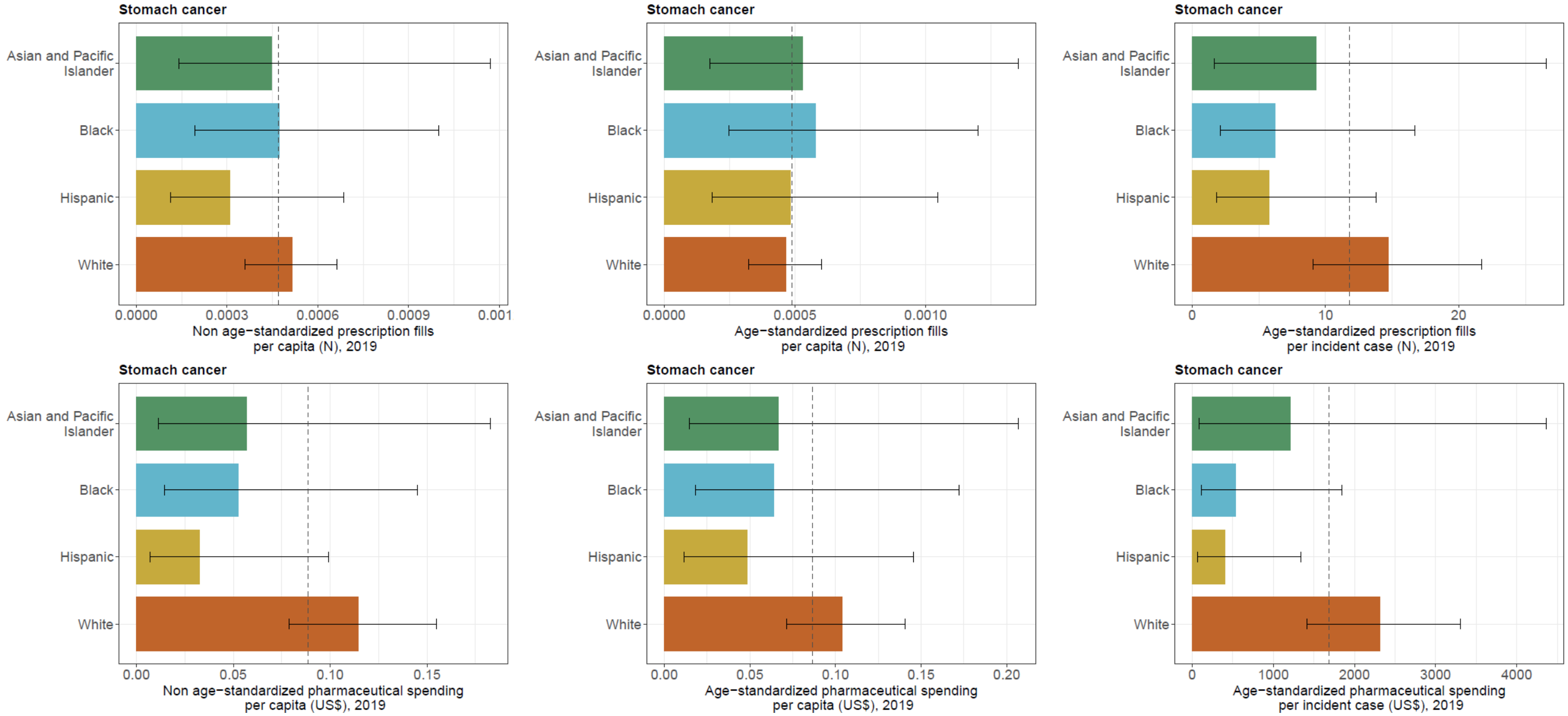

© 2025 Sahu M et al. JAMA Health Forum.

(39) Endocarditis

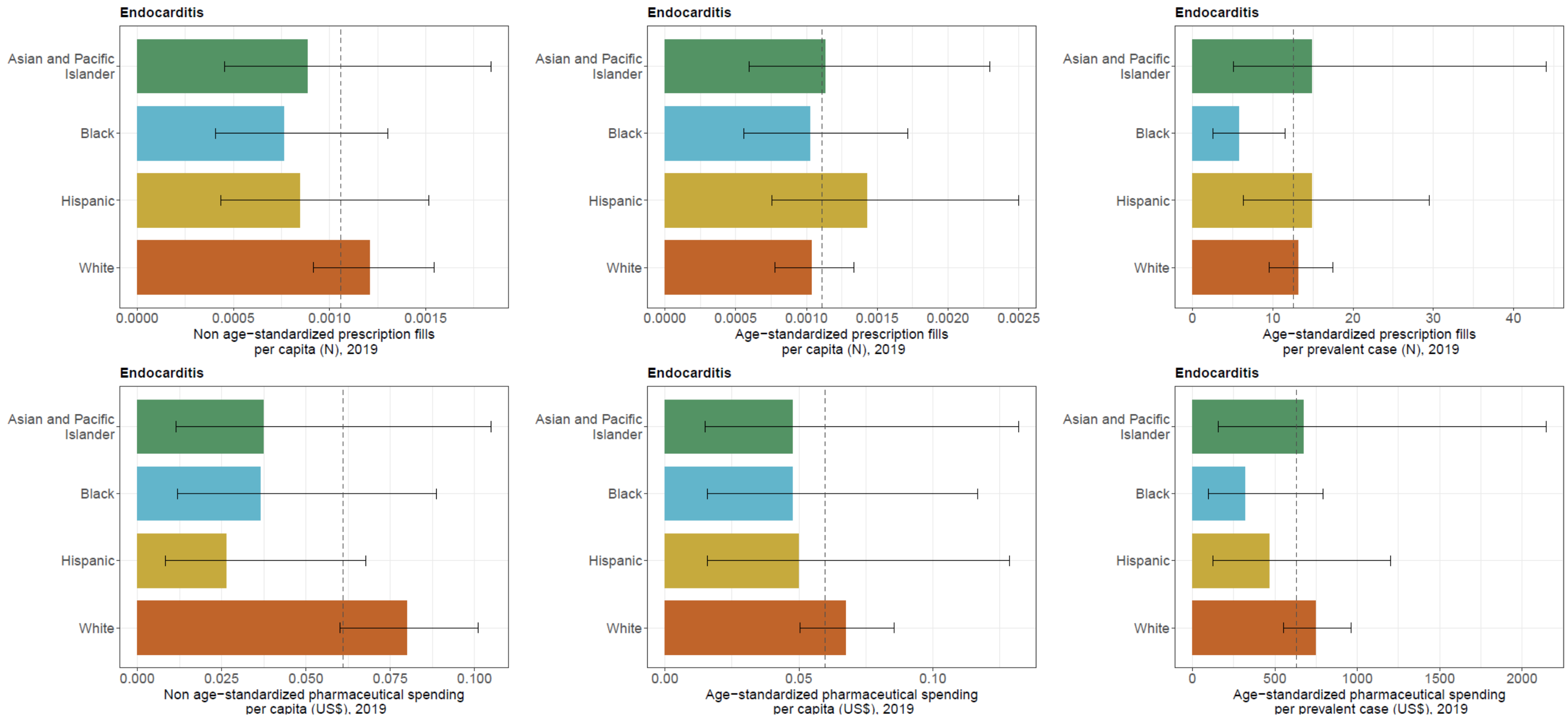

© 2025 Sahu M et al. JAMA Health Forum.

(40) Esophageal cancer

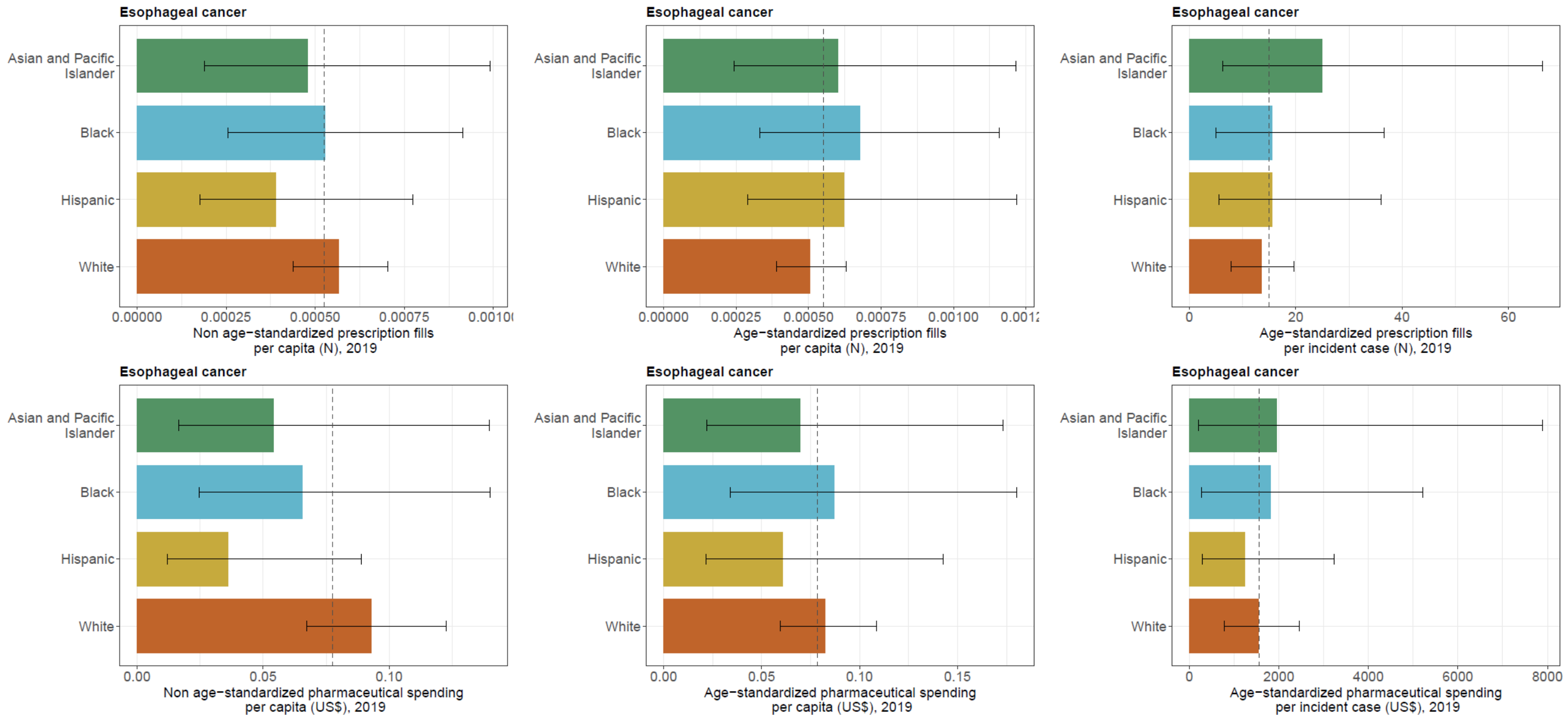

© 2025 Sahu M et al. JAMA Health Forum.

(41) Lip and oral cavity cancer

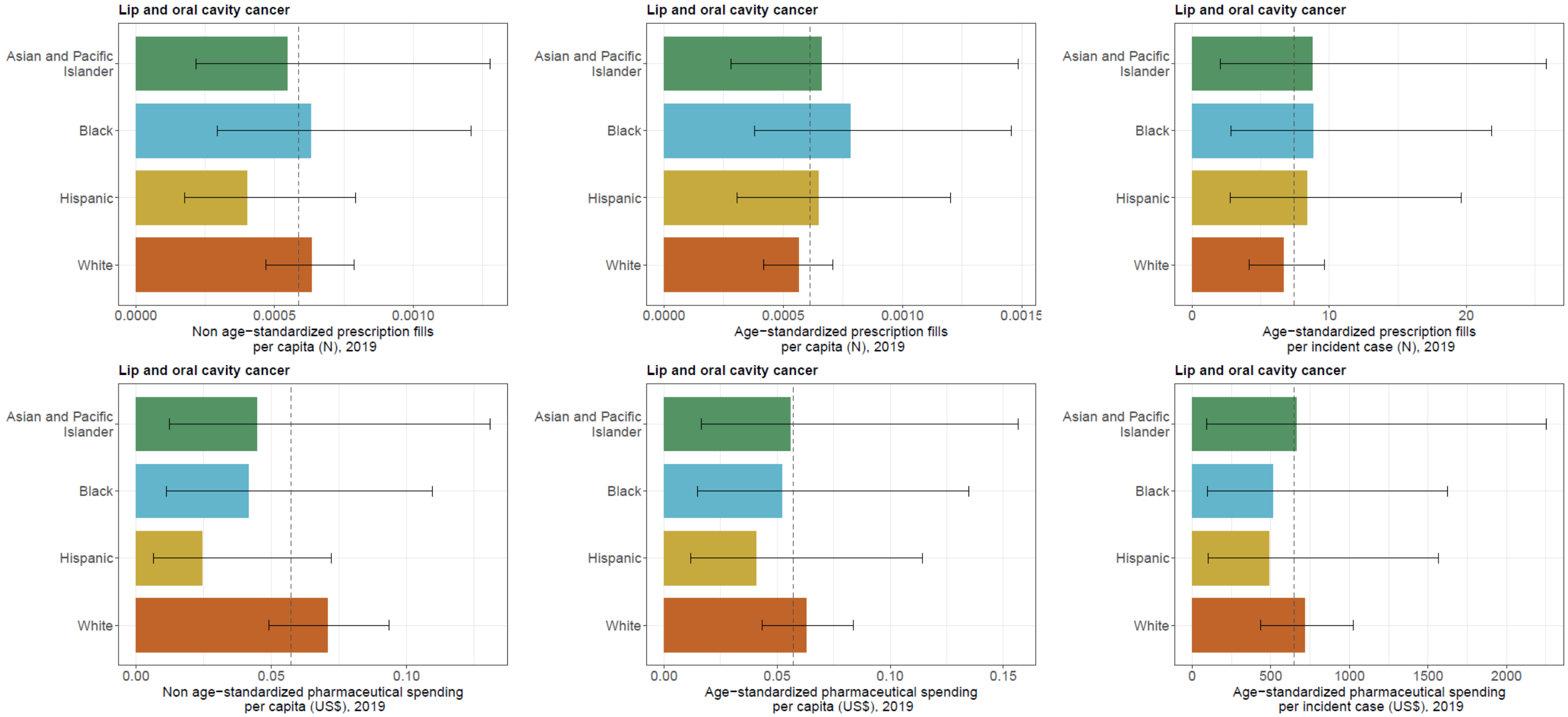

© 2025 Sahu M et al. JAMA Health Forum.

(42) Hodgkin lymphoma

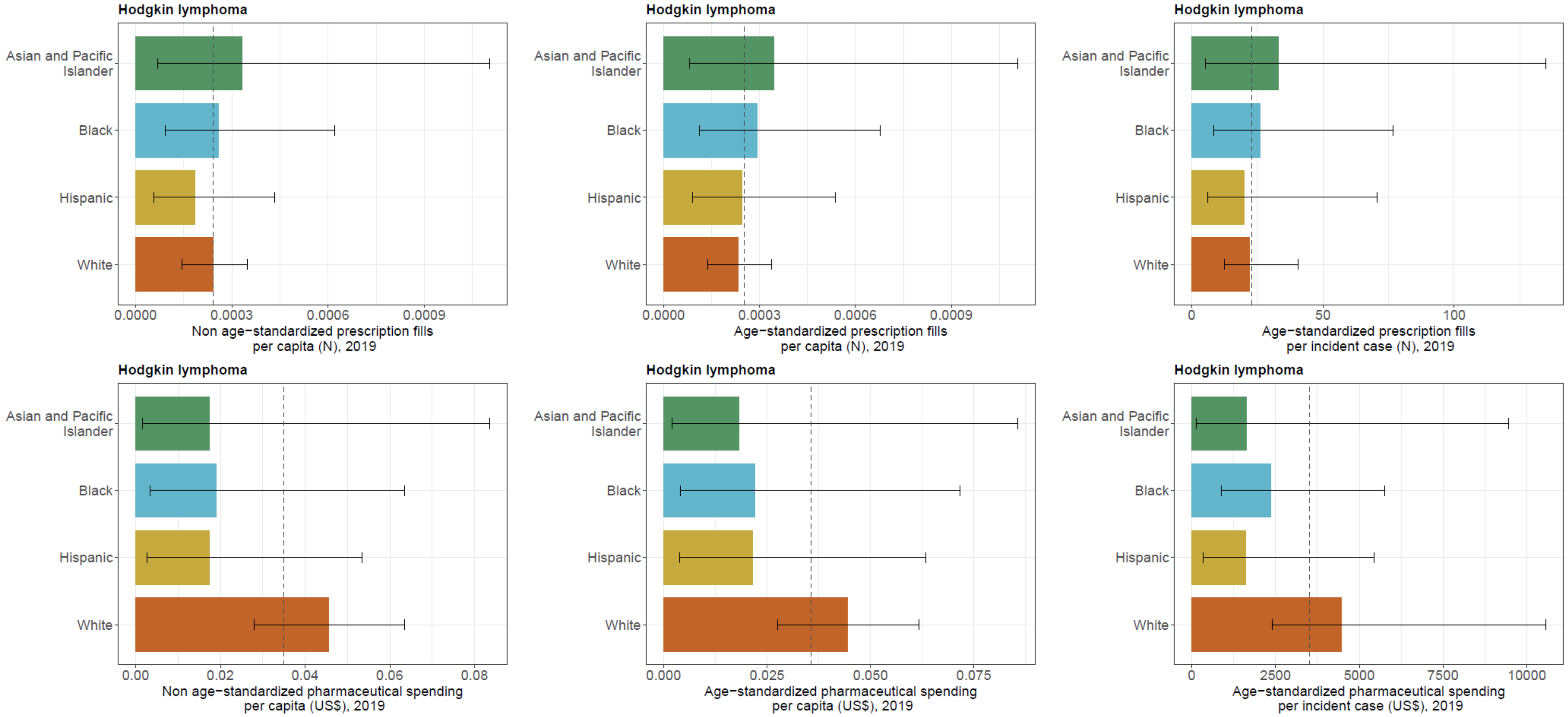

© 2025 Sahu M et al. JAMA Health Forum.

(43) Other pharynx cancer

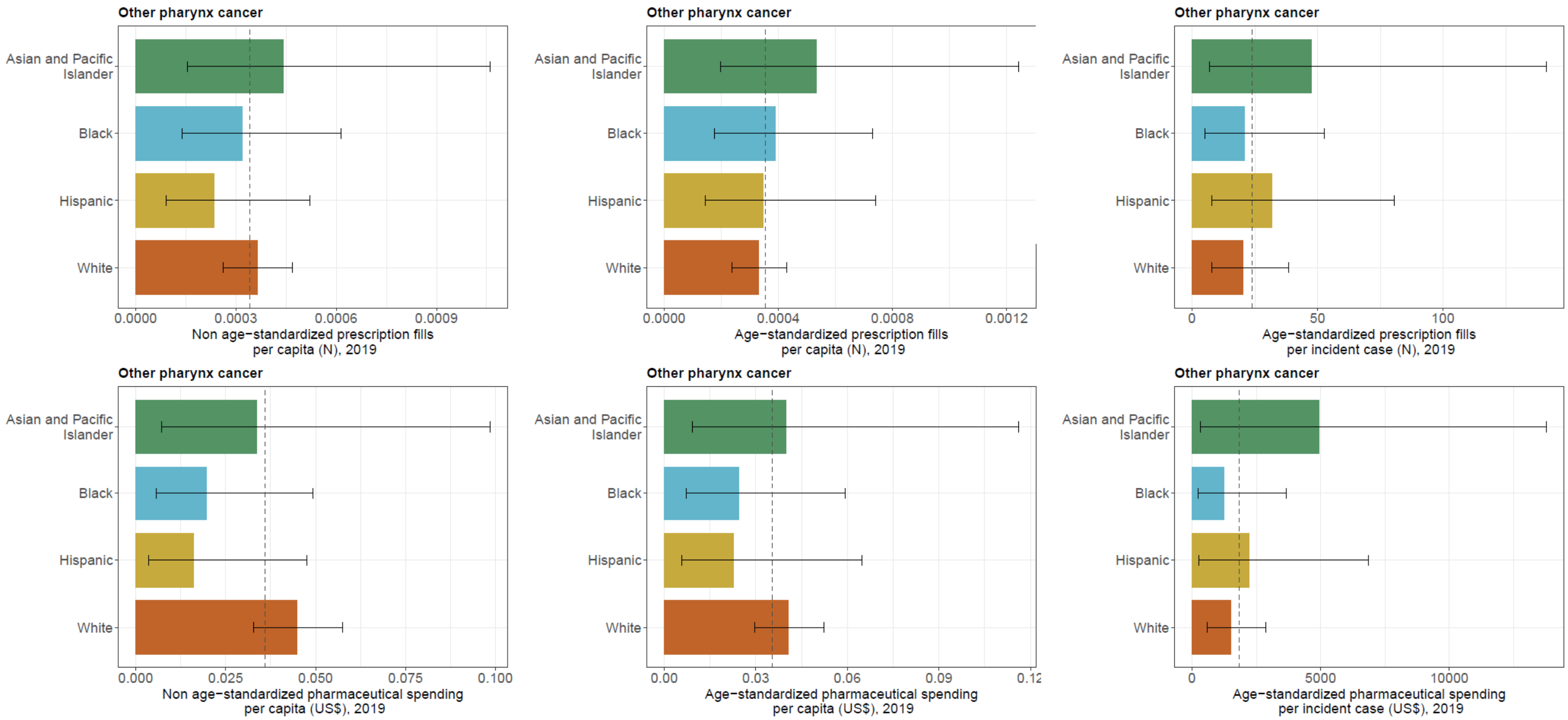

© 2025 Sahu M et al. JAMA Health Forum.

(44) Transport injuries

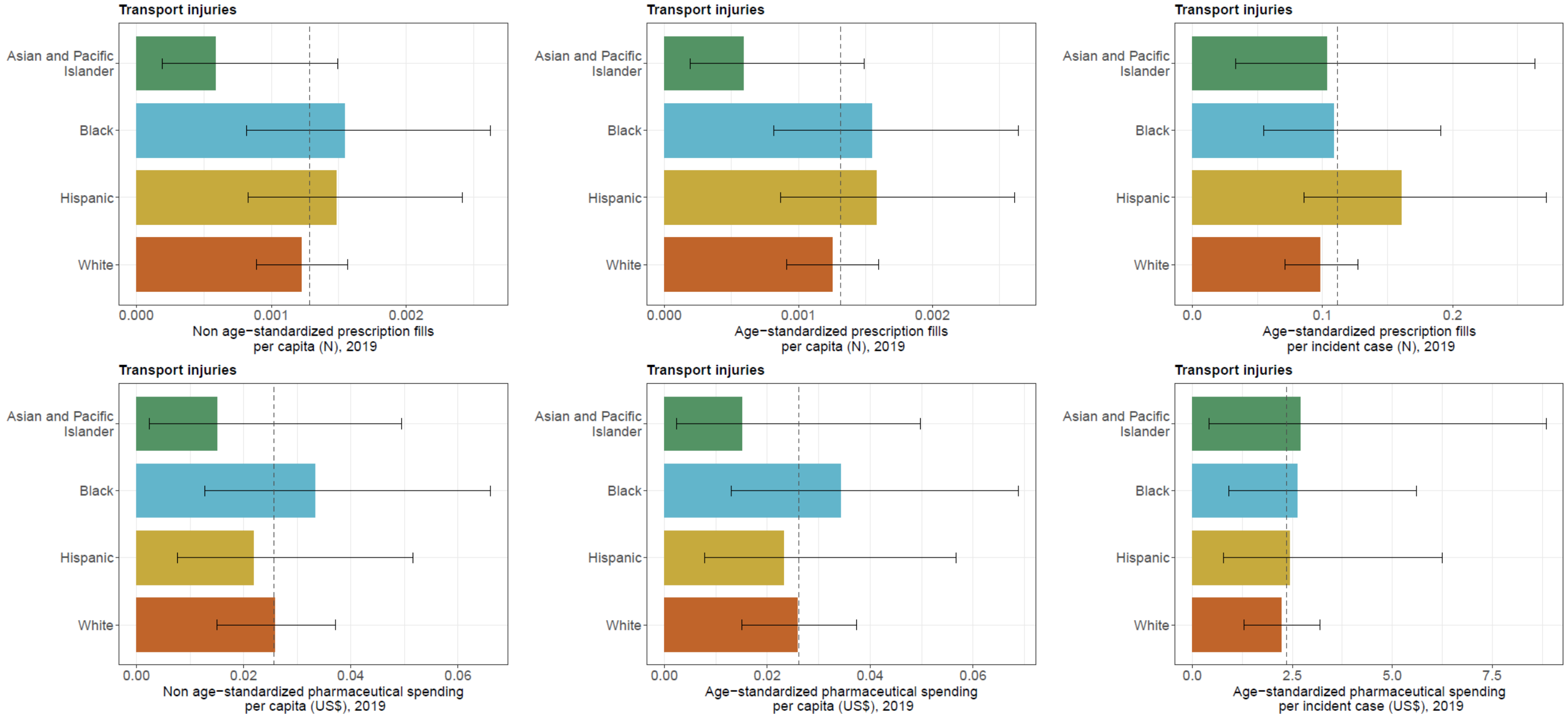

© 2025 Sahu M et al. JAMA Health Forum.

(45) Exposure to mechanical forces

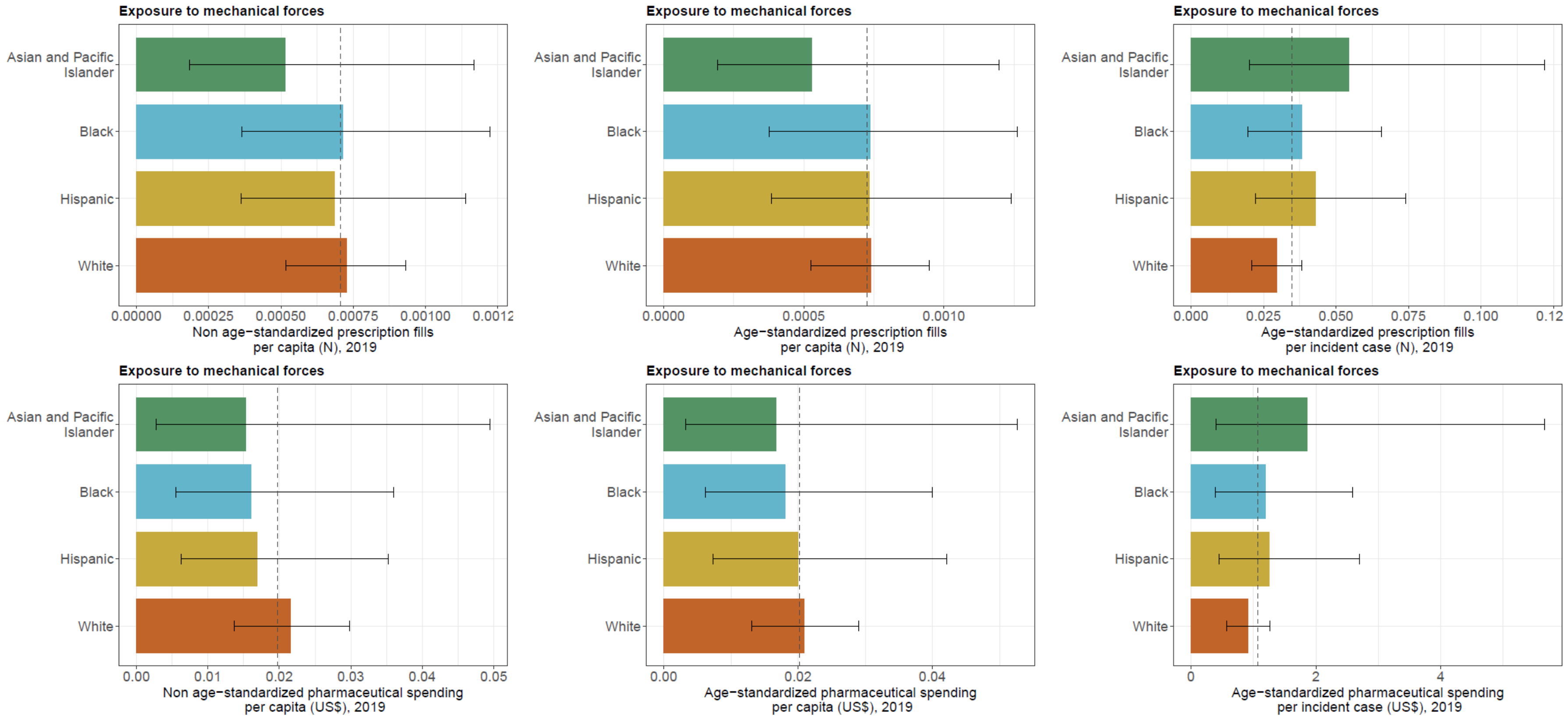

© 2025 Sahu M et al. JAMA Health Forum.

(46) Vascular intestinal disorders

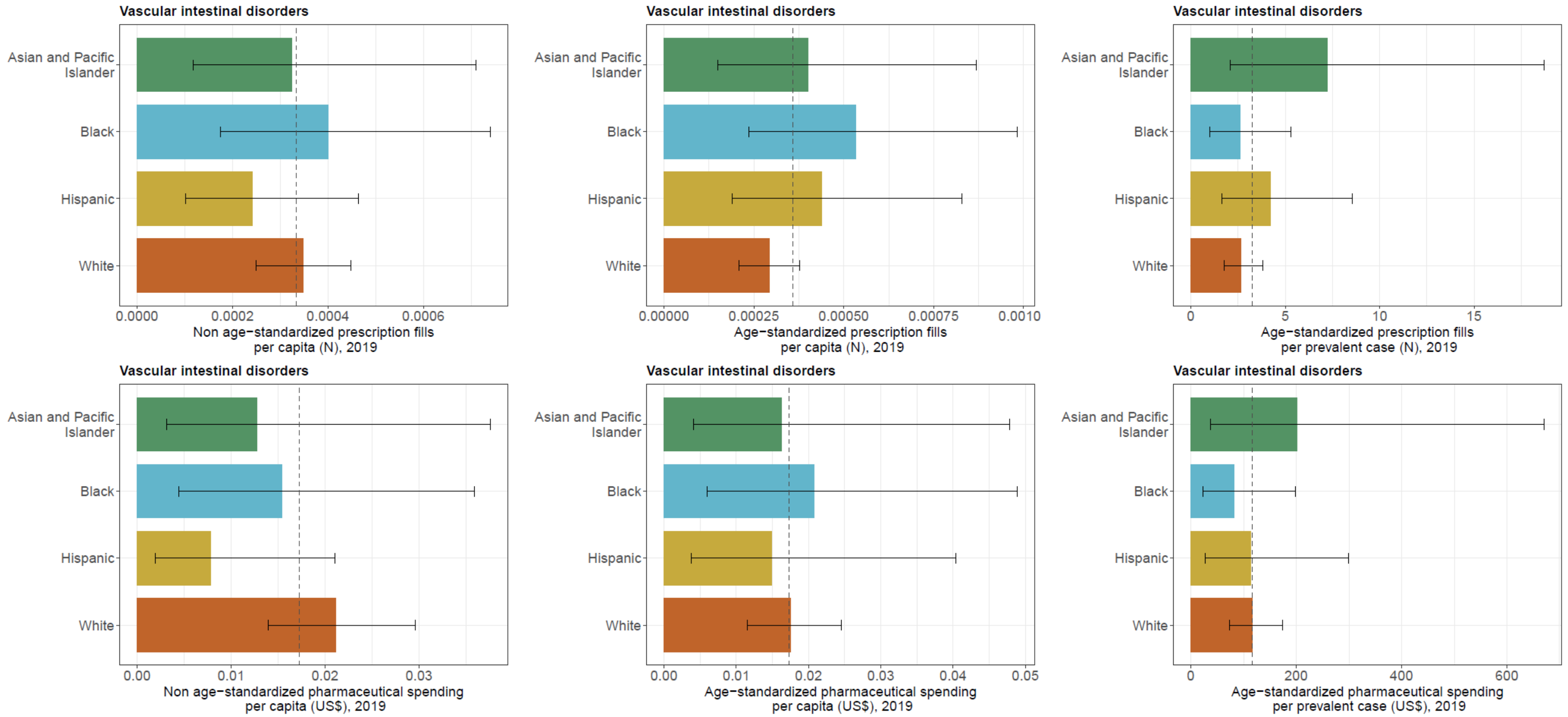

© 2025 Sahu M et al. JAMA Health Forum.

(47) Larynx cancer

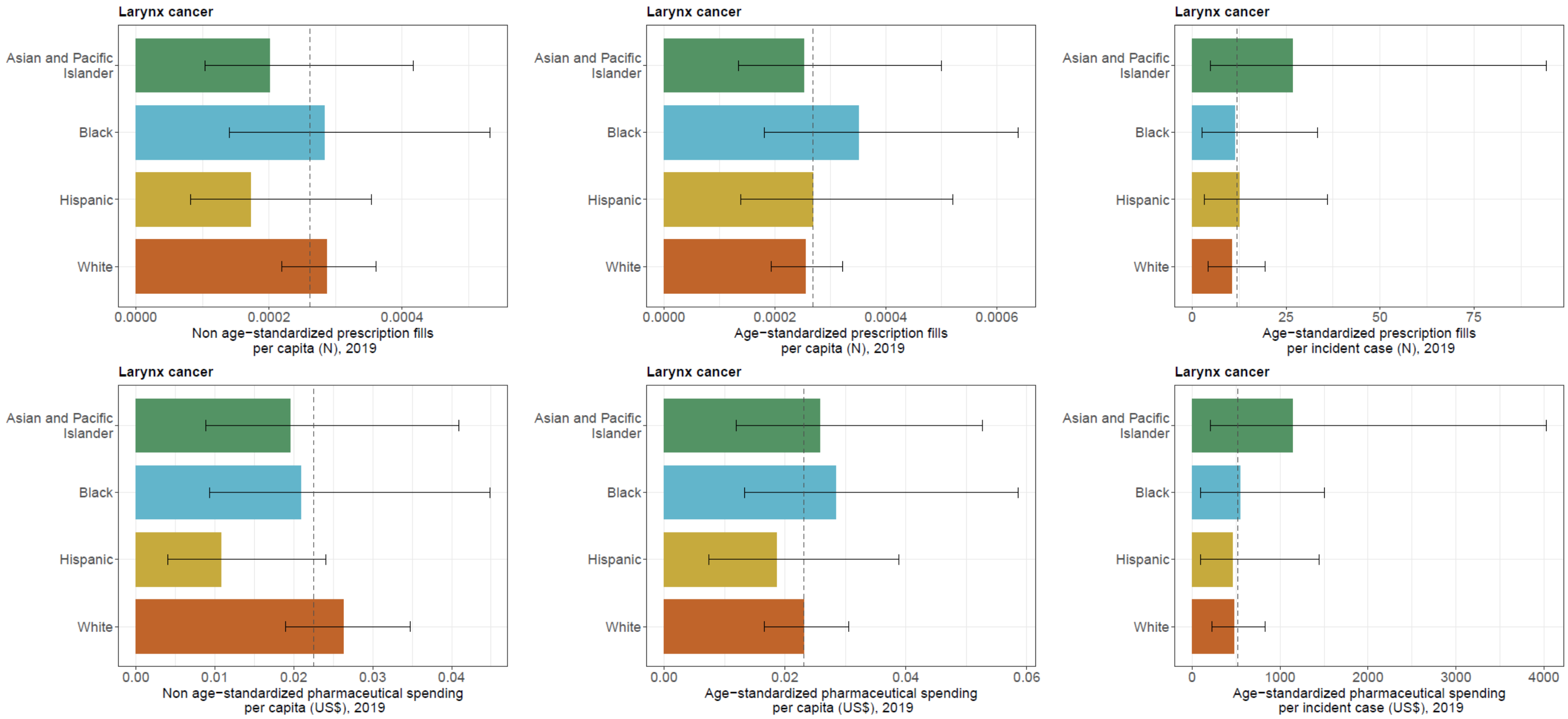

© 2025 Sahu M et al. JAMA Health Forum.

(48) Gallbladder and biliary tract cancer

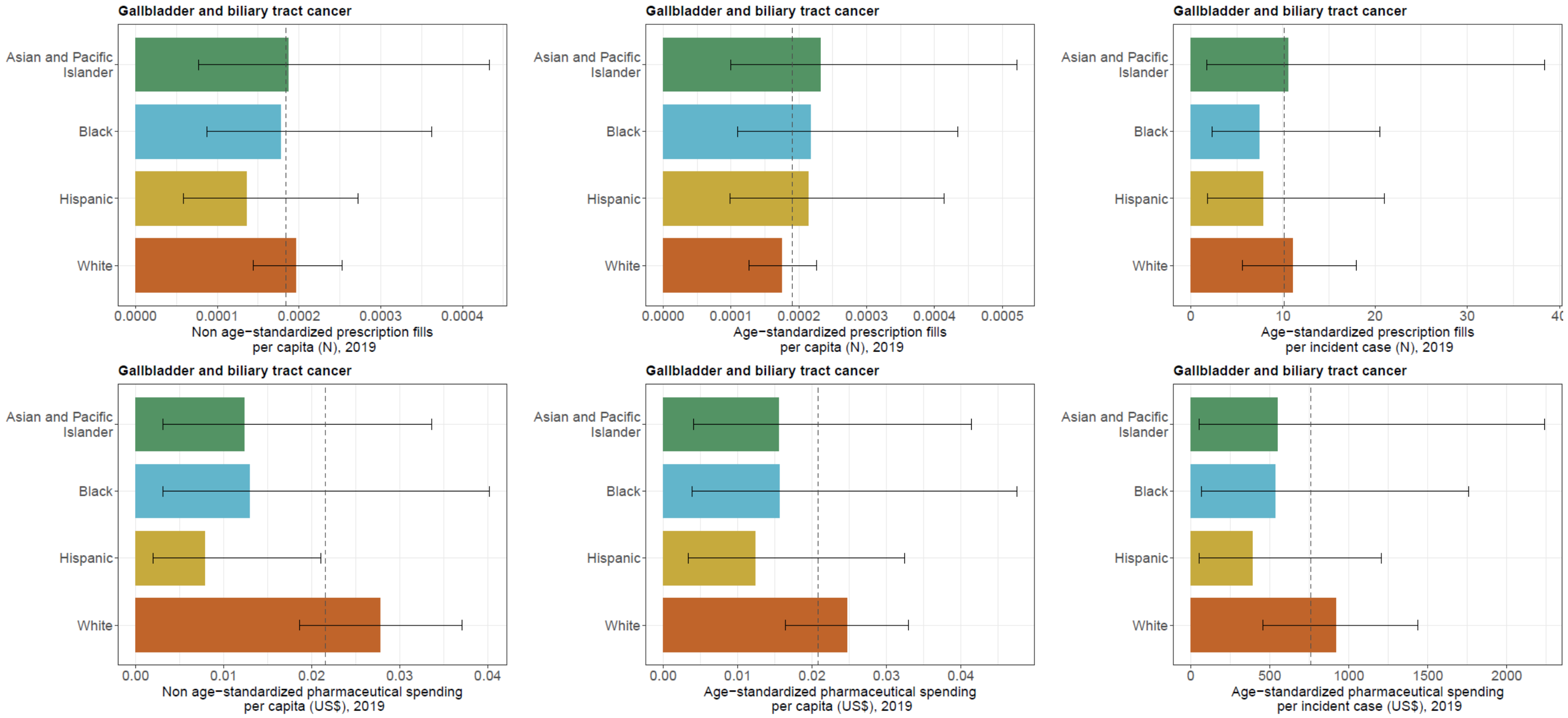

© 2025 Sahu M et al. JAMA Health Forum.

(49) Mesothelioma

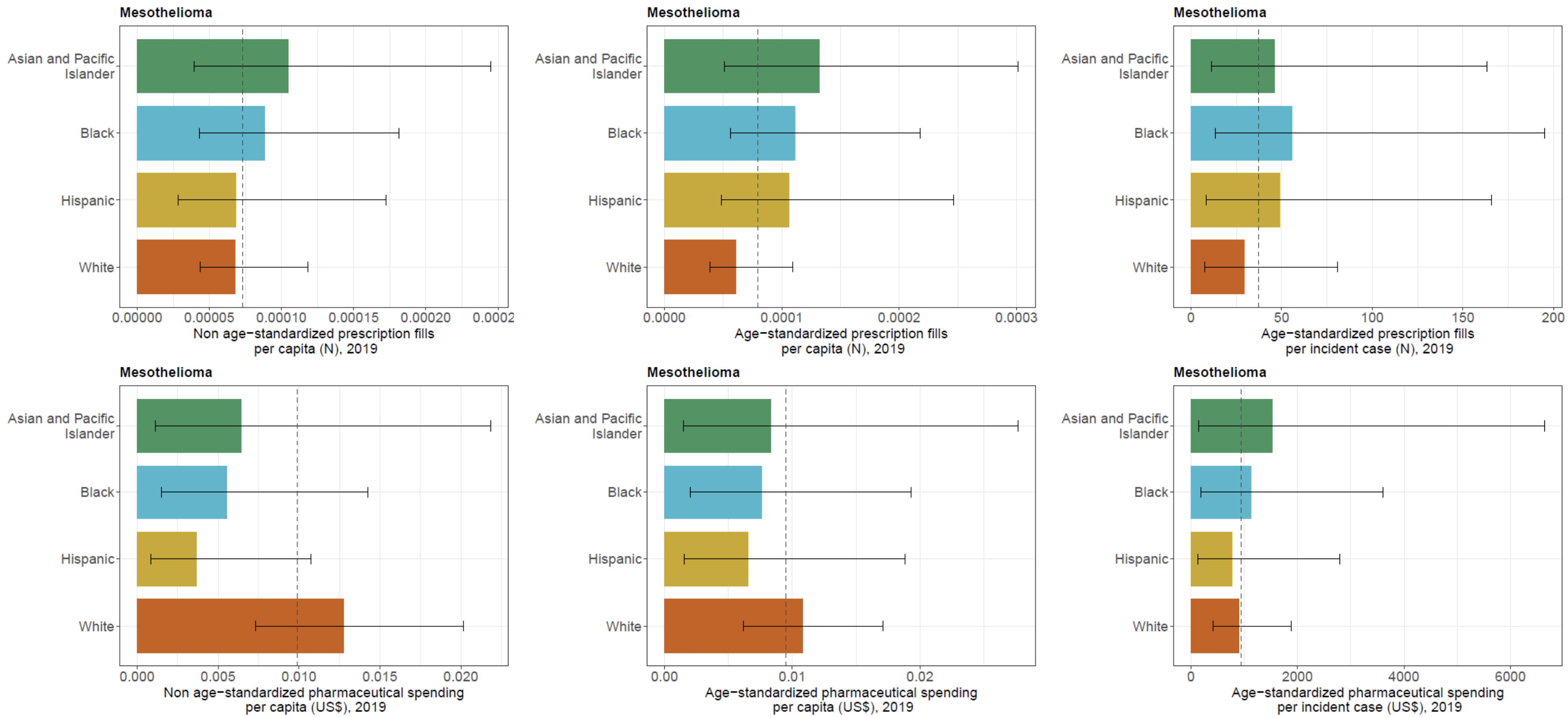

© 2025 Sahu M et al. JAMA Health Forum.

(50) Neonatal preterm birth

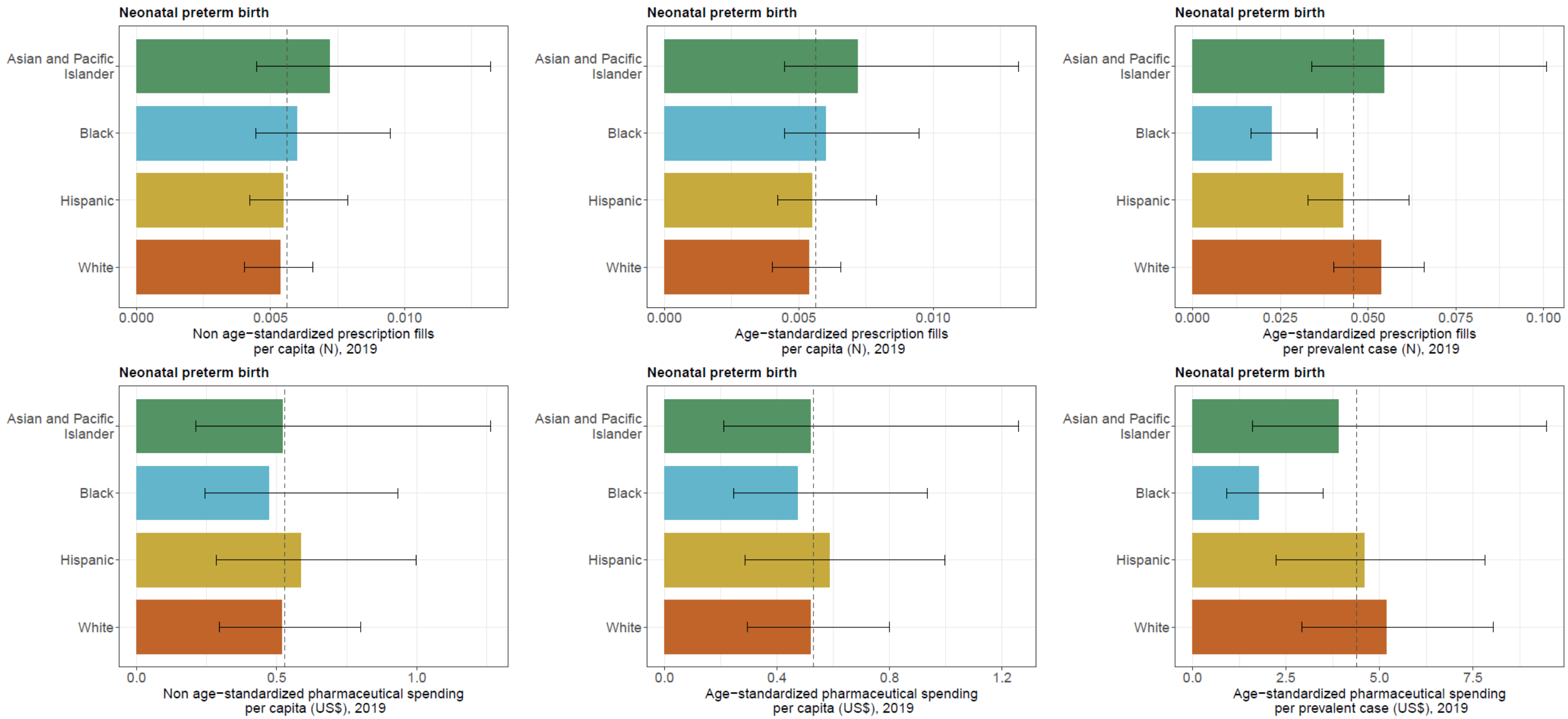

© 2025 Sahu M et al. JAMA Health Forum.

(51) Nasopharynx cancer

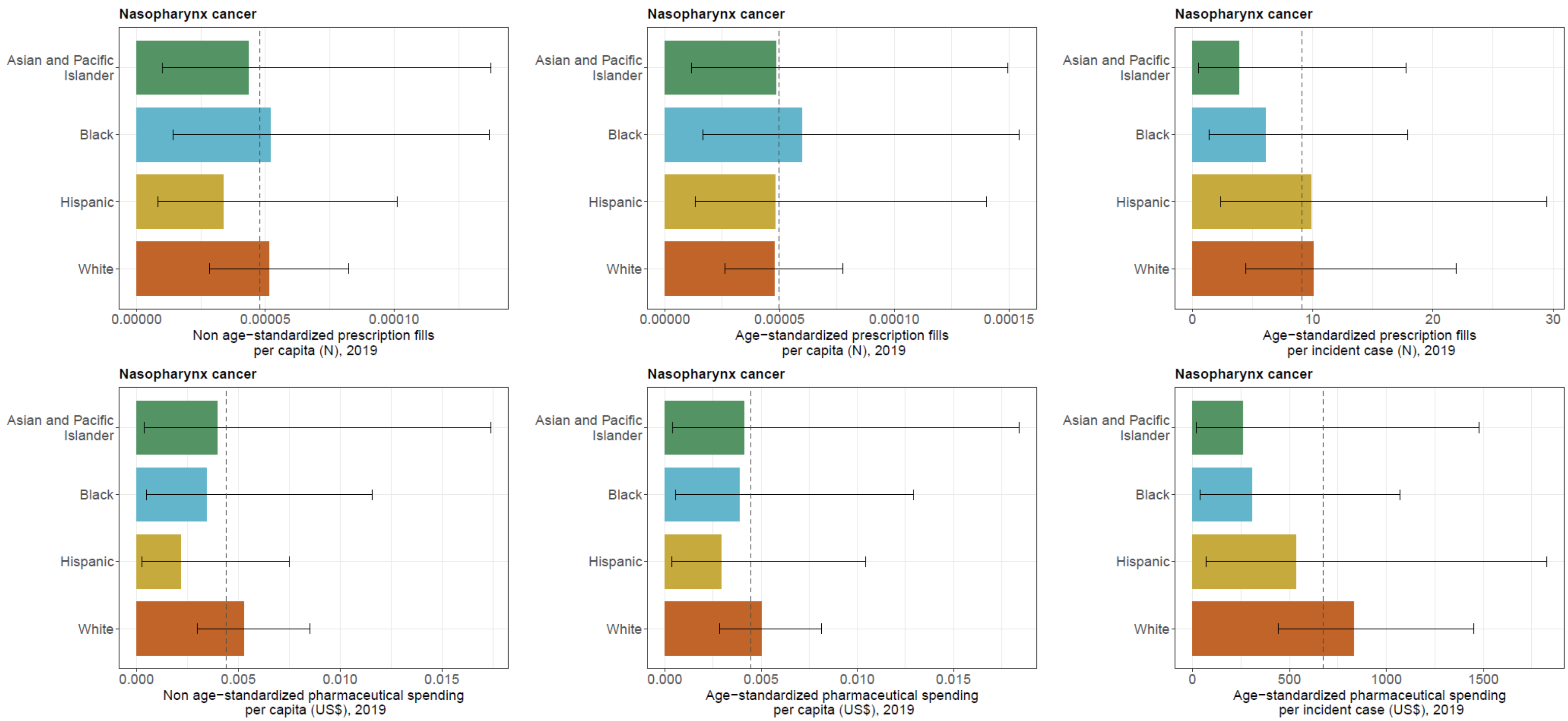

© 2025 Sahu M et al. JAMA Health Forum.

(52) Self-harm

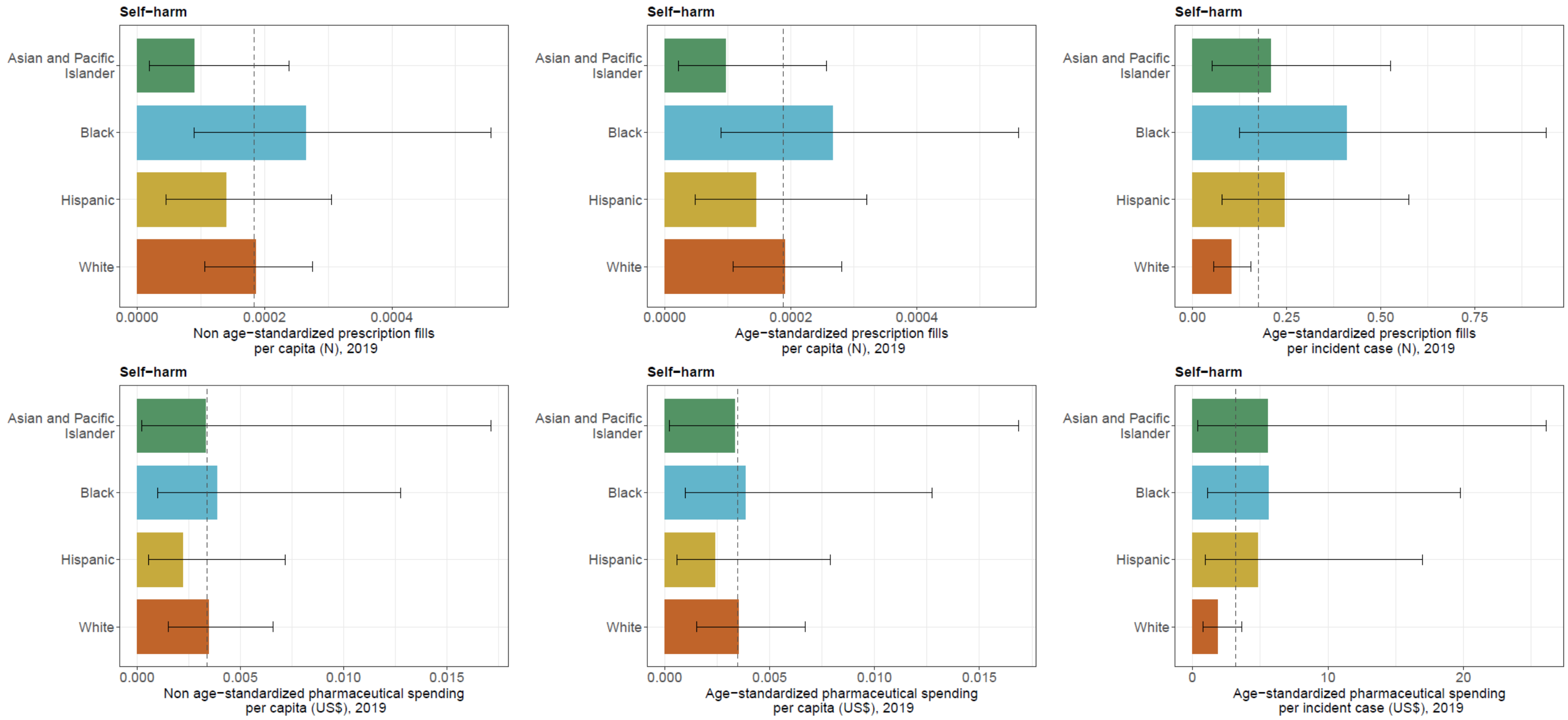

© 2025 Sahu M et al. JAMA Health Forum.

D. eFigure 2: *Decomposition of pharmaceutical spending per capita, for 52 conditions*

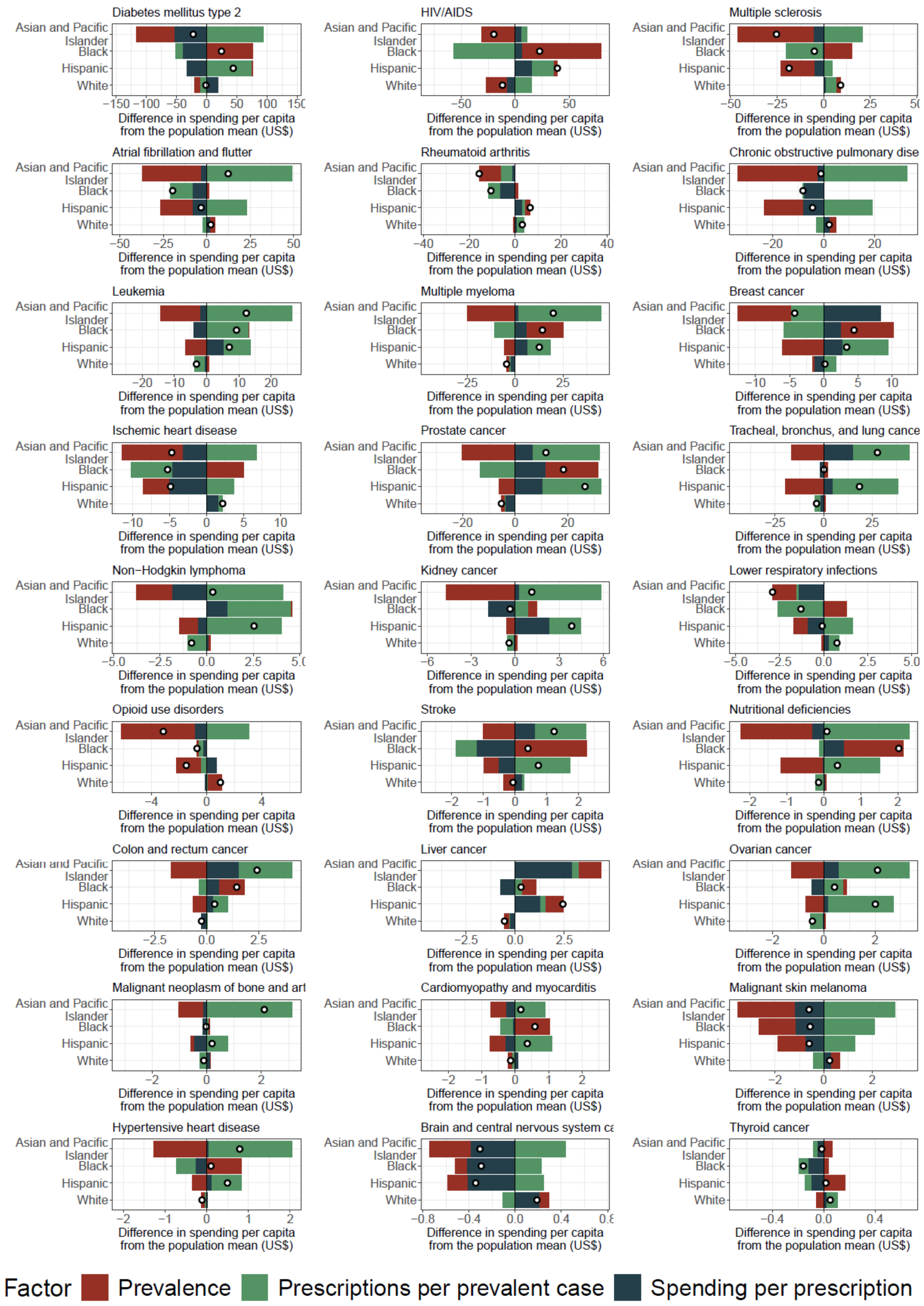

© 2025 Sahu M et al. JAMA Health Forum.

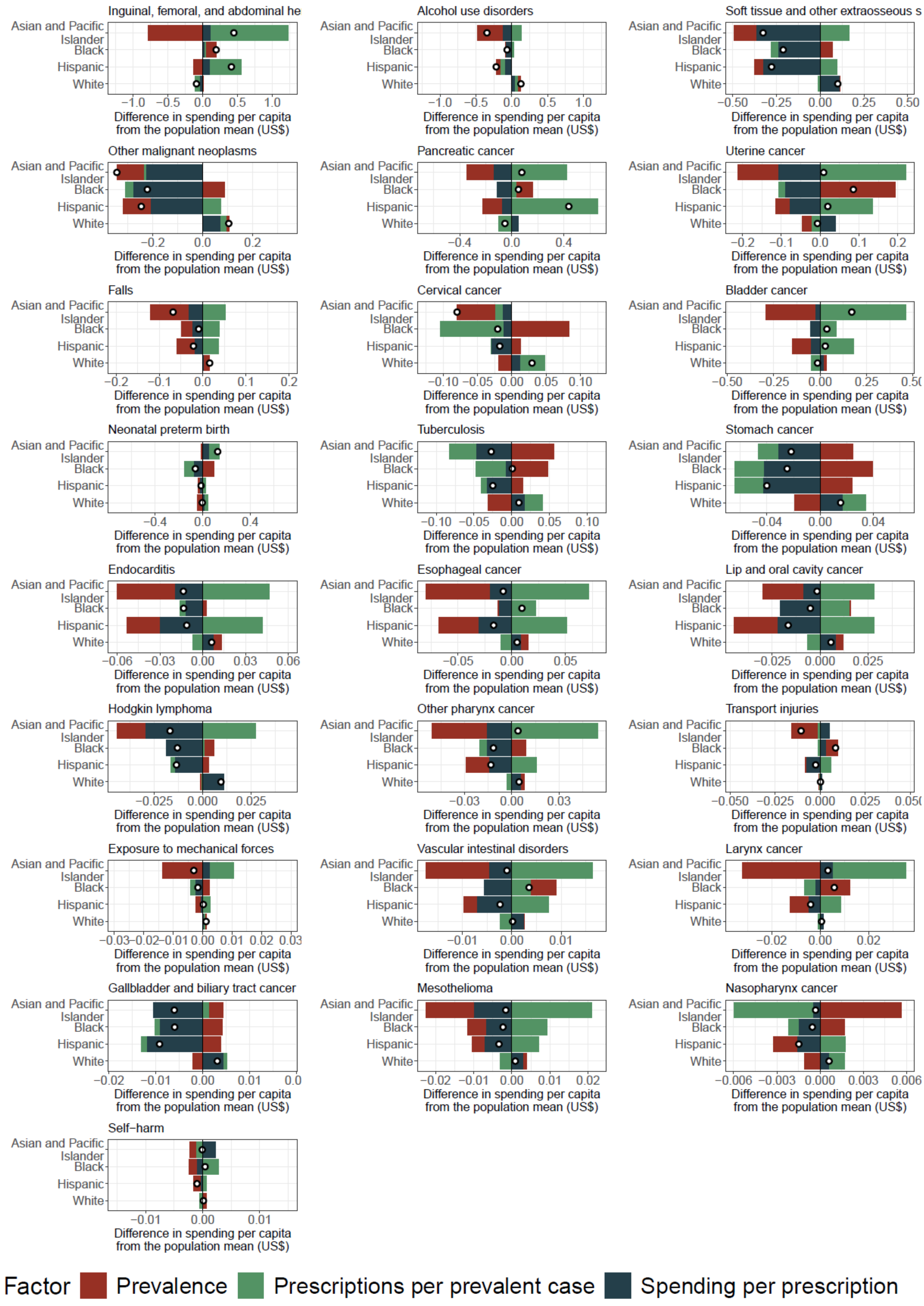

© 2025 Sahu M et al. JAMA Health Forum.

eAppendix 4. STATE-LEVEL SUPPLEMENTAL RESULTS

A. eTable : *Pharmaceutical spending and utilization per capita by race and ethnicity (age-standardized)*

For each US state and the District of Columbia, we present the total per-capita pharmaceutical spending and utilization by race/ethnicity. States are ranked by total (national) retail pharmaceutical spending. All groups other than Hispanic are non-Hispanic ethnicity. The mean values across 100 draws are presented in the top row, with the 95% Uncertainty Interval below.

State	Rank (all-population per-capita drug spending, age-std)	Rank (all-population per-capita prescription fills, age-std)	Spending per capita (US$)				Prescription fills per capita (n)			
			Asian and Pacific Islander	Black	Hispanic	White	Asian and Pacific Islander	Black	Hispanic	White
			381.2	603.1	574.3	914.6	3.6	4.9	4.6	6.9
Alaska	44	48	(333.7, 451)	(519.6, 706.7)	(481.4, 698.2)	(893.9, 929.8)	(3.4, 3.9)	(4.5, 5.3)	(4.1, 5)	(6.6, 7.3)
			516.1	1081.7	605	1363.4	4.8	9.6	5.5	11.9
Alabama	8	2	(406.4, 752.3)	(1003.5, 1169.3)	(504.9, 792.2)	(1331.4, 1392.5)	(4.3, 5.4)	(8.8, 10.2)	(5, 5.8)	(11.4, 12.4)
			461.3	928.5	3231.8	982.9	5.4	8.7	26.2	9.4
Arkansas	26	10	(358, 650.8)	(847.9, 1029.2)	(2888.3, 3581.5)	(955, 1005)	(4.9, 6)	(8, 9.3)	(23.6, 28.4)	(9, 9.8)
			725.6	848.4	767.8	976.1	5.6	7	6.4	7.3
Arizona	35	38	(633.5, 925)	(746.6, 997.2)	(705.6, 850.7)	(948.2, 1005.5)	(5, 6.1)	(6.5, 7.4)	(5.9, 6.9)	(7, 7.6)
			734.4	931.9	891.5	1103.9	5.2	6.3	6.2	6.8
California	36	47	(672, 836.7)	(850.6, 1057)	(836.6, 965.7)	(1048.4, 1159)	(4.6, 5.6)	(5.9, 6.7)	(5.8, 6.5)	(6.5, 7.1)
			480.6	599	779.5	796.3	3.8	5.1	6.2	6
Colorado	45	50	(408.9, 635.6)	(520.6, 687.6)	(715.8, 865.2)	(776.8, 813.1)	(3.4, 4.1)	(4.8, 5.4)	(5.7, 6.6)	(5.7, 6.2)
			1451.6	1181.7	1405.5	1405.3	10	8.3	10.2	9.1
Connecticut	5	11	(1260, 1780.5)	(1086.9, 1306.8)	(1278.7, 1555.9)	(1370.5, 1434.3)	(9.1, 11)	(7.7, 8.7)	(9.4, 11)	(8.7, 9.4)
			1250	1512.5	1420.3	2569	10.7	11	9.8	17.7
District of Columbia	1	1	(919.8, 1838.6)	(1379.8, 1671.3)	(1174.8, 1868.4)	(2350.5, 2735.9)	(9.1, 12.3)	(10.4, 11.8)	(8.9, 10.7)	(16.5, 18.8)

© 2025 Sahu M et al. JAMA Health Forum.

			488.3	1468.7	714	1413.3	3.4	9.9	5.5	8.4
Delaware	4	16	(409.4, 628.7)	(1355.7, 1597)	(627, 867.9)	(1378.4, 1451.7)	(3.1, 3.7)	(9.1, 10.5)	(5.2, 5.7)	(8, 8.8)
			1074	943.9	1105.4	1108.6	7.4	7	7.9	7.4
Florida	15	24	(953, 1345)	(859.1, 1048.7)	(1026.9, 1213.7)	(1064.8, 1151.8)	(6.6, 8.1)	(6.4, 7.3)	(7.3, 8.4)	(7.1, 7.6)
			1029.3	926.1	1674.4	1147.4	7.6	7.4	12	8.5
Georgia	27	30	(905.5, 1311.1)	(847.7, 1006.8)	(1483.7, 1898.6)	(1093.8, 1197.1)	(6.9, 8.4)	(6.9, 7.9)	(10.8, 13.1)	(8.1, 8.9)
			877.9	1633.7	2166.6	2316.2	6.1	11.8	14.6	13.6
Hawaii	3	12	(791, 955.5)	(1351.7, 2088.6)	(1829.6, 2579.3)	(2179, 2498.3)	(5.6, 6.6)	(10.3, 13.1)	(13.1, 15.9)	(12.2, 14.7)
			405.9	592.8	590.2	805.7	4.2	6.2	5.9	7.8
Iowa	40	23	(327.9, 620.4)	(533.2, 676.4)	(512.1, 715.4)	(794.8, 812.6)	(3.8, 4.5)	(5.8, 6.5)	(5.6, 6.2)	(7.4, 8.1)
			236	199.6	361.8	663.7	2.2	2.2	3.9	6.2
Idaho	49	45	(178.2, 346.3)	(177.5, 219.4)	(307.9, 443.6)	(653.7, 670.5)	(2, 2.4)	(2, 2.4)	(3.7, 4.1)	(5.8, 6.5)
			942.8	859.5	1019.6	990.6	7.1	6.8	7.2	7
Illinois	31	40	(833, 1149.9)	(785.2, 961.6)	(920.1, 1133.3)	(952.3, 1018.8)	(6.3, 7.8)	(6.3, 7.1)	(6.7, 7.7)	(6.7, 7.2)
			1944.7	1086.9	1498.7	1033.1	12.9	8.1	10.9	7.7
Indiana	22	26	(1667.6, 2450.8)	(999.6, 1196.5)	(1333.8, 1702.8)	(1013.8, 1048.1)	(11.4, 14.5)	(7.4, 8.6)	(9.8, 11.8)	(7.3, 8.1)
			319.9	891	1257.9	751.6	3.1	8.4	11.9	6.8
Kansas	43	35	(262.9, 428.1)	(811.2, 1006.7)	(1134.2, 1398.1)	(739.9, 761)	(2.9, 3.4)	(7.6, 8.9)	(10.8, 12.8)	(6.4, 7)
			2287.8	1378.3	518.9	1104	17.5	11.9	5	10.2
Kentucky	16	5	(1952.8, 2881.8)	(1259.9, 1533.7)	(435.7, 636.2)	(1088.1, 1115.5)	(15.3, 19.9)	(10.8, 12.7)	(4.7, 5.3)	(9.7, 10.7)
			954.3	1083.5	956.9	1277.8	8.5	9.7	7.9	10.7
Louisiana	11	6	(802.1, 1213.1)	(1006.7, 1165.5)	(852.5, 1113)	(1237.7, 1315.7)	(7.8, 9.2)	(8.9, 10.2)	(7.4, 8.4)	(10.3, 11.2)
			1024.7	1169	1227.5	1151.2	8	9	9.6	8.2
Massachusetts	12	19	(894.6, 1304.4)	(1049.1, 1330.4)	(1093.9, 1416.1)	(1114.8, 1179.5)	(7.3, 8.7)	(8.4, 9.4)	(8.9, 10.3)	(7.8, 8.5)
Maryland	29	44	1037.8	870.9	1181.3	1069.2	6.8	5.6	7.6	6.2

© 2025 Sahu M et al. JAMA Health Forum.

			(934, 1236.2)	(804.7, 949.7)	(1063.1, 1364.4)	(1016.7, 1108.7)	(6.1, 7.5)	(5.2, 5.8)	(7, 8.1)	(6, 6.5)
			588.5	682.4	964.7	999.2	4.8	5.3	6.3	8.4
Maine	14	7	(406.6, 900.5)	(578.7, 988.6)	(710.7, 1367.8)	(990.2, 1005.2)	(4.3, 5.3)	(5, 5.6)	(5.8, 6.7)	(8, 8.7)
			1002.8	939.7	1393.2	1027.2	7.3	7.7	10	7.8
Michigan	21	22	(868.7, 1284.4)	(861.8, 1044.9)	(1219.8, 1637.5)	(1007.2, 1043.1)	(6.4, 8)	(7.1, 8.1)	(9.1, 10.7)	(7.4, 8.1)
			1038.8	1349.7	1207	767.8	8.5	11.4	9.5	6.3
Minnesota	41	43	(909.1, 1329.6)	(1228.1, 1511.5)	(1019.2, 1469.4)	(744.1, 781.2)	(7.5, 9.3)	(10.3, 12)	(8.6, 10.3)	(6, 6.6)
			930.4	1059.8	1856.3	1097.1	7.6	8.9	13.4	8.9
Missouri	17	13	(785.2, 1319.3)	(968.3, 1193.8)	(1591.4, 2204.2)	(1073.1, 1115.6)	(6.9, 8.4)	(8.1, 9.4)	(12, 14.6)	(8.4, 9.2)
			536	905.6	578.9	1166.7	4.4	8.6	5	10.4
Mississippi	20	9	(432.8, 781.4)	(851.3, 965.3)	(480.4, 742.6)	(1130.4, 1199.5)	(4.1, 4.9)	(8, 9)	(4.7, 5.3)	(9.9, 10.8)
			102.2	256	387.6	581	1.2	2.4	3.2	5.5
Montana	50	46	(88.2, 113)	(228.9, 280.5)	(310.5, 556.7)	(575.7, 585.8)	(1.1, 1.3)	(2.2, 2.6)	(2.9, 3.4)	(5.1, 5.7)
			1475.8	993.6	1669.4	1195.9	10.7	7.7	12.5	8.9
North Carolina	13	18	(1314.3, 1863.6)	(906.3, 1112.4)	(1468.5, 1899.5)	(1158, 1229.6)	(9.5, 11.8)	(7.1, 8.1)	(11.4, 13.5)	(8.5, 9.3)
			120.1	628.4	807.8	962.4	1.5	9.1	6.5	8.6
North Dakota	34	20	(111.3, 133.8)	(530.5, 759.2)	(639, 1104.2)	(948.5, 972.4)	(1.4, 1.7)	(8.5, 9.6)	(5.7, 7.1)	(8.1, 9)
			453	588.7	1630.3	1012.2	3.8	5.1	13.1	8.1
Nebraska	30	25	(350.5, 653.6)	(523.7, 656.2)	(1446.8, 1796.7)	(996.8, 1025.6)	(3.5, 4.2)	(4.8, 5.4)	(11.6, 14.3)	(7.6, 8.4)
New Hampshire	28	31	498.2	575.9	698.5	930.5	3.2	4	4.2	6.9
			(371.2, 712.6)	(487.1, 763.6)	(547, 954.1)	(917.9, 938.7)	(2.8, 3.6)	(3.8, 4.2)	(3.9, 4.5)	(6.6, 7.1)
			1211.6	1155.6	1134.3	1358	8	7.6	7.5	7.8
New Jersey	9	29	(1085.5, 1429.9)	(1061.6, 1292.4)	(1045.9, 1236.9)	(1312.6, 1402)	(7.2, 8.7)	(7.1, 8)	(7, 8)	(7.5, 8.1)
			337.4	514.9	640.6	813.7	3.4	5.2	6.3	7.4
New Mexico	46	34	(281.1, 468.4)	(452.7, 577.6)	(607.3, 675.7)	(787.2, 839.2)	(3.1, 3.7)	(4.9, 5.5)	(5.8, 6.7)	(7, 7.6)
Nevada	25	28	817.3	961.2	1152.5	1092	6.3	8.1	9.2	8.2

© 2025 Sahu M et al. JAMA Health Forum.

			(736.2, 966.5)	(865.4, 1117.2)	(1061.3, 1264.4)	(1040.5, 1137.5)	(5.6, 6.8)	(7.6, 8.5)	(8.6, 9.7)	(7.9, 8.5)
New York	2	8	1215.8	1386.9	1428.8	1545.9	9.5	9.6	10.1	9.4
			(1126.1, 1399.7)	(1295.6, 1530.2)	(1332.5, 1553)	(1496.5, 1584)	(8.8, 10.1)	(9, 10.1)	(9.5, 10.7)	(9, 9.8)
Ohio	32	27	810.2	934.6	975.6	891.3	6.5	7.9	7.8	7.4
			(696.1, 1053.2)	(858.9, 1041.2)	(849.9, 1142.5)	(875.3, 903.1)	(5.9, 7.2)	(7.3, 8.3)	(7.1, 8.5)	(7, 7.7)
Oklahoma	24	21	470.3	1184.2	1311.3	1092.7	4.6	9.5	10.5	8.4
			(389.2, 634.9)	(1081.7, 1307.5)	(1173, 1481.5)	(1071.4, 1113.8)	(4.2, 5)	(8.6, 10.1)	(9.6, 11.3)	(8, 8.8)
Oregon	47	42	547.8	576.2	890.3	676.1	5.1	5.4	8.2	6.2
			(484.8, 677)	(496.5, 682.7)	(806.8, 1016.1)	(661.8, 685.6)	(4.6, 5.6)	(5, 5.7)	(7.6, 8.7)	(5.9, 6.5)
Pennsylvania	10	14	1300.4	1076.2	1424.9	1137.9	9.6	8.3	10.7	8.4
			(1147.7, 1631.5)	(980.4, 1204.2)	(1267.4, 1678.2)	(1114.4, 1156.1)	(8.6, 10.6)	(7.7, 8.7)	(9.9, 11.5)	(8, 8.7)
Rhode Island	6	4	830.9	884.9	964.4	1359.4	6.9	7.6	8.6	10.4
			(703.3, 1099.5)	(794.8, 1005.3)	(873.6, 1121.4)	(1329.1, 1379.9)	(6.3, 7.5)	(7.2, 8.1)	(8.1, 9.1)	(9.9, 10.9)
South Carolina	18	17	540.4	1003.4	1436.5	1085.6	3.9	7.9	11.1	8.7
			(418.7, 736.7)	(928.8, 1107.2)	(1234, 1659.5)	(1055.2, 1116.8)	(3.5, 4.3)	(7.2, 8.3)	(10, 12.2)	(8.3, 9)
South Dakota	42	41	411.1	624	485.8	795.8	3.4	13.4	5	6.6
			(327.3, 578.6)	(537.5, 732.1)	(397.3, 660.2)	(782.9, 806.4)	(3, 3.6)	(12.1, 14.6)	(4.8, 5.3)	(6.3, 6.9)
Tennessee	19	15	1836.3	961.3	874.5	1074.8	13.4	8.1	7	9
			(1568.3, 2349.6)	(868.6, 1089.5)	(744.5, 1104.7)	(1045.2, 1097.1)	(11.7, 15.2)	(7.4, 8.6)	(6.3, 7.6)	(8.5, 9.4)
Texas	23	32	1355.9	1011.4	986.2	1337.7	9.9	7.6	7.3	9
			(1237.1, 1600)	(914.5, 1125.4)	(927.5, 1065.5)	(1284.1, 1387.3)	(8.8, 10.9)	(7, 8)	(6.9, 7.7)	(8.6, 9.3)
Utah	38	39	1098.8	537.3	1142.9	1024.4	8.3	4.8	9.2	8.4
			(929.4, 1411.4)	(455.6, 672)	(998.4, 1306.4)	(995, 1045.5)	(7.3, 9.2)	(4.5, 5.1)	(8.4, 10)	(8, 8.7)
Virginia	33	37	1096.6	843.5	1065	954.5	8.6	6.5	7.9	7

© 2025 Sahu M et al. JAMA Health Forum.

			(994.5, 1290.7)	(783.6, 913.8)	(957, 1235.6)	(923.7, 978.1)	(7.7, 9.5)	(6.1, 6.8)	(7.1, 8.6)	(6.8, 7.3)
			209.7	446.9	401.2	748.6	2.2	4.2	3.1	6.5
Vermont	39	33	(193.5, 230.5)	(401.5, 511.9)	(312, 559.3)	(744.6, 751.5)	(2, 2.3)	(3.9, 4.4)	(2.8, 3.3)	(6.1, 6.8)
			607.9	851.4	733.1	679.4	5.3	7	6.5	5.6
Washington	48	51	(535.6, 725.3)	(742.9, 958.3)	(635.6, 858.5)	(658.2, 695.5)	(4.7, 5.8)	(6.4, 7.3)	(6, 7.1)	(5.3, 5.8)
			1437.1	1204.7	1745.1	782.8	10.3	9.2	13.7	6.4
Wisconsin	37	36	(1237.7, 1845.5)	(1093.9, 1340.3)	(1504.9, 2009.9)	(766.9, 796.1)	(9.1, 11.4)	(8.4, 9.7)	(12.3, 14.9)	(6.1, 6.7)
			572.2	668.8	558.7	1212	4.1	6.2	4.1	10.2
West Virginia	7	3	(438.3, 944.5)	(588.7, 807.6)	(449.5, 820.4)	(1205.2, 1216.4)	(3.7, 4.6)	(5.8, 6.6)	(3.8, 4.4)	(9.7, 10.5)
			69.9	165	283.9	618.2	0.9	1.5	2.6	5.7
Wyoming	51	49	(63.8, 75.7)	(151.2, 182.5)	(248.2, 332.4)	(614.4, 621.2)	(0.7, 1)	(1.3, 1.7)	(2.4, 2.8)	(5.3, 6)

© 2025 Sahu M et al. JAMA Health Forum.

B. eFigure 3: *State maps of pharmaceutical spending per capita (age-standardized), 2019*

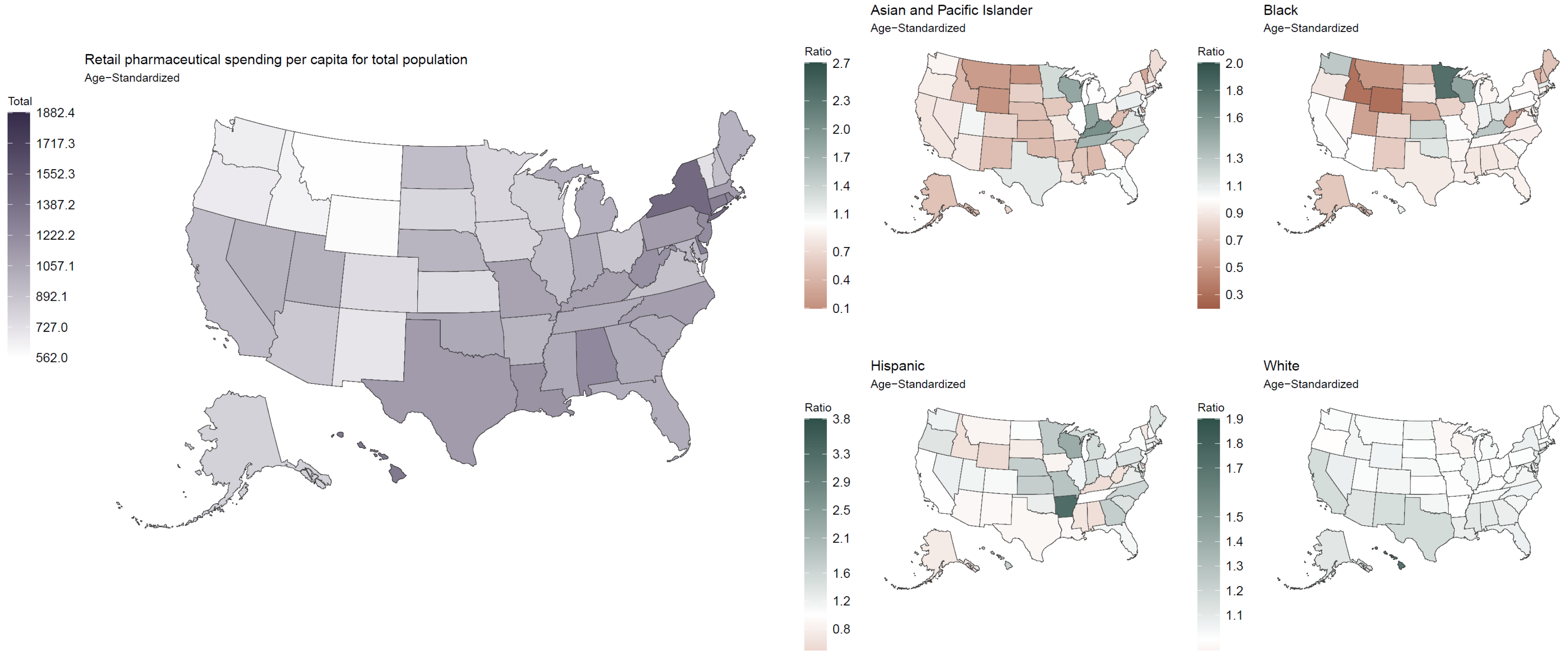

© 2025 Sahu M et al. JAMA Health Forum.

C. eFigure 4: *State-level decomposition (aggregated across 52 health conditions)*

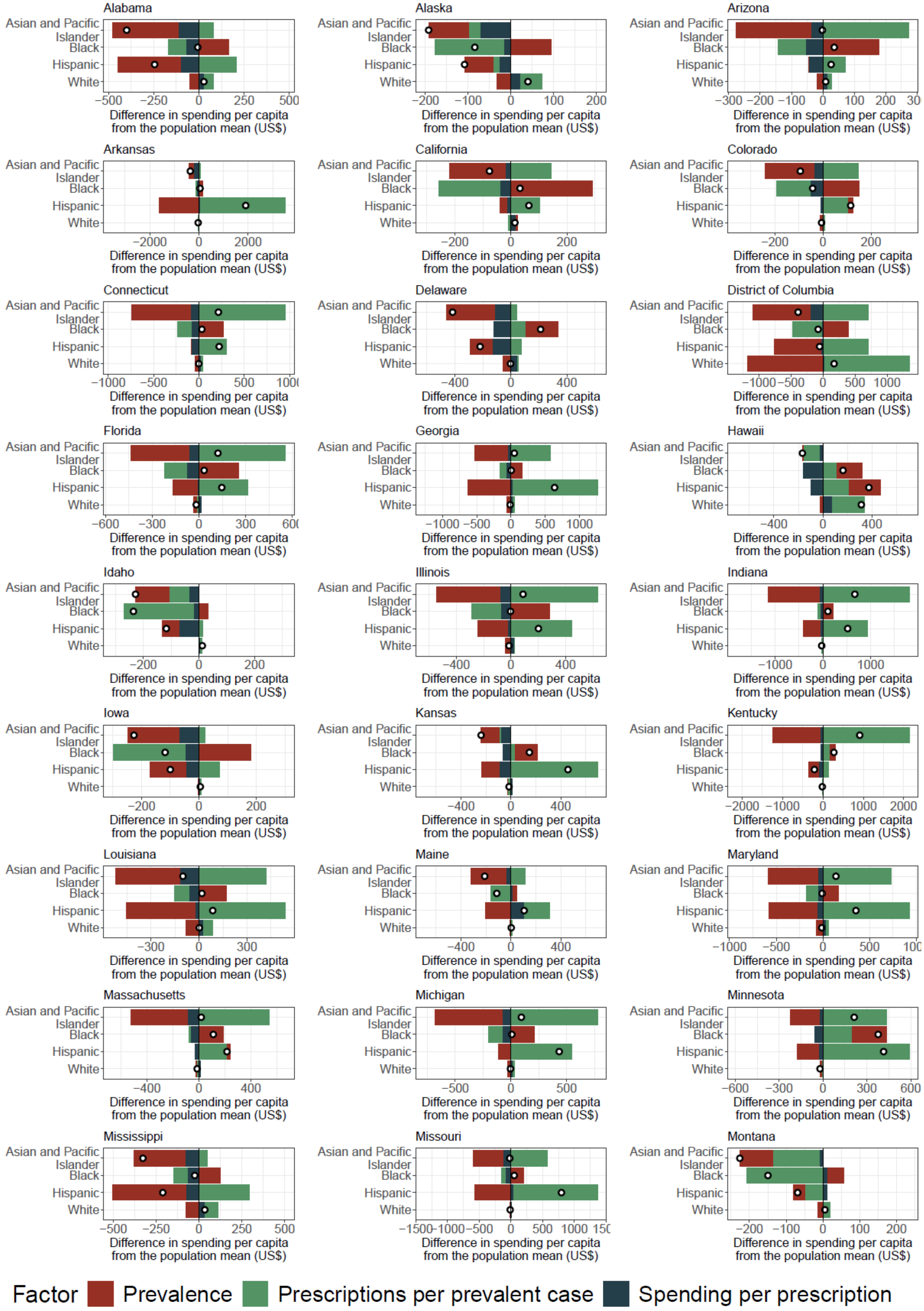

© 2025 Sahu M et al. JAMA Health Forum.

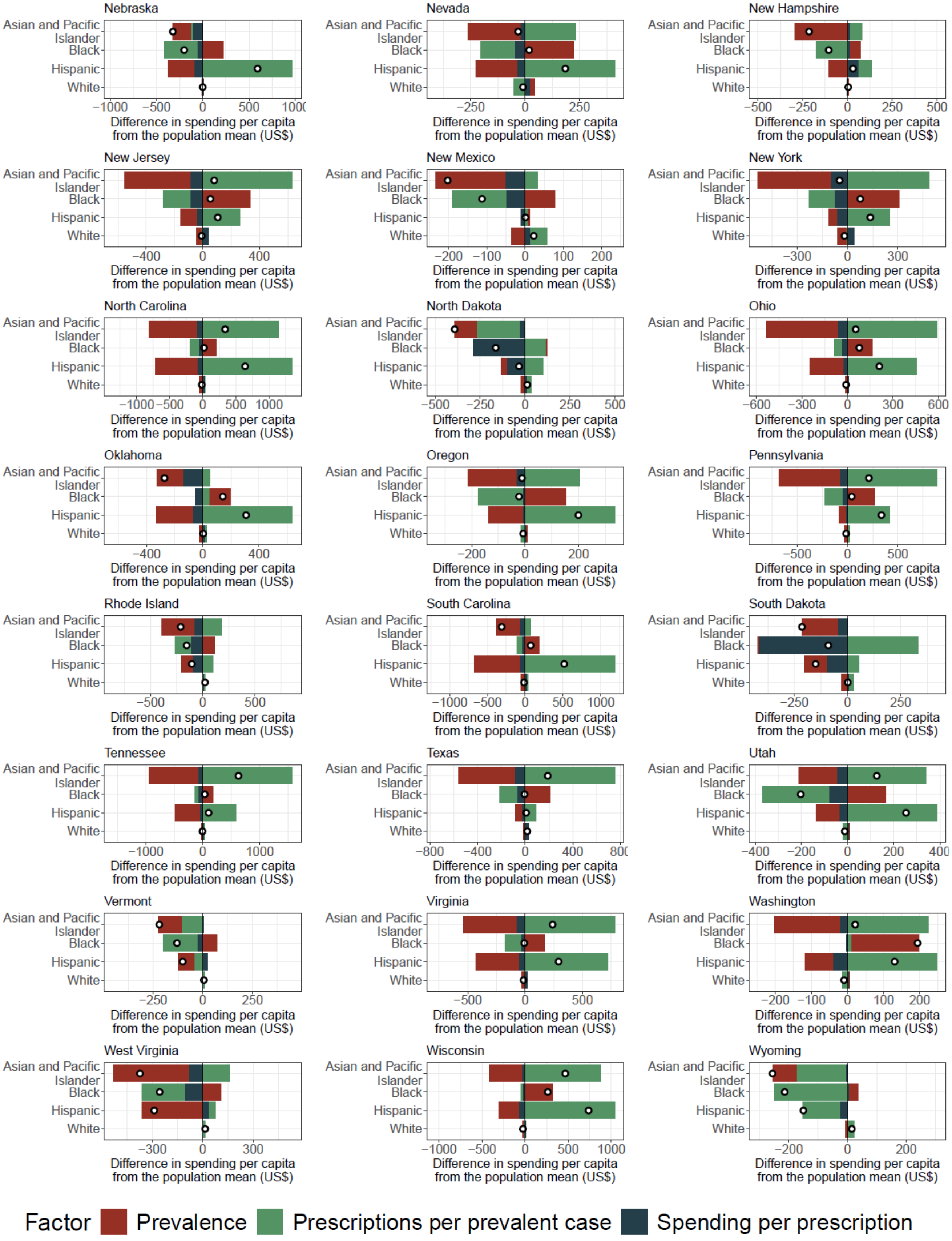

© 2025 Sahu M et al. JAMA Health Forum.

eAppendix 5. GATHER

This study complies with the Guidelines for Accurate and Transparent Health Estimates Reporting (GATHER) recommendations. We have documented the steps involved in our analytical procedures and detailed the data sources used. See Table S5.1 for the GATHER checklist. The GATHER recommendations can be found here: http://gather-statement.org/

Table S5.1 Gather checklist

#	GATHER checklist item	Description of compliance	Reference
Objectives and funding			
1	Define the indicators, populations, and time periods for which estimates were made.	Narrative provided in paper and methods appendix describing indicators, definitions, and populations	Main text (Methods) and methods appendix
2	List the funding sources for the work.	Funding sources listed in paper	Main text (Acknowledgments)
Data Inputs			
For all data inputs from multiple sources that are synthesized as part of the study:			
3	Describe how the data were identified and how the data were accessed.	Narrative provided in paper and methods appendix describing data-seeking methods	Main text (Methods) and methods appendix
4	Specify the inclusion and exclusion criteria. Identify all ad-hoc exclusions.	Narrative provided in paper and methods appendix describing inclusion and exclusion criteria	Main text (Methods) and methods appendix
5	Provide information on all included data sources and their main characteristics. For each data source used, report reference information or contact name/institution, population represented, data collection method, year(s) of data collection, sex and age range, diagnostic criteria or measurement method, and sample size, as relevant.	Metadata for data sources by component, activity, geography, currency, currency year, and income classification will be available through an interactive, online data record	Link to the GHDx to be provided upon publication.
6	Identify and describe any categories of input data that have potentially important biases (e.g., based on characteristics listed in item 5).	Summary of known biases included in paper narrative	Main text (Limitations)
For data inputs that contribute to the analysis but were not synthesized as part of the study:			

© 2025 Sahu M et al. JAMA Health Forum.

7	Describe and give sources for any other data inputs.	Will be included in GHDx link	Link to the GHDx to be provided upon publication.
For all data inputs:			
8	Provide all data inputs in a file format from which data can be efficiently extracted (e.g., a spreadsheet as opposed to a PDF), including all relevant meta-data listed in item 5. For any data inputs that cannot be shared due to ethical or legal reasons, such as third-party ownership, provide a contact name or the name of the institution that retains the right to the data.	Downloads of input data available through online tools such as the Global Health Data Exchange website	Online data visualization tools and the Global Health Data Exchange, http://ghdx.healthdata.org
Data analysis			
9	Provide a conceptual overview of the data analysis method. A diagram may be helpful.	Write ups of the overall methodological processes, as well as cause-specific modeling processes, have been provided	Main text (Methods) and methods appendix
10	Provide a detailed description of all steps of the analysis, including mathematical formulae. This description should cover, as relevant, data cleaning, data pre-processing, data adjustments and weighting of data sources, and mathematical or statistical model(s).	Corresponding methodological write-ups have been provided	Main text (Methods) and methods appendix
11	Describe how candidate models were evaluated and how the final model(s) were selected.	Details on evaluation of model performance have been provided	Methods appendix
12	Provide the results of an evaluation of model performance, if done, as well as the results of any relevant sensitivity analysis.	Details on evaluation of model performance have been provided	Methods appendix
13	Describe methods for calculating uncertainty of the estimates. State which sources of uncertainty were, and were not, accounted for in the uncertainty analysis.	Details on uncertainty calculations have been provided	Methods appendix

© 2025 Sahu M et al. JAMA Health Forum.

14	State how analytic or statistical source code used to generate estimates can be accessed.	Access statement provided	Code is provided in an online repository, code will be provided upon publication.
Results and Discussion			
15	Provide published estimates in a file format from which data can be efficiently extracted.	Results are available through the Global Health Data Exchange	Link to the GHDx to be provided upon publication.
16	Report a quantitative measure of the uncertainty of the estimates (e.g. uncertainty intervals).	Uncertainty intervals are provided with all results	Main text, methods appendix, and online data tools (the Global Health Data Exchange, link to the GHDx to be provided upon publication.)
17	Interpret results in light of existing evidence. If updating a previous set of estimates, describe the reasons for changes in estimates.	Discussion of methodological differences between our estimates and other available evidence provided in the paper and methods appendix	Main text (Methods and Discussion) and methods appendix
18	Discuss limitations of the estimates. Include a discussion of any modelling assumptions or data limitations that affect interpretation of the estimates.	Discussion of limitations was provided	Main text (Limitations) and methods appendix

© 2025 Sahu M et al. JAMA Health Forum.

eReferences

1. Dieleman JL, Beauchamp M, Crosby SW, et al. Tracking US Health Care Spending by Health Condition and County. *JAMA*. Published online February 14, 2025. doi:10.1001/jama.2024.26790

2. Beauchamp M, Thomson A, et al. Tracking US State Health Spending by Race and Ethnicity. *[Under review]*.

3. Centers for Medicare & Medicaid Services (CMS). Health Expenditures by State of Residence, 1991-2020. Accessed May 10, 2024. https://www.cms.gov/data-research/statistics-trends-and-reports/national-health-expenditure-data/state-residence

4. Merative. MarketScan Research Databases. Accessed January 22, 2025. https://www.merative.com/documents/merative-marketscan-research-databases

5. Agency for Health care Research and Quality. Medical Expenditure Panel Survey (MEPS). Accessed November 12, 2020. https://www.meps.ahrq.gov/mepsweb/

6. Centers for Medicare & Medicaid Services. *Part D Event (PDE) File*. ResDAC Research Data Assistance Center Accessed April 4, 2025. https://resdac.org/cms-data/files/pde

7. Centers for Medicare & Medicaid Services. *TAF Technical Documentation: Claims Files*. MACBIS Medicaid and CHIP Business Information Solutions; 2021. Accessed April 4, 2025. https://resdac.org/sites/datadocumentation.resdac.org/files/2021-08/TAF_TechGuide_Claims_Files.pdf

8. Kythera Labs, Inc. *Deciphering Healthcare*.; 2020. Accessed February 25, 2023. https://www.kytheralabs.com/

9. Health Care Cost Institute. *HCCI 2.0 Commercial Claims*. Accessed February 25, 2023. https://healthcostinstitute.org/data-access-hub

10. US Office of Management and Budget (OMB). 1977 Statistical Policy Directive No. 15: Race and Ethnic Standards for Federal Statistics and Administrative Reporting. Published online May 1978. Accessed April 4, 2025. https://www2.census.gov/about/ombraceethnicityitwg/1978-statistical-policy-handbook.pdf

11. US Census Bureau. Census Bureau Releases 2020 Census Population for More Than 200 New Detailed Race and Ethnicity Groups. Published online September 21, 2023. Accessed April 4, 2025. https://www.census.gov/library/stories/2023/09/2020-census-dhc-a-race-overview.html

12. Agency for Healthcare Research and Quality. Medical Expenditure Panel Survey 2018 Full Year Consolidated Data File. Published online August 2020. Accessed March 22, 2023. https://www.meps.ahrq.gov/data_stats/download_data/pufs/h209/h209doc.shtml#Demographic253

13. Jarrín OF, Nyandege AN, Grafova IB, Dong X, Lin H. Validity of Race and Ethnicity Codes in Medicare Administrative Data Compared With Gold-standard Self-reported Race Collected During Routine Home Health Care Visits. *Medical Care*. 2020;58(1):e1-e8.

14. Filice CE, Joynt KE. Examining Race and Ethnicity Information in Medicare Administrative Data. *Medical Care*. 2017;55(12):e170-e176. doi:10.1097/MLR.0000000000000608

15. Grafova IB, Jarrín OF. Beyond Black and White: Mapping Misclassification of Medicare Beneficiaries Race and Ethnicity. *Med Care Res Rev*. 2021;78(5):616-626. doi:10.1177/1077558720935733

© 2025 Sahu M et al. JAMA Health Forum.

16. Medicaid and CHIP Payment and Access Commission (MACPAC). *Availability of Race and Ethnicity Data for Medicaid Beneficiaries*.; 2022. Accessed February 25, 2023. https://www.macpac.gov/wp-content/uploads/2022/03/MACPAC-brief_Race-and-Ethnicity-Data-Availability.pdf

17. Nead KT, Hinkston CL, Wehner MR. Cautions When Using Race and Ethnicity in Administrative Claims Data Sets. *JAMA Health Forum*. 2022;3(7):e221812. doi:10.1001/jamahealthforum.2022.1812

18. Mokdad AH, Bisignano C, Hsu JM, et al. The burden of diseases, injuries, and risk factors by state in the USA, 1990–2021: a systematic analysis for the Global Burden of Disease Study 2021. *The Lancet*. 2024;404(10469):2314-2340. doi:10.1016/S0140-6736(24)01446-6

19. Dwyer-Lindgren L, Kendrick P, Kelly YO, et al. Cause-specific mortality by county, race, and ethnicity in the USA, 2000–19: a systematic analysis of health disparities. *The Lancet*. 2023;402(10407):1065-1082. doi:10.1016/S0140-6736(23)01088-7

20. US Census Bureau. American Community Survey, 2009–19 American Community Survey 1-Year Estimates, Variable: Health insurance coverage status by age for race and ethnicity groups. Tables B27001B-B27001E, B27001H, B27001I. Accessed using tidycensus package. Accessed June 13, 2024. https://CRAN.R-project.org/package=tidycensus

21. State Health Compare, State Health Access Data Assistance Center (SHADAC). *SHADAC Analysis of American Community Survey (ACS) Public Use Microdata Sample (PUMS) Files*. University of Minnesota Accessed April 24, 2024. statehealthcompare.shadac.org

22. *Race, Ethnicity, and Language Data: Standardization for Health Care Quality Improvement*. National Academies Press; 2009:12696. doi:10.17226/12696

23. US Census Bureau. American Community Survey, 2009–2017 American Community Survey 5-Year Estimates. Tables AJ6XE001-AJ6XE031 using Census data portal. Accessed October 21, 2021. https://data.census.gov/cedsci/

24. US Census Bureau. Decennial Census, 2000, 2010. Tables H9X001-H9X049; using Census data portal. Accessed February 21, 2021. https://data.census.gov/cedsci/

25. Kristensen K, Nielsen A, Berg CW, Skaug H, Bell BM. **TMB** : Automatic Differentiation and Laplace Approximation. *J Stat Soft*. 2016;70(5). doi:10.18637/jss.v070.i05

26. Health Expenditures by State of Residence, 1991-2020. Accessed July 20, 2023. https://www.cms.gov/research-statistics-data-and-systems/statistics-trends-and-reports/nationalhealthexpenddata/nationalhealthaccountsstatehealthaccountsresidence

27. Centers for Medicare & Medicaid Services. National Health Expenditure Accounts: Methodology Paper, 2022: Definitions, Sources, and Methods. Accessed October 10, 2024. https://www.cms.gov/files/document/definitions-sources-and-methods.pdf

28. Dickman SL, Gaffney A, McGregor A, et al. Trends in Health Care Use Among Black and White Persons in the US, 1963-2019. *JAMA Netw Open*. 2022;5(6):e2217383. doi:10.1001/jamanetworkopen.2022.17383

© 2025 Sahu M et al. JAMA Health Forum.
