# Supplementary material for: Prescription Drug Utilization and Spending by Race, Ethnicity, Payer, Health Condition, and US State
Source: JAMA Health Forum. 2025 Aug 8;6(8):e252329. doi: 10.1001/jamahealthforum.2025.2329 (PMC12334964; doi:10.1001/jamahealthforum.2025.2329)
Supplement: Supplement 2. — Data Sharing Statement [file jamahealthforum-e252329-s002.pdf]

Data Sharing Statement

Sahu. Prescription Drug Utilization and Spending by Race, Ethnicity, Payer, Health Condition, and US State. *JAMA Health Forum*. Published August 08, 2025.
doi:10.1001/jamahealthforum.2025.2329

Data
Data available: Yes
Data types: Data (not involving human participants)
How to access data: Input data for this study came from several sources. To download US Health Disparities project estimates of life expectancy at birth, visit the Global Health Data Exchange (https://ghdx.healthdata.org/record/ihme-data/united-states-causes-death-life-expectancy-by-county-race-ethnicity-2000-2019). To download estimates of US state-level health spending and utilization, please visit: https://ghdx.healthdata.org/record/ihme-data/united-states-health-spending-by-state-payer-type-service-2003-2019.
When available: With publication

Additional Information
Who can access the data: Researchers whose proposed use of the data has been approved
Types of analyses: For a specified purpose
Mechanisms of data availability: After approval of a proposal and a signed data access agreement
